# Distinct millisecond-scale click-train patterns evoke discriminable behavioral and neural responses in humans and rats

**DOI:** 10.1371/journal.pbio.3004046

**Published:** 2026-10-08

**Authors:** Yuying Zhai, Peirun Song, Ana Belén Lao-Rodríguez, Xinyu Du, Haoxuan Xu, Hangting Ye, Xuehui Bao, Ishrat Mehmood, Nayaab Shahir Pandit, Cheng Chang, Zhouyou Dai, Zhiyi Tu, Pei Chen, Tingting Zhang, Lingling Zhang, Xuan Zhao, David Pérez-González, Manuel S. Malmierca, Xiongjie Yu

**Affiliations:** 1 Department of Anesthesiology, Shanghai Tenth People’s Hospital, Tongji University School of Medicine, Shanghai, China; 2 Department of Anesthesia, Women’s Hospital, Zhejiang University School of Medicine, Hangzhou, China; 3 Zhejiang Provincial Key Laboratory of Precision Diagnosis and Therapy for Major Gynecological Diseases, Women’s Hospital, Zhejiang University School of Medicine, Hangzhou, China; 4 College of Biomedical Engineering and Instrument Science, Zhejiang University, Hangzhou, China; 5 Key Laboratory for Biomedical Engineering of Ministry of Education, Zhejiang University, Hangzhou, China; 6 Cognitive and Auditory Neuroscience Laboratory (Lab 1), Institute of Neuroscience of Castilla y León (INCYL), University of Salamanca, Salamanca, Spain; 7 Institute for Biomedical Research of Salamanca (IBSAL), University of Salamanca, Salamanca, Spain; 8 Department of Cell Biology and Pathology, Faculty of Medicine, University of Salamanca, Salamanca, Spain; 9 Department of Basic Psychology, Psychobiology and Methodology of Behavioral Sciences, Faculty of Psychology, University of Salamanca, Salamanca, Spain; University College London, UNITED KINGDOM OF GREAT BRITAIN AND NORTHERN IRELAND

## Abstract

Natural sounds are shaped by both spectral content and temporal patterning. Here we ask whether click trains with the same mean inter-click interval, but different millisecond-scale temporal patterns evoke distinct behavioral and neural responses. Human listeners discriminated several such click-train patterns, and human EEG revealed context-dependent deviance-related responses. Awake rats showed analogous cortical response differences at the ECoG level. Along the IC–MGB–A1 pathway, unit recordings revealed weak oddball/context sensitivity in IC, intermediate sensitivity in MGB, and stronger stimulus-specific adaptation in A1. Reversible inactivation of the auditory cortex reduced MGB sensitivity, indicating a contribution of corticothalamic feedback. Further A1 layer analyses showed stronger effects in supragranular than deep cortical layers. Together, these results show that distinct click-train temporal patterns are differentially represented across the auditory system, with stronger sensitivity emerging in higher auditory structures.

## Abstract 2

Natural sounds contain both spectral and temporal information, but how millisecond-scale temporal patterns are represented across the auditory system is unclear. This study shows that distinct click-train patterns evoke discriminable behavioral and neural responses, with sensitivity increasing from midbrain to cortex and shaped by corticothalamic feedback and cortical hierarchy.

## Introduction

Temporal integration provides one key mechanism by which the auditory system transforms discrete acoustic events into stable perceptual objects. When inter-click intervals (ICIs) in a click train are relatively long, listeners perceive individual clicks as separate events. As ICIs shorten below 30 ms, the clicks perceptually fuse into a continuous, pitch-like sound determined by the mean repetition rate—a phenomenon often described as temporal integration or temporal merging [1–7]. This fusion suggests that when ICIs are sufficiently short, the auditory system forms a unitary “temporal object” whose identity depends on the configuration of intervals, rather than on any single event. While it is known that different millisecond-scale temporal configurations—such as periodic versus Poisson click trains—are perceived differently and can be represented with high temporal fidelity in the auditory nerve and early auditory pathway, it remains less clear how sensitivity to these rapidly varying temporal patterns is expressed at higher levels of the auditory system. Specifically, it is unclear how changes in such patterns are reflected in automatic deviance-related responses and how context-dependent sensitivity varies along the ascending auditory pathway.

Click trains, composed of identical brief pulses arranged over time, are therefore an attractive tool for probing temporal processing in the auditory system [6–10]. Most previous work has focused on the encoding of individual clicks and overall rate—examining temporal and rate coding as ICI varies [2,11]—rather than treating the click train as a unified temporal structure with its own identity. Building on the fusion phenomenon described above, a regular click train with constant ICI can be viewed as a single auditory object defined by its temporal configuration [4,5]. This motivates the broader hypothesis that millisecond-scale temporal patterning within click trains may provide a useful basis for perceptual and neural discrimination, not only for regular but also for irregular click trains, and thereby contribute to sound identity and auditory object formation.

In the present study, we test this hypothesis using click trains that share the same average ICI but differ in their temporal configurations. First, we show that human listeners perceive click trains with different temporal configurations as distinct sounds, and that changes in temporal configuration elicit robust mismatch negativity (MMN) responses when presented as deviants in an oddball paradigm, indicating automatic deviance detection at the cortical level. We then demonstrate that rats exhibit context-dependent cortical response differences to changes in click-train temporal pattern, suggesting that neural sensitivity to these stimulus classes can be observed in both humans and rats. Finally, combining these behavioral and electrophysiological findings with pathway-level recordings along the rat inferior colliculus–medial geniculate body–auditory cortex (IC–MGB–A1) axis, we identify where temporal configuration sensitivity emerges along the ascending auditory pathway and provide initial insights into its underlying circuitry. Together, these results reveal how click-train temporal patterns are represented across multiple levels of the auditory system and provide new insight into its neural implementation across species.

## Results

### Sensitivity to temporal configuration in humans

The human auditory system exhibits robust temporal integration: when the ICI in a click train is sufficiently brief, individual clicks are no longer perceived as separate events but fuse into a single auditory object. Previous work has shown that listeners typically fail to detect gaps when the ICI falls below 30 ms [6,9], a phenomenon we refer to as temporal merging. The resulting perceptual unit can be regarded as a temporally merged sound object. Here, we asked whether the temporal configuration within such temporally merged objects influences auditory perception.

We designed a stimulus set comprising 10 distinct click trains (Fig 1A and S1 Table), each containing 51 pulses with a mean ICI of 4 ms. One set, labeled Con, had a constant ICI of 4 ms. A second set, labeled Asc, had ICIs that increased progressively from 2 ms to 6 ms, whereas a third set, labeled Dec, had ICIs that decreased from 6 ms to 2 ms. These labels refer to the direction of change in ICI, not to perceived pitch direction: because repetition rate is inversely related to ICI, Asc corresponds to a descending repetition-rate/pitch trend, whereas Dec corresponds to an ascending repetition-rate/pitch trend. TW_10_ (*two-way*, 10%) consisted of click trains in which each ICI was randomly selected as either 3.6 ms or 4.4 ms, corresponding to 4 ms ± 10%. TW_30_, TW_50_, and TW_70_ were constructed similarly, with each ICI randomly taking one of two values corresponding to 4 ms ± 30%, 4 ms ± 50%, and 4 ms ± 70%, respectively. We also generated three random-pattern sets, RN_10_, RN_40_, and RN_60_, in which ICIs were randomly drawn from continuous ranges corresponding to ±10%, ±40%, and ±60% around 4 ms. The Asc and Dec stimuli therefore represented ordered monotonic interval patterns, the TW stimuli represented two-way interval patterns, and the RN stimuli represented random interval patterns with different degrees of spread. These stimulus classes were chosen to sample several qualitatively different forms of millisecond-scale temporal pattern variation under a shared mean inter-click interval, thereby enabling structured behavioral and neural comparisons across distinct temporal-pattern categories. Because all ICIs were very short (≤7 ms), these click trains were designed to fall within the temporally merged regime examined in this study. Accordingly, the stimuli differed in their inter-click timing patterns and exhibited distinct model-based peripheral auditory representations after cochlear filtering. The audio files of the click-train stimuli are provided in S1 Audio, and the corresponding cochleagrams generated using the auditory model are shown in S1 Fig.

**Fig 1:**
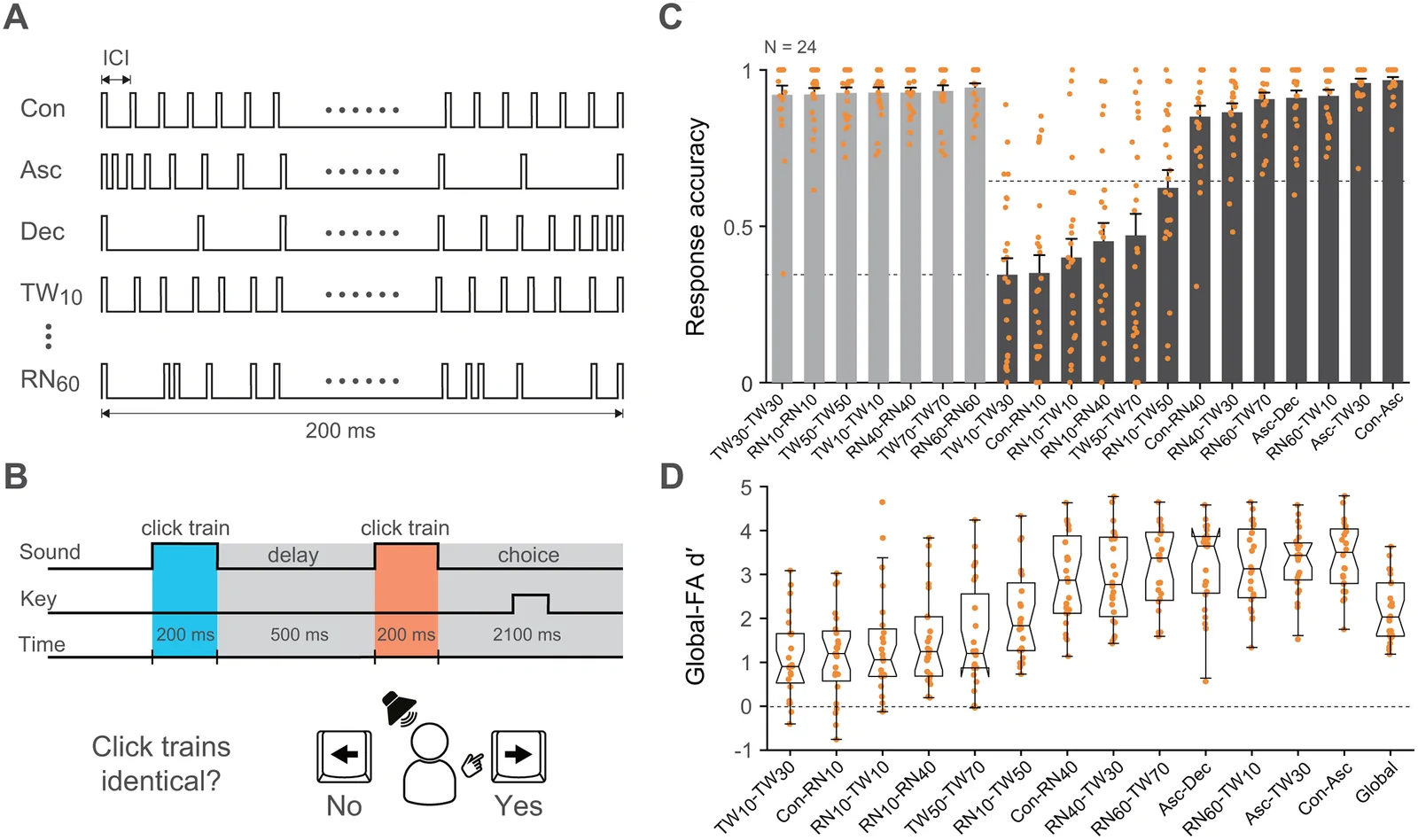
Behavioral discrimination of temporally merged click-train objects. **(A)** Different click-train temporal patterns used for the delayed match-to-sample (DMS) task. Five example sets of click trains are depicted, each comprising 51 pulses with a mean ICI of 4 ms, totaling a duration of 200 ms. Each set is labeled (e.g., Con, Asc), with extensive descriptions available in S1 Table. **(B)** Delayed match-to-sample task setup. Participants were presented with two sequences of click trains separated by a 500-ms interval. They were tasked with determining whether the two sequences were identical by pressing one of two designated buttons (left or right arrow keys). **(C)** Behavioral performance across participants. Response accuracy is shown as the mean correct response rate for each tested click-train pair. Dark gray bars represent different-stimulus pairs, and light gray bars represent identical-stimulus pairs. Error bars indicate the standard error of the mean (SEM). Orange circles denote individual participant accuracies (*N* = 24). Dashed horizontal lines indicate the overall response accuracies expected from consistently choosing the more frequent trial type (“different”, 0.65) or the less frequent trial type (“identical”, 0.35), reflecting the unequal probabilities of the two trial types in the experimental design. **(D)** d′ values calculated from the behavioral responses shown in (C). For each different-stimulus pair, d′ was calculated from the hit rate and false-alarm rate derived from the same/different responses. The hit rate was defined as the proportion of “different” responses for that stimulus pair, whereas the false-alarm rate was estimated from responses to identical-pair trials. For the identical-pair reference, all identical control conditions were included. The “Global” category summarizes d′ values calculated by aggregating responses across all different-stimulus trials and all identical-stimulus trials. Box plots show the distribution of d′ values across participants, with the central line indicating the mean, box edges representing the interquartile range, and whiskers showing the full range. Orange points denote individual participant scores. The horizontal dashed line at d′ = 0 indicates chance-level sensitivity. Source data are available in Zenodo (see Data availability).

To assess perceptual sensitivity to click-train temporal pattern, we used a delayed match-to-sample task in which subjects heard two 200-ms click trains separated by a 500-ms silent interval (Fig 1B). On each trial, the two trains were either identical or drawn from a specific configuration pair, and participants reported whether the two sounds were the “same” or “different” via button press. Twenty-four participants engaged in the task involving 20 pairs of click trains (Fig 1C), and they exhibited similar behavioral response patterns characterized by lower discrimination performance between the TW_10_-TW_30_, Con-RN_10_, and RN_10_-TW_10_ pairs and excellent discrimination between Con-Asc, Asc-TW_30_, RN_60_-TW_10_, Asc-Dec, and RN_60_-TW_70_ pairs. The mean response accuracies for the TW_10_-TW_30_, Con-RN_10,_ and RN_10_-TW_10_ pairs were 0.34, 0.35, and 0.40 (Fig 1C), respectively, indicating that these pairs were difficult to discriminate behaviorally. In contrast, the mean response accuracies for the Con-Asc, Asc-TW_30_, RN_60_-TW_10_, Asc-Dec, and RN_60_-TW_70_ pairs were 0.97, 0.96, 0.92, 0.92, and 0.91, respectively (Fig 1C), all significantly greater than the reference probability of 0.65 corresponding to consistently selecting the more frequent “different” response (*p* < 0.01 for all five pairs, Kolmogorov–Smirnov test), indicating that participants reliably discriminated these stimulus pairs. When both click trains were identical, response accuracies were also high (between 0.911 and 0.933). To quantify discrimination sensitivity while accounting for response bias, we calculated d′ values for each stimulus pair (Fig 1D). This analysis showed a graded pattern of sensitivity across stimulus pairs, with lower d′ values for difficult pairs such as TW_10_–TW_30_, Con–RN_10_, and RN_10_–TW_10_, and higher d′ values for more easily discriminable pairs such as Con–Asc, Asc–TW_30_, RN_60_–TW_10_, Asc–Dec, and RN_60_–TW_70_.

These behavioral results show that, even when click trains share the same mean ICI and duration, differences in click-train temporal patterns can support reliable perceptual discrimination. The graded pattern across stimulus pairs further motivated us to examine how sensitivity to these patterns is represented along the auditory pathway.

To examine whether differences among click-train patterns elicited context-dependent differences between deviant and standard responses, we used several oddball paradigms to assess neural sensitivity to these stimuli. Across four passive EEG sessions, different click-train patterns were assigned as standard and deviant stimuli depending on the experimental design (see Methods, EEG experiment procedures, Passive Listening Sessions 2–5). Figure 2 presents representative examples of these oddball configurations. In the representative configuration shown in Fig 2A, TW_30_ was presented as the standard stimulus, whereas TW_50_, Asc, RN_40_, TW_10_, RN_10_, and RN_60_ served as deviant stimuli. Among these deviant stimuli, TW_50_, Asc, and RN_40_ were selected as representative examples for illustration. Deviant stimuli were randomly interspersed among repetitive standard stimuli, with an average of nine standard stimuli followed by one deviant stimulus at a 300-ms inter-stimulus interval.

**Fig 2:**
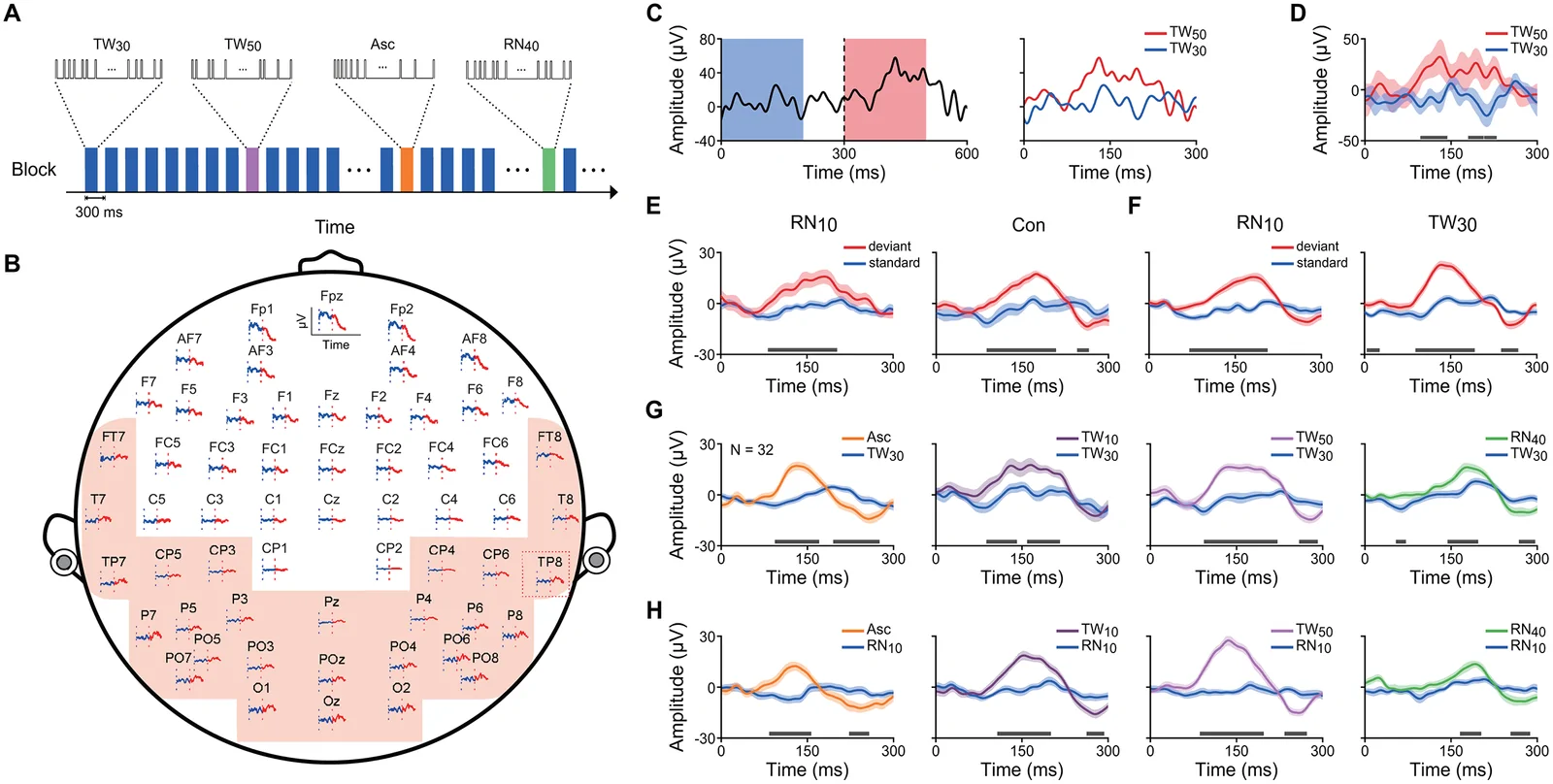
EEG response to oddball paradigm with temporally merged click-train in humans. **(A)** Representative example of an oddball paradigm configuration used in the EEG experiments. In this example, the standard sound (TW_30_) is presented at a 300-ms inter-stimulus interval, followed by a deviant sound after 7-11 standard repetitions. The deviant sound is randomly chosen from a set of stimuli (TW_50_, Asc, RN_40_, TW_10_, RN_10,_ and RN_60_). Different standard-deviant assignments were used across passive EEG sessions (see Methods). **(B)** Topographical layout of the 64-channel EEG setup used for recording human auditory responses. For each channel, individual EEG traces are shown in response to the standard sound (TW_30_, blue) and the subsequent deviant sound (TW_50_, red). Two vertical dotted lines mark the onset of the standard sound (preceding the deviant, blue line) and the onset of the deviant sound (red line), respectively. The shaded background indicates channels that showed robust auditory responses and were selected for further analysis. The red rectangle indicates the channel expanded in panel (C). **(C)** Left panel: enlarged view of the EEG response from the channel indicated by the red rectangle in (B). Right panel: comparative analysis of the EEG responses to the standard and deviant sounds, with alignments based on the onsets of the sounds. **(D)** The averaged responses to the standard and deviant sounds across channels highlighted with the shaded background, for the example participant. Gray bars above the x-axis indicate a significant difference (*p* < 0.05, independent-samples *t* test with FDR correction) between the standard and deviant responses during this period. **(E)** Population-level EEG responses (*N* = 32 subjects) to the stimulus pair RN_10_-Con, showing a contextual comparison in which the same physical stimulus was presented as the standard stimulus (blue) in one oddball session and as the deviant stimulus (red) in another session. Gray bars above the x-axis indicate a significant difference (*p* < 0.05, independent-samples *t* test with FDR correction) between the standard and deviant responses during this period. **(F)** Population-level EEG responses to the stimulus pair RN_10_-TW_30_, showing a similar comparison in which the same physical stimulus was presented under different standard (blue) and deviant (red) roles across oddball paradigms. **(G)** Population-level EEG responses to a representative multi-deviant oddball configuration using TW_30_ as the standard sound (blue) and Asc, TW_10_, TW_50_, and RN_40_ as deviants. **(H)** Population-level EEG responses to a representative multi-deviant oddball configuration using RN_10_ as the standard (blue) and Asc, TW_10_, TW_50_, and RN_40_ as deviant sounds. Source data are available in Zenodo (see Data availability).

We conducted 64-channel electroencephalogram (EEG) recordings (Fig 2B) on 32 subjects to capture the neural responses to these auditory stimuli. In one illustrative case, the deviant response to TW_50_ contrasted sharply with the response to the preceding standard sound TW_30_. Specifically, robust responses were detected to TW_50_, particularly in the occipital and temporal lobes, whereas no obvious response was observed for the preceding standard sound TW_30_ (Fig 2B). An enlarged view of channel TP8 clearly demonstrated the difference (Fig 2C, left panel), with responses aligned to stimulus onset (Fig 2C, right panel). Following our previous study [9], we predefined a region of interest comprising 27 EEG channels over the temporal and occipital regions, where robust auditory responses to click trains had previously been identified. Averaging responses across this predefined region of interest (indicated by the red background in Fig 2B) further clarified the difference between deviant and standard responses, revealing a sustained and statistically significant contrast from approximately 80 ms to 280 ms (*p* < 0.05, deviant versus standard, independent-samples *t* test, with FDR correction applied, Fig 2D).

In our human subject population, significant differences between deviant and standard responses emerged for multiple click-train patterns, particularly within the 100–200 ms post-stimulus window. To examine whether these differences varied with the contextual role of the stimulus, we compared neural responses to the same physical stimulus when it served as a standard stimulus in one passive EEG session and as a deviant stimulus in another session. Figure 2E and 2F show representative examples of such contextual comparisons, in which significant differences between deviant and standard responses were observed for the RN_10_–Con and RN_10_–TW_30_ comparisons, respectively. We then examined the multi-deviant oddball paradigm. Figure 2G and 2H show representative examples in which TW_30_ (Fig 2G) and RN_10_ (Fig 2H) served as the standard stimuli, respectively; the gray horizontal bars indicate time ranges in which the response to the deviant differed significantly from the response to the standard (*p* < 0.05, independent-samples *t* test, with FDR correction applied). Thus, the human auditory cortex automatically detects changes in click-train temporal patterns, generating MMN responses even in the absence of an explicit discrimination task.

### Cross-species sensitivity to temporal configuration in rats

Building on the behavioral and electrophysiological evidence obtained in humans, we next examined whether analogous neural sensitivity to click-train temporal patterns could be observed in awake rats. We used a two-sequence oddball paradigm (Fig 3A), in which each stimulus in a pair of temporally merged click trains was presented once as the standard and once as the deviant. In one sequence, one click train was repeated nine times as the standard, followed by the other click train as the deviant; this pattern of nine standards followed by one deviant was repeated for 30 cycles. In the second sequence, the standard and deviant roles of the two click trains were reversed. For comparison, the response to the standard sound immediately preceding the deviant was selected as the standard response. We then performed 32-channel electrocorticography (ECoG) recordings from the auditory cortex in awake rats (Fig 3B).

**Fig 3:**
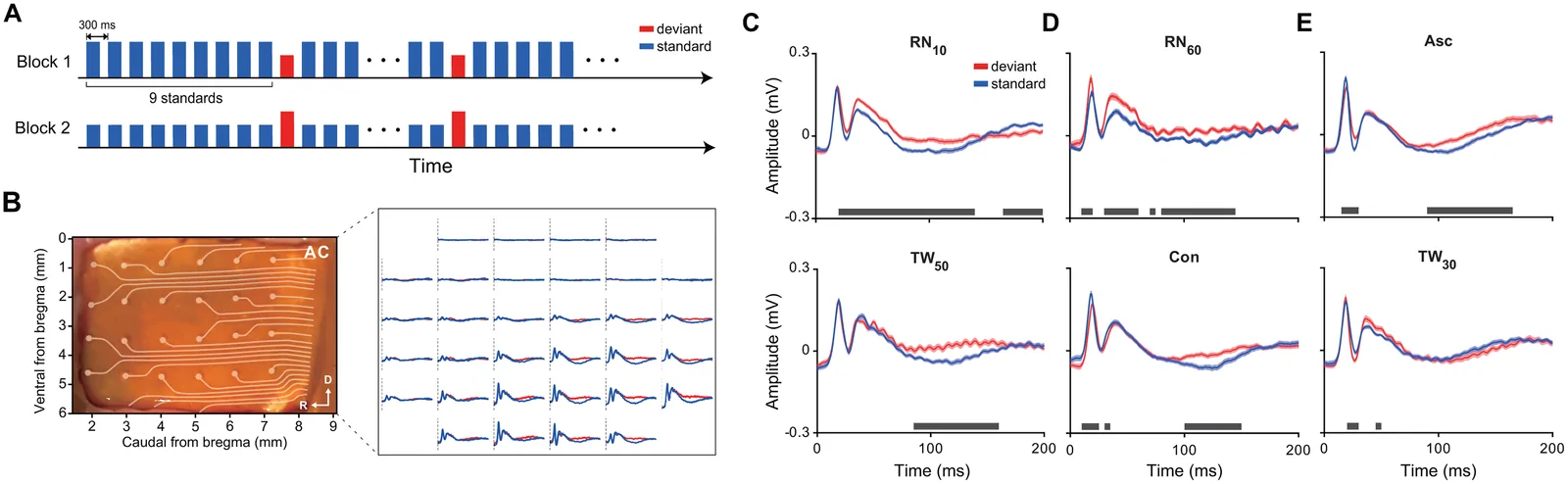
ECoG responses to temporally merged click-train in an oddball paradigm in awake rats. **(A)** Oddball Stimulus Paradigm: the oddball paradigm consists of two sets of stimuli. Each set contains a pair of sounds: one sound is repeatedly presented as the standard sound at a 300-ms inter-stimulus interval, followed by a deviant sound after every nine standard sounds. This sequence (9 standard sounds followed by 1 deviant) is repeated 30 times. In the other set, the roles of the standard and deviant sounds are reversed. **(B)** Left panel: photograph of the rat auditory cortex with an overlaid 32-channel ECoG array used for recording. Right panel: example ECoG waveforms from all 32 channels in response to the same sound (TW_50_) presented as the standard (blue line) and as the deviant (red line). Dashed vertical lines indicate stimulus onset (time zero). **(C–E)** Population-level ECoG responses to the same physical click-train stimuli when presented as standards or deviants in the oddball paradigm illustrated in (A). ECoG channels in each rat were selected based on deviant responses exceeding the baseline amplitude ([−50, 0 ms]) by more than 2 standard deviations within the [0, 200 ms] window (*N* = 3 rats, *n* = 36 channels). **(C)** Responses to the RN_10_–TW_50_ pair. The top row shows responses to RN_10_ presented as standard (blue) and deviant (red), respectively. Bottom row shows responses to TW_50_ as standard (blue) and deviant (red). Shaded areas indicate SEM across all selected channels. Gray bars above the x-axis indicate time windows with significant standard–deviant differences (*p* < 0.05, independent-samples *t* test with FDR correction) between the standard and deviant responses during this period. **(D)** Responses to the RN_60_–Con pair. Top row: RN_60_ as standard and deviant. Bottom row: Con as standard and deviant. **(E)** Responses to the Asc–TW_30_ pair. Top row: Asc as standard and deviant. Bottom row: TW_30_ as standard and deviant. Source data are available in Zenodo (see Data availability).

Across rats, auditory cortical ECoG responses differed between standard and deviant presentations when the same physical click-train stimulus was compared across its two roles. However, the direction and time course of these deviant–standard differences varied across stimulus conditions, and deviant responses were not uniformly larger or more negative than standard responses. For example, RN_10_ and TW_50_ showed significant context-dependent differences between deviant and standard responses when the same stimuli were presented as standards or deviants (Fig 3C). Similar context-dependent differences were also observed for additional stimulus pairs, including RN_60_–Con and Asc–TW_30_ (Fig 3D, 3E). These results indicate that click-train temporal patterns can evoke context-dependent cortical population responses in awake rats. Together with the human EEG results, these findings suggest analogous neural sensitivity to click-train temporal patterns across species, although the present data do not establish perceptual equivalence between humans and rats.

### Neuronal mechanisms underlying sensitivity to temporal configuration

Next, we conducted extracellular recordings along the auditory pathway (Fig 4A) to further investigate the underlying neuronal basis of sensitivity to click-train temporal patterns. These recordings include A1 (Fig 4B), medial geniculate body (MGB, Fig 4C), and inferior colliculus (IC, Fig 4C). This approach allowed us to compare context-dependent sensitivity to click-train temporal patterns across multiple auditory levels using a classic two-sequence oddball paradigm, a widely adopted method for assessing stimulus-specific adaptation at the neuronal level [12–23] (Fig 4D – 4F).

**Fig 4:**
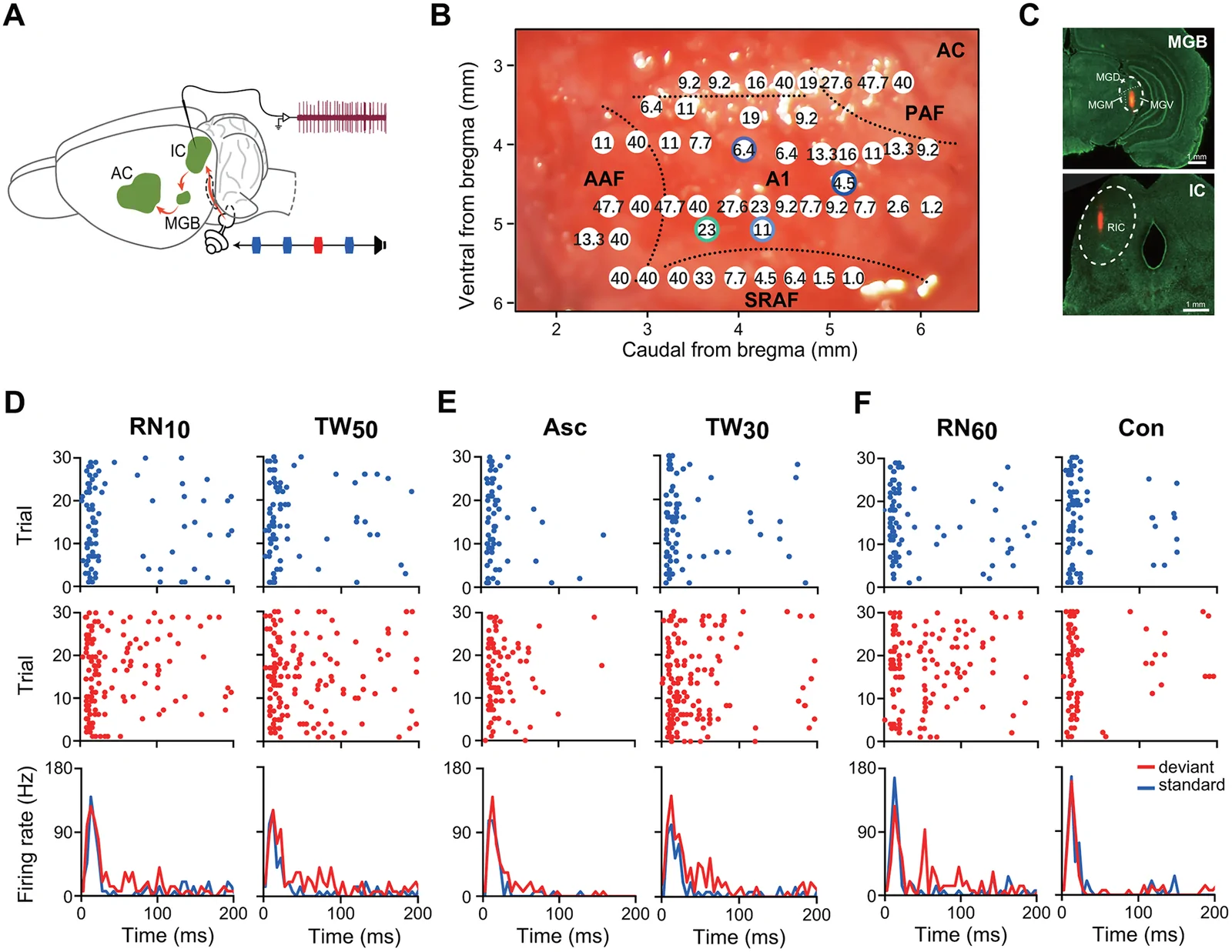
Neuronal response to oddball paradigm with temporally merged click-train. **(A)** Extracellular recording setup: the recording process along the auditory pathway, including the inferior colliculus (IC), medial geniculate body (MGB), and primary auditory cortex (A1). Modified from Parras and colleagues (2017). **(B)** Tonotopic distribution of characteristic frequencies (CF) across the auditory cortex of one example rat, with frequencies indicated in kilohertz (kHz). Colored circles mark the locations where neuronal recordings were conducted for the main dataset in A1 in the rat. **(C)** Top panel: localization of the MGB. The orange area highlights the track of the Neuropixels probe within the MGB, as indicated by the thick dashed line. The thin dashed lines delineate the subregions of the MGB, including the medial (MGM), dorsal (MGD), and ventral (MGV) subdivisions. Bottom panel: localization of the IC. The orange area indicates the track of the Neuropixel probe within the IC, outlined by the thick dashed line. Thie electrode was placed in the rostral subdivision of the inferior colliculus (RIC). **(D–F)** Responses of an example A1 neuron in the oddball paradigm. Raster plots display the responses to a sound when it is in the role of standard (top row) and in the role of deviant (middle row), for three stimulus pairs: (D) RN_10_ and TW_50_; (E) Asc and TW_30_; (F) RN_60_ and Con. The bottom row presents the peri-stimulus time histograms (PSTHs) for each sound when presented as the deviant (red) or standard (blue) stimulus. Source data are available in Zenodo (see Data availability).

For one example A1 neuron, we tested three pairs: RN_10_ and TW_50_, Asc and TW_30_, and RN_60_ and Con. In the pair RN_10_ and TW_50_, the A1 neuron showed robust responses in both the standard and deviant conditions, with the deviant response being clearly stronger (raster plots in red, Fig 4D), and significant differences between deviant and standard responses were observed for both trains (RN_10_, *p* = 0.0014; TW_50_, *p* < 0.001; independent-samples *t* test, bottom row in Fig 4D). Similarly, for the pair Asc and TW_30_, we observed analogous responses, with significant differences between deviant and standard responses for both (Asc, *p* < 0.05; TW_30_, *p* < 0.001; independent-samples *t* test, bo*t*tom row in Fig 4E). For the pair RN_60_ and Con, significant differences were observed between deviant and standard responses for RN_60_ (*p* = 0.0102, independent-samples *t* test, bottom row in Fig 4F) but not for Con (*p* = 0.94, independent-samples *t* test, bottom row in Fig 4F).

We further analyzed the differences in neuronal responses across different levels of the auditory pathway for the pairs RN_10_ and TW_50_, RN_60_ and Con, and Asc and TW_30_ (Fig 5), presented in an oddball paradigm. For RN_10_ and TW_50_, the responses in the IC neuronal population (*n* = 940) to deviant and standard sounds overlapped and showed no significant differences (RN_10_, *p* = 0.81; TW_50_, *p* = 0.18; independent-samples *t* test, Fig 5C). However, in the MGB (*n* = 632), deviant responses for both RN_10_ and TW_50_ were significantly stronger than standard responses (RN_10_, *p* = 0.018; TW_50_, *p* = 0.0019; independent-samples *t* test, Fig 5B). Similarly, in the A1 (*n* = 671), deviant responses were significantly stronger than standard responses (RN_10_, *p* = 0.037; TW_50_, *p* < 0.001; independent-samples *t* test, Fig 5A).

**Fig 5:**
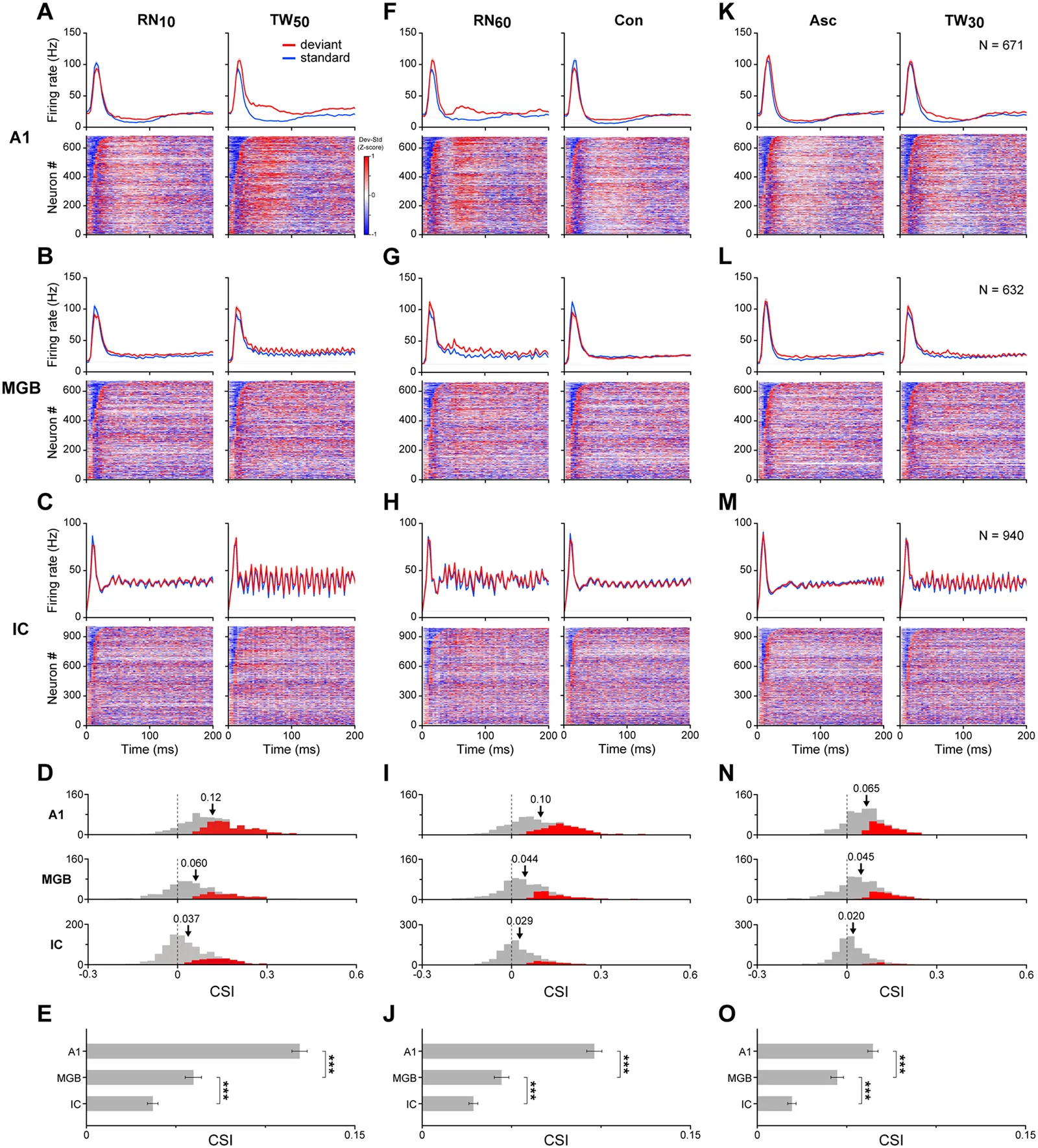
Population neuronal response to oddball paradigm with temporally merged click-train in IC, MGB, and A1. **(A)** Top panels: population PSTHs displaying averaged neuronal responses to RN_10_ (left) and TW_50_ (right) as deviant (red) and standard (blue) stimuli in A1. Bottom panels: heatmaps illustrating the z-scored differences (deviant − standard) in PSTHs of firing rates for each neuron across the population. A diverging colormap centered at 0 was used, with warmer colors indicating positive differences and cooler colors indicating negative differences. Time is aligned to stimulus onset at 0 ms. **(B, C)** Population PSTHs in MGB and IC following the same formatting conventions as A1 (A). **(D)** Distribution of CSI calculated for neurons in A1 (top row), MGB (middle row), and IC (bottom row) within the time window of 5–200 ms from stimulus onset. Red bars highlight neurons with CSI significantly greater than 0, determined by a nonparametric bootstrap procedure with 1,000 iterations (*p* < 0.05). Arrows point to the mean. **(E)** Comparison of CSI values among A1, MGB, and IC. Error bars represent standard error of the mean (SEM). ***: *p* < 0.001, ANOVA with post-hoc test. **(F–J)** Population responses to another sound pair in the oddball paradigm (RN_60_, Con), following the same conventions as panels (A–E). **(K**–**O)** Population responses to yet another sound pair in the oddball paradigm (Asc, TW_30_), adhering to the same conventions as panels (A–E). Source data are available in Zenodo (see Data availability).

To further characterize the differences between standard and deviant responses across the auditory pathway, we employed the common stimulus-specific adaptation index (CSI). This index is defined as the sum of the differences between the deviant and standard responses for the two trains, normalized by the sum of both the deviant and standard responses for both trains (see Methods). The analysis showed that the distribution of the CSI shifted positively (Fig 5D), indicating a greater distinction between deviant and standard responses as auditory processing progresses from the IC to A1. The average CSI increased significantly from the IC to A1 (*p* < 0.001 for both comparisons: A1 versus MGB and MGB versus IC, ANOVA with post-hoc test, Fig 5E).

Consistent sensitivity to deviant sounds was observed across different sound pairs in the population. For RN_60_ and Con, differences between deviant and standard responses were minimal in the IC (RN_60_, *p* = 0.31; Con, *p* = 0.90; independent**-samples** *t* test, Fig 5H) but became significantly pronounced in A1 (RN_60_, *p* < 0.001; Con, *p* < 0.001; independent**-samples** *t* test, Fig 5F). The CSI distribution similarly shifted positively (Fig 5I), with the average CSI showing a significant increase from IC to A1 (*p* < 0.001 for both comparisons: A1 versus MGB and MGB versus IC, ANOVA with post-hoc test, Fig 5J). For Asc and TW_30_, differences between deviant and standard responses were minimal in the IC (Asc, *p* = 0.71; TW_30_, *p* = 0.42; independent**-samples** *t* test, Fig 5M) but became significantly pronounced in A1 (Asc, *p* = 0.035; TW_30_, *p* < 0.001; independent**-samples** *t* test, Fig 5K). The CSI distribu*t*ion similarly shifted positively (Fig 5N), with the average CSI showing a significant increase from IC to A1 (*p* < 0.001 for both comparisons: A1 versus MGB and MGB versus IC, ANOVA with post-hoc test, Fig 5O). CSI distributions by subdivision for MGV, MGD, CIC, and EIC across the three click-train stimulus pairs are shown in S2 Fig.

The relatively lower CSI observed for click trains in the IC could, in principle, reflect the fact that IC neurons often show weaker SSA than neurons in the MGB and A1 [13,17,22,24]. To examine whether a general difference in SSA expression across auditory regions could account for the reduced click-train CSI, we analyzed a subset of neurons that showed significantly positive CSI values for a pair of complex tones (CT; see Methods, Session 5: Complex Tone Oddball Procedure) in the oddball paradigm, including neurons from A1 (*n* = 94), MGB (*n* = 109), and IC (*n* = 140). This selection provided a population of neurons that exhibited SSA for an independent stimulus class, allowing us to test whether reduced click-train CSI persisted among neurons capable of expressing SSA. Neuronal responses and corresponding CSI values for these neurons are shown in S3 Fig.

In this dataset, complex-tone CSI was significantly higher in the IC than in A1 (*p* < 0.001, Wilcoxon rank-sum test; S3B–S3C Fig). To reduce possible selection bias, we further performed a split-half cross-validation analysis, in which neurons were selected using one subset of the CT trials based on significantly positive CSI, and CSI was then recomputed for these neurons using an independent held-out subset of trials. The selected neurons continued to show positive CT-evoked SSA in the held-out data, indicating that this control effect was not attributable solely to circular selection or noise. However, when the same neuronal subset was examined using the three pairs of click-train stimuli, CSI still tended to increase from IC to A1 (S3E, S3H, and S3K Fig) and remained significantly higher in A1 than in IC for all three pairs (*p* < 0.001, ANOVA with post hoc t-tests; S3F, S3I, and S3L Fig). Thus, the lower click-train CSI in the IC was not explained solely by a general difference in SSA expression across auditory regions. However, this analysis does not exclude the existence of an IC subpopulation with particularly large CSI for specific click-train pairs, because neurons were selected based on complex-tone CSI. Accordingly, we interpret the higher click-train CSI in A1 as evidence of stronger context-dependent sensitivity to the tested click-train patterns in A1 than in IC.

To further examine whether neural sensitivity to click-train temporal patterns could be observed when long-term spectral magnitude was matched, we performed an additional oddball experiment using two pairs of click trains with identical frequency-domain magnitude spectra but different time-domain structures (S4 Fig). The corresponding cochleagram representations of these matched stimuli are shown in S1 Fig. FFT spectra overlapped within each stimulus pair, confirming matched long-term spectral magnitude between the two sounds (S4A and S4E Fig). Despite this spectral matching, population responses still showed deviant–standard differences, particularly in A1 and MGB (S4B and S4F Fig). CSI distributions were shifted positively in A1 and, to a lesser extent, in MGB, whereas IC showed weaker effects (S4C and S4G Fig). Across auditory levels, CSI was significantly higher in A1 than in IC for both spectrally matched stimulus pairs (S4D and S4H Fig). These results indicate that differences in long-term spectral magnitude are not required for the observed context-dependent responses. However, because the paired stimuli differed in their short-term spectrotemporal organization, this control does not isolate a purely temporal component.

Since MGB and AC are extensively connected [25], the sensitivity to temporal configuration in MGB may arise from AC top-down inputs. To explore this possibility, we used cooling techniques to decrease neuronal activities in the auditory cortex (Fig 6) and thus decrease the top-down inputs to MGB. In the normal condition before cooling the AC, the MGB neuronal population (*n* = 86) demonstrated robust SSA for the three pairs, and the deviant responses were stronger than the standard responses (RN_10_, *p* = 0.18; TW_50_, *p* < 0.001, top row in Fig 6A; RN_60_, *p* < 0.001; Con, *p* = 0.40, top row in Fig 6D; Asc, *p* < 0.05; TW_30_, *p* < 0.001, top row in Fig 6G; independent-samples *t* test) with average CSIs of 0.12, 0.13, and 0.089 respectively (top row in Fig 6B, 6E and 6H). During the cooling of AC, the same MGB population demonstrated comparable responses to deviant and standard stimuli for all three pairs (RN_10_, *p* = 0.86; TW_50_, *p* = 0.59, bottom row in Fig 6A; RN_60_, *p* = 0.29; Con, *p* = 0.99, bottom row in Fig 6D; Asc, *p* = 0.98; TW_30_, *p* = 0.50, bottom row in Fig 6G; independent-samples *t* test) with average CSIs of 0.038, 0.079, and 0.071 respectively (bottom row in Fig 6B, 6E, and 6H). Three sessions of experiments were carried out involving three rats. The CSI of the cooling condition was significantly smaller than the normal pre-cooling condition for the pair of RN_10_ and TW_50_ (*p* < 0.001, Fig 6C, paired *t* test), and the pair of RN_60_ and Con (*p* = 0.0119, Fig 6F, paired *t* test). However, for the pair of Asc and TW_30_, the CSIs were similar between the cooling condition and the normal pre-cooling condition (*p* = 0.095, Fig 6I, paired *t* test), mainly due to the fact that the CSI is 0.089 in the normal condition, which is already very small. As a control experiment, we verified that AC cooling effectively suppressed local cortical activity (S5A Fig) without significantly altering the SSA of MGB neurons in response to complex tones (*p* = 0.53, paired *t* test; S5B–S5D Fig). Thus, cortical feedback contributes importantly to MGB sensitivity to these click-train temporal patterns, but the present data do not determine the specific circuit mechanism underlying this effect.

**Fig 6:**
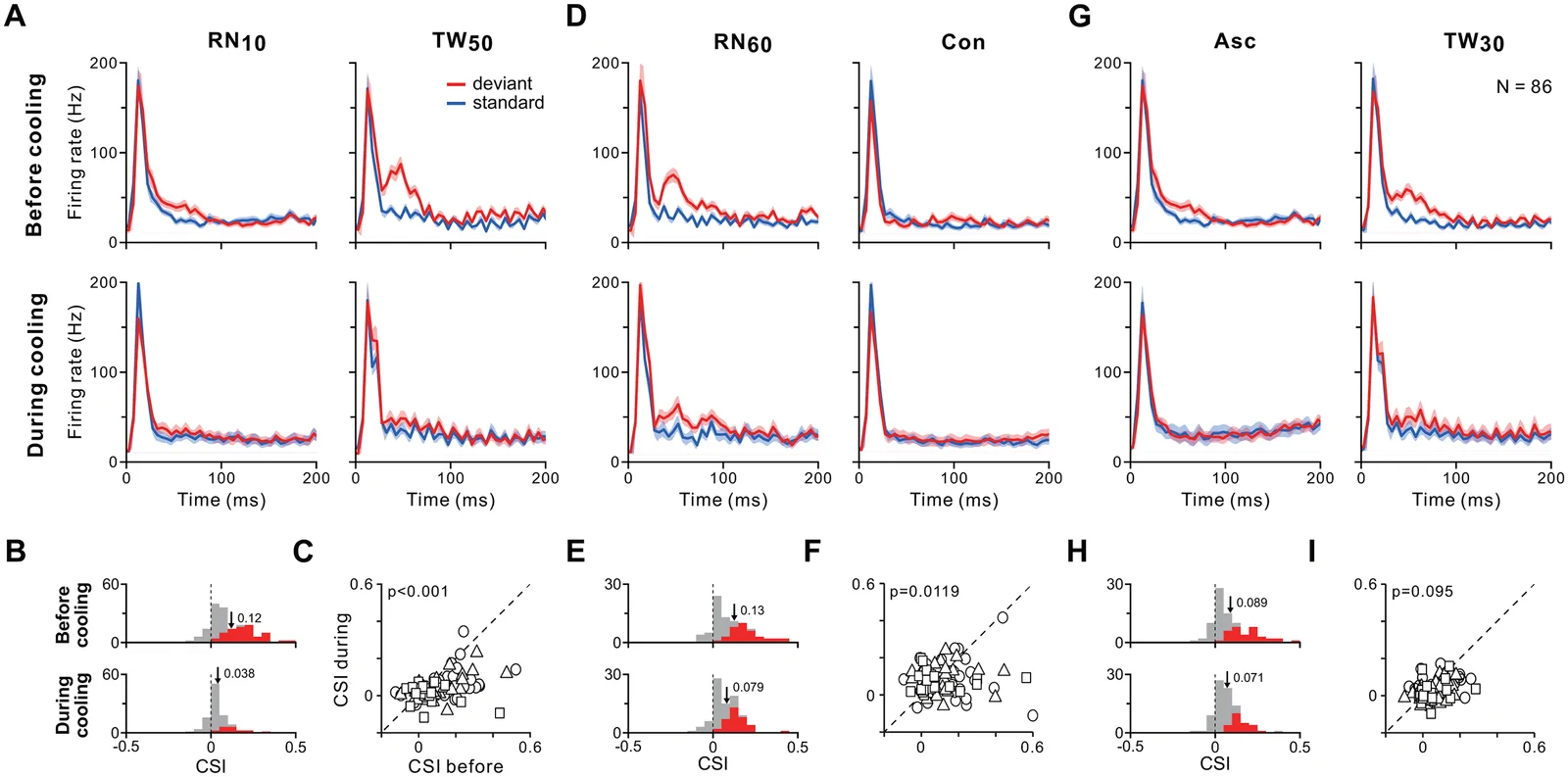
Effect of AC cooling on the response to oddball paradigm with temporally merged click-train in MGB. **(A)** Populational PSTHs displaying responses to RN_10_ (left) and TW_50_ (right) as deviant (red) and standard (blue) stimuli under two conditions: before AC cooling (top row), and during AC cooling (bottom row). **(B)** Distribution of CSI before cooling (top row) and during cooling (bottom row), with arrows indicating the means. Red bars highlight neurons with CSI significantly greater than 0, determined by a nonparametric bootstrap procedure with 1,000 iterations (*p* < 0.05). **(C)** Comparison of CSI between before and during AC cooling with different symbols representing data from different rats (*N* = 3 rats, total neurons = 86). *p* < 0.001, paired *t* test. **(D–F)** Population responses from the same dataset to the sound pair in the oddball paradigm (RN_60_, Con), following the same conventions as panels (A–C). *p* = 0.0119, paired *t* test. **(G–I)** Population responses to sound pair in the oddball paradigm (Asc, TW_30_), adhering to the same conventions as panels (A–C). *p* = 0.095, paired *t* test. Source data are available in Zenodo (see Data availability).

Since AC contributed importantly to sensitivity to click-train temporal patterns, we next asked whether this sensitivity differed across cortical layers. To support laminar assignment, Neuropixels probe tracks were reconstructed histologically, and layer boundaries were further estimated from noise-evoked LFP and current source density (CSD) profiles (S6 Fig). Based on recording depth and CSD profiles, A1 neurons were grouped into supragranular layers (layers I/II, *n* = 30), middle layers (layers III/IV, *n* = 176), and deep layers (layers V/VI, *n* = 465) for analysis. For the pair RN_10_ and TW_50_, the average CSI values were 0.162, 0.166, and 0.099 in the supragranular, middle, and deep layers, respectively (Fig 7A). For the pair RN_60_ and Con, the average CSI values were 0.145, 0.136, and 0.08 (Fig 7C), and for the pair Asc and TW_30_, the average CSI values were 0.106, 0.090, and 0.052 (Fig 7E), respectively. When analyzing complex tones, the average CSI values were 0.073, 0.11, and 0.053 (Fig 7G) across the supra, middle, and deep layers. Interestingly, the CSI in the supra layer was significantly greater than in the deep layer (*p* < 0.001, ANOVA with post-hoc test) for all three pairs of click trains, but similar across layers for complex tones (*p* = 0.45, ANOVA with post-hoc test). These findings suggest that superficial cortical layers play a particularly important role in encoding temporal configuration, whereas layer-specific differences are less marked for frequency-related features.

**Fig 7:**
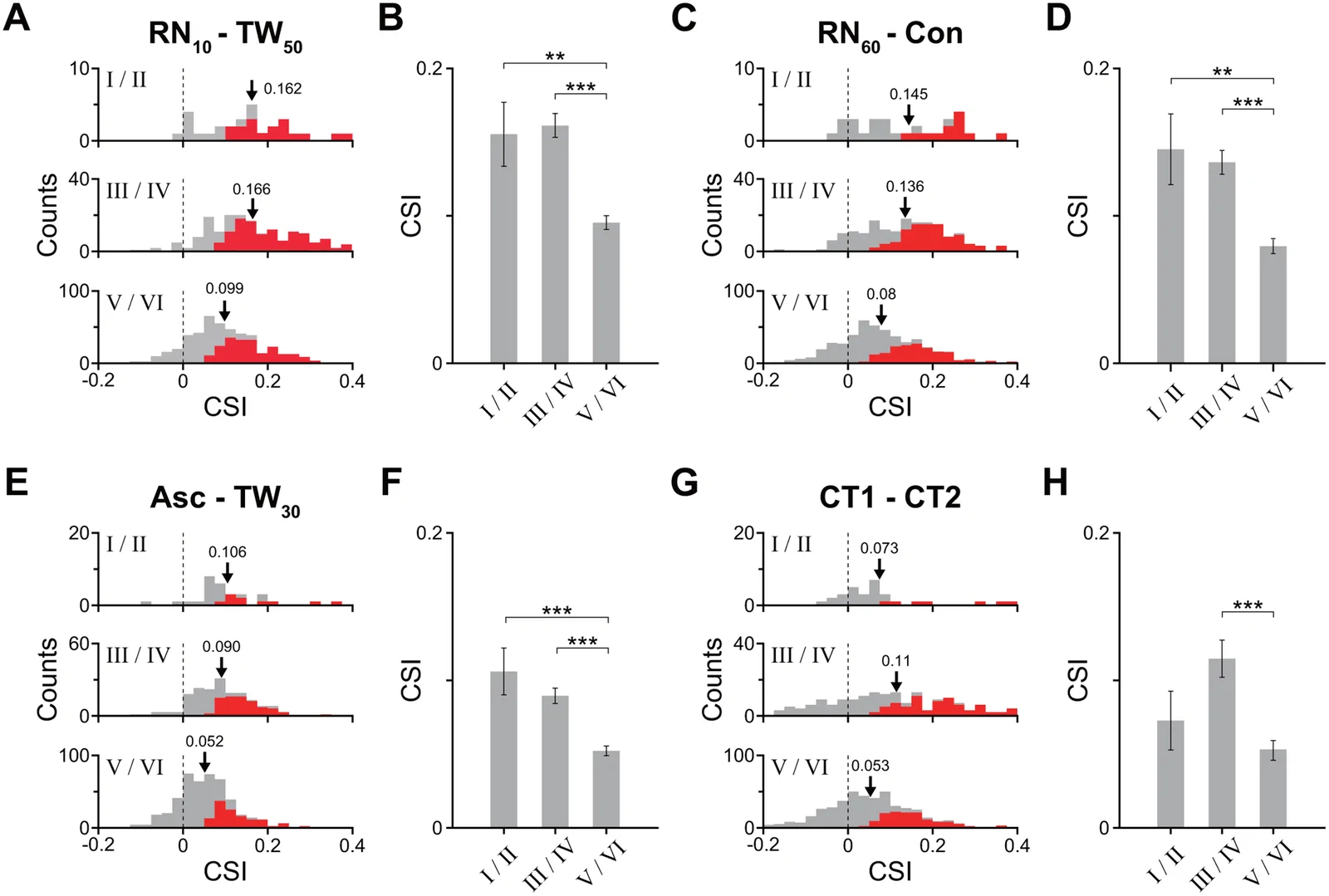
Neuronal responses to temporally merged click-train and complex tones across cortical layers in A1 in the oddball paradigm. **(A)** Distribution of CSI in the oddball paradigm (RN_10_, TW_50_) across cortical layers: supra layer (layers Ⅰ/Ⅱ, *n* = 30, top row), middle layer (layers Ⅲ/Ⅳ, *n* = 176, middle row), and deep layer (layers Ⅴ/Ⅵ, *n* = 465, bottom row). Arrows point to the mean. **(B)** Comparison of CSI among supra, middle, and deep layers. Red bars highlight neurons with CSI significantly greater than 0, determined by a nonparametric bootstrap procedure with 1,000 iterations (*p* < 0.05). Error bars represent standard error; n.s., no significant difference; ** *p* < 0.01; *** *p* < 0.001, ANOVA with post-hoc test. **(C–H)** Population responses from the same datasets featured in panels (A**–**B) to two additional click-train pairs and a complex-tone pair: (C-D) RN_60_ and Con, (E-F): Asc and TW_30_, and complex tones pair (G-H). Following the same conventions as panels (A-C). Source data are available in Zenodo (see Data availability).

## Discussion

In this study, we show that click trains sharing the same mean inter-click interval but differing in temporal pattern can evoke different behavioral and neural responses across multiple levels of the auditory system. In humans, listeners reliably discriminated several click-train patterns, indicating perceptual sensitivity to millisecond-scale temporal patterning (Fig 1). Human EEG revealed deviance-related cortical response differences associated with changes in click-train pattern (Fig 2). In awake rats, ECoG recordings also showed context-dependent cortical response differences across click-train conditions (Fig 3). Along the IC–MGB–A1 hierarchy, extracellular recordings showed weaker oddball/context sensitivity in IC, intermediate sensitivity in MGB, and stronger stimulus-specific adaptation in A1 (Figs 5 and S3–S4). Reversible cooling of auditory cortex reduced MGB sensitivity to these click-train patterns (Fig 6), indicating an important contribution of corticothalamic feedback. Layer analyses further showed stronger sensitivity in supragranular than infragranular layers of A1 in the present recordings (Fig 7). These findings indicate that distinct click-train temporal patterns, together with their associated spectrotemporal consequences, are differentially represented across the auditory system, with stronger sensitivity emerging in higher auditory structures.

### Click-train temporal patterns and their auditory representation

Rapid click trains have been studied extensively in relation to temporal coding, repetition-rate coding, and pitch perception [1,2,8,26]. They provide a useful approach for systematically manipulating millisecond-scale temporal patterns, although such manipulations also produce associated short-term spectrotemporal differences. At ICIs below 30 ms, regular click trains are generally perceived as a single pitch-like sound [1], consistent with temporal integration. Different periodic and irregular click trains can nevertheless evoke distinguishable perceptual qualities and neural responses. The present study builds on this literature by examining behavioral discrimination and oddball/context sensitivity to selected click-train patterns across successive levels of the auditory pathway.

In a delayed match-to-sample task, participants reliably discriminated click trains that differed in temporal pattern while sharing the same mean inter-click interval and overall duration, with performance showing a graded pattern across stimulus pairs (Fig 1). The behavioral discriminability of these click-train patterns must ultimately arise from differences in the auditory activity evoked by the sounds. The present behavioral results therefore show that the tested click-train pairs generated neural differences that were available to perceptual decision-making in the delayed match-to-sample task. However, these results do not by themselves identify the exact acoustic or temporal statistics underlying that sensitivity. Moreover, oddball paradigms using pairs such as RN_10_–Con, RN_10_–TW_30_, Asc–TW_30_, TW_10_–TW_30_, TW_50_–TW_30_, and RN_40_–TW_30_ elicited clear MMN responses (Fig 2E–2H), indicating automatic cortical sensitivity to changes in these patterns even without an explicit task. Thus, human behavioral and EEG results reveal sensitivity to click-train pattern differences, while the acoustic descriptors and computations that support this sensitivity remain unresolved.

A key open question is which acoustic or temporal statistics contribute to the observed behavioral discrimination. Although the tested click-train pairs shared the same mean inter-click interval, the present study does not identify the specific computational variable extracted by the auditory system. Several candidate accounts remain possible, including temporal regularity-related cues, pitch-like perceptual consequences of fast click trains, modulation-based representations, asymmetric temporal filtering/integration, or other higher-order temporal descriptors. Because the current stimulus set was not designed to isolate these alternatives parametrically, the present data do not allow a decisive distinction among them. A related issue concerns the relationship between behavioral and neural sensitivity. Some stimulus pairs that were weakly discriminable in the delayed match-to-sample task, such as TW_10_ versus TW_30_ in Fig 1C, nevertheless elicited measurable EEG differences in the oddball paradigm, such as TW_10_ versus TW_30_ in Fig 2G. This should not be interpreted as a strict dissociation between perception and neural representation. Behavioral performance depends on additional factors, including task demands, sensory memory, attention, decision criteria, and response mapping. Neural responses can therefore contain information that is at least as fine-grained as, and sometimes finer than, what is expressed in overt behavior. We therefore interpret the EEG results as evidence for context-dependent neural sensitivity to click-train pattern differences, rather than as evidence for a dissociation between neural and behavioral discrimination. This interpretation is consistent with previous work showing that fast click trains can generate pronounced pitch-related and spectrotemporal effects [4,27], and that closely spaced clicks may interact monaurally and spectrally in related perceptual paradigms [28,29].

The additional control using time-reversed stimulus pairs matched in long-term FFT magnitude produced oddball/context-dependent differences in neuronal responses, with stronger CSI in A1 than in IC (S4 Fig). Because time reversal preserves the long-term FFT magnitude spectrum while altering the time-domain waveform and short-term spectrotemporal structure, this control indicates that differences in long-term spectral magnitude are not required for the observed context sensitivity. However, it does not isolate a purely temporal component of the neural response or exclude feedforward mechanisms sensitive to short-term spectrotemporal differences. Harpaz and colleagues [30] similarly demonstrated stimulus-specific adaptation to sounds with matched long-term spectra and observed stronger effects in A1 than in IC and MGB. Their findings, like ours, argue against explanations based solely on adaptation within fixed spectral channels while remaining compatible with mechanisms sensitive to short-term spectrotemporal structure. Thus, the present findings are consistent with these previous observations and extend them to the rapid click-train stimuli examined here.

### Neuronal sensitivity to click-train temporal patterns along the auditory pathway

We examined neuronal sensitivity to click-train temporal patterns using SSA within an oddball paradigm, assessing responses across multiple levels of the auditory pathway, from IC to A1 (Fig 4A). SSA has been extensively studied in the auditory cortex [20,24,31–38], medial geniculate body [33,39–44], inferior colliculus [21,45–49], and cochlear nucleus [19,50]. Typically, these studies have utilized pure tones to generate oddball stimuli, with previous data suggesting that SSA is mediated by adaptation in narrowly tuned inputs, a concept supported by computational modeling [51] and the discovery of synaptic SSA [15]. However, recent studies propose that SSA is predominantly a predictive process [22,30,52–54]. Our click-train paradigm extends this framework by testing oddball/context sensitivity for sounds constructed from identical pulses with the same average ICI but different temporal patterns and associated spectrotemporal consequences. Under such conditions, simple feedforward adaptation would be expected to produce similar adaptation. Consistent with this, we observed minimal SSA in IC and only modest SSA in MGB, while A1 exhibited pronounced SSA for click-train temporal pattern (Fig 5). This hierarchical pattern is compatible with predictive-processing accounts, but does not by itself distinguish predictive signaling from increasingly complex feedforward or recurrent adaptation.

The weaker click-train SSA observed in IC is unlikely to reflect a general inability of IC neurons to show SSA [17,22,55], because IC neurons selected for positive CSI to complex-tone stimuli showed reliable SSA under that control condition, including in split-half cross-validation (S3 Fig). This analysis nevertheless does not exclude stimulus dependence, population heterogeneity, or IC subpopulations sensitive to particular click-train pairs. At the same time, the majority of neurons showed deviant > standard effects, as reflected by the overall positive shift of the CSI distributions, indicating that the dominant population tendency remained consistent with deviance-related sensitivity to click-train temporal patterns under oddball context. However, a subset of neurons and response windows showed standard > deviant effects, suggesting that the observed oddball-related responses are not exclusively stimulus-specific and may also include contributions from broader contextual or adaptation-related processes.

An alternative interpretation is that SSA is more broadly expressed in cortex than in subcortical structures, such that the stronger cortical effects observed here may reflect a more general expansion of adaptation/context sensitivity across a wider range of sounds, rather than a mechanism uniquely specialized for the click-train patterns used in this study. The present data do not distinguish decisively between these possibilities, and the cortical findings should therefore be interpreted with appropriate caution.

The present findings build on previous studies of hierarchical stimulus-specific adaptation, including the work of Parras and colleagues, who demonstrated increasing SSA along the IC–MGB–A1 pathway using frequency oddballs [22]. Work with “tone clouds” provides an important comparison. Harpaz and colleagues (2021) used spectrally balanced tone clouds—each composed of the same set of tones presented once—and found early and strong SSA in A1 but only delayed and weak SSA in IC and MGB [30], suggesting that A1 encodes abstract temporal–spectral patterns. Tone clouds can be conceptualized as comprising a spectral configuration in the frequency domain and a temporal configuration in the time domain. Recent studies have shown that auditory cortical neurons can exhibit bursting responses selective for particular tone configurations rather than individual tones [56,57]. In contrast to these tone-based paradigms, our click-train stimuli used identical pulses and differed primarily in temporal pattern, while also giving rise to associated short-term spectrotemporal consequences. Under these conditions, A1 neurons showed stronger SSA than IC neurons (Figs 5 and S3), consistent with stronger cortical sensitivity to click-train temporal patterns. Thus, our findings extend this established line of research from frequency oddballs and configural tone clouds to rapid click-train patterns. However, because these stimuli retain spectrotemporal differences, and because matching the long-term FFT magnitude does not ensure equivalent peripheral or neurogram-level representations, the present findings should not be interpreted as isolating a purely temporal mechanism.

Our cooling experiments further support this view. Reversible suppression of auditory cortex substantially reduced temporal pattern sensitivity in MGB (Fig 6), indicating that thalamic SSA for click-train temporal pattern depends in large part on corticothalamic feedback. This is consistent with extensive reciprocal connectivity between MGB and auditory cortex [58] and supports a model in which corticothalamic feedback shapes context-dependent thalamic responses to click-train temporal patterns. However, compared with previous studies of corticofugal modulation of MGB responses [58], differences in stimulus design, analysis windows, and recording conditions may influence the observed corticofugal effects.

Finally, layer-specific analysis revealed that supragranular layers of A1 exhibit stronger SSA for temporal pattern than infragranular layers, whereas SSA for complex tones did not show such pronounced laminar differences (Fig 7). This suggests that superficial layers—known to support horizontal integration and cortico–cortical communication—play a privileged role in encoding temporal pattern and in propagating prediction-error signals within the cortical hierarchy.

In summary, our findings show that sensitivity to click-train temporal patterns increases along the auditory hierarchy, emerging more strongly in higher structures such as MGB and A1 than in IC. Rather than identifying a single site or mechanism for temporal-pattern encoding, the present results suggest a hierarchical and feedback-influenced organization in which cortical and corticothalamic circuits contribute to context-dependent responses to temporally patterned click trains. Future work should identify the specific synaptic and circuit mechanisms—such as recurrent connectivity, inhibitory microcircuits, and corticothalamic loops—that implement this sensitivity and link these mechanisms to perception and behavior. In particular, relating neuronal sensitivity to explicit perceptual thresholds, behavioral biases, and formal stimulus descriptors will be necessary to establish how click-train temporal patterns are transformed into perceptual judgments.

### Limitations of the present study

Several important limitations of the present study should be acknowledged. First, although our results show that different click-train temporal patterns can drive reliable behavioral and neural differences, the present dataset does not identify which specific temporal or acoustic statistics are encoded or compared by the auditory system. The stimulus sets used here were not designed to isolate a single descriptor, such as temporal regularity, monotonicity, transition probability, entropy, higher-order temporal correlations, pitch-related cues, or modulation-based representations, while holding others constant. Thus, the current findings demonstrate sensitivity to click-train temporal patterns, but do not yet establish the precise computational variables underlying that sensitivity. Second, the underlying neuronal mechanisms remain incompletely resolved. The principal neuronal analyses rely on CSI/SSA measures, which quantify differences between responses to the same stimulus presented as a deviant or as a standard. However, these measures do not by themselves distinguish deviance-specific processing or prediction-error signaling from use-dependent adaptation, including differential adaptation of stimulus-selective feedforward inputs. Resolving these alternatives would require an explicit model of adaptation dynamics and testing whether deviant responses exceed model predictions. Similarly, although cortical cooling provided causal evidence that cortical input contributes to MGB sensitivity under our paradigm, the present data do not determine whether this effect reflects predictive signaling, gain control, adaptation dynamics, changes in inhibitory–excitatory balance, or other forms of context-dependent modulation. Accordingly, the present results characterize context-dependent sensitivity to the tested click-train patterns across the auditory pathway without specifying the underlying neuronal mechanism. Third, cross-species comparisons must be interpreted with particular caution. While humans and rats showed analogous neural sensitivity to the same stimulus classes, we did not behaviorally validate in rats that these click trains occupy the same perceptual regime as in humans, and therefore the present study does not establish perceptual equivalence across species. More broadly, differences between behavioral performance and neural response measures indicate that neural sensitivity to temporal-pattern differences may exceed what is expressed under a particular behavioral task, and the current study does not provide a formal model linking perceptual discrimination, population coding, and deviance-related responses across levels of the auditory pathway. Addressing these issues will require future work using more controlled parametric stimulus designs, formal model comparison, direct neural decoding analyses, and more specific circuit manipulations.

## Methods

### Ethics statement

For the human experiments, the study protocol was conducted in accordance with applicable ethical standards, including the principles expressed in the Declaration of Helsinki, and was approved by the Institutional Review Board (IRB-20230131-R). Participants were informed about the experimental procedures and study details before providing consent. Verbal informed consent was obtained from all participants before participation in the study. After completing the experiment, participants received monetary compensation for their participation.

For the rat experiment, the study protocol was approved by the Animal Subjects Ethics Committee of Zhejiang University (ZJU20210078).

### Stimuli

We developed three experimental procedures for EEG, ECoG, and extracellular recording, employing 10 unique click trains as depicted in supplementary information S1 Table. Each click train lasted 200 ms, consisting of 51 fixed pulses, with each pulse lasting 200 µs and an average ICI of 4 ms. The sound intensity for all click trains was uniformly calibrated to 60 dB SPL to ensure consistency across experiments involving both human participants and animal subjects. Calibration was performed using a ¼-inch condenser microphone (Brüel and Kjær 4954, Nærum, Denmark) and a PHOTON/RT analyzer (Brüel and Kjær, Nærum, Denmark).

To further characterize the acoustic representations of the stimuli, we additionally generated two pairs of spectrally matched click trains, in which the temporal configurations differed while the long-term spectral properties were matched. The original click trains and these additional matched pairs were analyzed using a model-based peripheral auditory representation. Specifically, cochleagrams were generated using an ERB-spaced gammatone filterbank implemented in the Auditory Modeling Toolbox (AMT) [59]. The filterbank consisted of 96 auditory filters with center frequencies ranging from 80 Hz to 40 kHz. The envelope outputs of each auditory filter were converted to logarithmic amplitude values (dB) for visualization. The corresponding audio files are provided in S1 Audio, and the cochleagram representations generated from these stimulus waveforms are shown in S1 Fig.

### Participants and surgical procedures

#### EEG experiment participants.

We recruited 32 volunteers (18 males and 14 females; mean age = 25.3 years, SD = 3.59) with normal hearing to participate in the EEG experiments. Of these, 24 participated in the Active Listening Session, and all 32 took part in the Passive Listening Sessions.

#### ECoG surgery.

Three adult male Wistar rats (280–340 g, 9–12 weeks), each with clean external ears, were selected for this study. We implemented sterile surgical techniques for the implantation of the headpost and the ECoG array. Anesthesia was administered using pentobarbital sodium (40 mg/kg) along with atropine sulfate (0.05 mg/kg, s.c.) given 15 min before the procedure to reduce tracheal secretion. Xylocaine (2%) was applied liberally to the incision site to minimize pain. A head fixation bar was secured to the top of the skull using dental cement and six titanium screws. A craniotomy exposed a 4.5 mm × 5 mm area above the auditory cortex on the left side, leaving the dura mater intact. The ECoG array (KD-PIFE, KedouBC, China), consisting of 32 electrodes in a 6 × 6 grid with 1 mm spacing, was implanted. The array covered an approximate area of 5 mm × 5 mm, encompassing the entire auditory cortex (AC) (Fig 3B). After the implantation, the ECoG array was covered with artificial brain gel, followed by the repositioning of the bone flap over the surgical site. The area was then sealed with dental cement to ensure closure and protection. Postoperative recovery lasted 7 days, during which anti-edema medications and antibiotics were administered, and the animals’ weight was monitored daily to ensure their well-being.

#### Extracellular recording surgery.

Three adult male Wistar rats (280–340 g, 9–12 weeks) were utilized for extracellular electrode recordings, undergoing a similar procedure to the ECoG surgery for headpost stabilization. Then, the left lateral and dorsal surfaces of the skull were exposed post-surgery. Once cleaned and dried, reference landmarks were established at the bregma and on the left lateral skull for precise electrode positioning. Target areas for the electrode recordings included the left primary auditory cortex, the left medial geniculate body, and the left inferior colliculus (IC). To prevent tissue overgrowth, the skull above these areas was thinly coated with dental cement and enclosed with a protective wall. A handheld micro-drill created a small opening (approximately 2 mm × 2 mm) at the designated target site, which was subsequently sealed with brain gel. On the recording day, the dura mater was carefully punctured, and the electrode was vertically inserted into the target area using the established landmarks for guidance.

#### Cooling AC.

To investigate the functional role of AC in processing click-train temporal configurations, we employed a reversible cooling technique to deactivate the AC and recorded activity from the ipsilateral medial geniculate body (MGB). A 4-mm diameter cryoloop was positioned over the AC, manually bent to fit the cortical surface and covering both the primary and nonprimary fields. Cooling was achieved by circulating anhydrous ethanol, cooled by dry ice, through the cryoloop using a peristaltic pump. A temperature probe was placed near the AC to continuously monitor the temperature, which was regulated by adjusting the ethanol flow rate, maintaining a stable temperature of 8 ± 3 °C. This cooling temperature effectively deactivates all layers of the AC, including corticofugal projections from layers V and VI, without significantly affecting neural activity in subcortical areas such as the MGB [58]. MGB neuronal activity was recorded 5 min after cooling began. Additionally, we monitored AC neuron activity before, during, and after cooling to confirm the deactivation effect and ensure that the structural and functional integrity of the deactivated area remained intact.

### Experimental procedures

For human EEG recordings, click sounds were synthesized using MATLAB R2022b (The MathWorks, Natick, MA, USA) on a computer equipped with a high-fidelity sound card (Creative, AE-7). These clicks were produced with a sampling rate of 384 kHz and 32-bit precision and then broadcast through a stereo speaker system (Golden Field M23), ensuring the precise presentation of click train sequences. In contrast, animal ECoG and Extracellular recording experiments were conducted within a sound-proof room, with stimuli played contralaterally to the recording site in rats. These acoustic stimuli were digitally generated using a computer-controlled Auditory Workstation (RZ6, TDT) at a sampling rate of 97656 Hz and delivered through magnetic speakers (MF1, TDT) within the TDT systems.

#### EEG experimental procedures.

The study comprised five experimental sessions, including one active discrimination session and four passive listening sessions. EEG signals were recorded using a 64-channel system.

*Active Listening Session (*Fig 1B*).* In this session, participants engaged in a delayed match-to-sample task. An initial click train (one of the 10 click trains listed in S1 Table) was played, followed by a 500-ms silence before presenting a second click train, randomly selected from the same pool of 10 options. Participants were required to assess whether the two sounds were identical. Responses were recorded using the left arrow key for differing sounds and the right arrow key for identical sounds. We selected 20 sound pairs, as shown in Fig 1C, each presented 40 times per session. Among these 20 pairs, 7 were identical pairs and 13 were different-stimulus trials. Therefore, identical and different-stimulus trials accounted for 35% and 65% of all trials, respectively.

To quantify discrimination sensitivity while accounting for response bias, we calculated d′ for each stimulus pair using signal detection theory. For each different-stimulus pair, the hit rate was defined as the proportion of trials in which participants responded “different” to that pair. The false-alarm rate was defined as the proportion of trials in which participants responded “different” to identical-pair trials. Because multiple identical-pair conditions were included, the false-alarm rate was estimated using responses pooled across all identical-pair trials. For each participant, a pooled false-alarm rate was calculated across all seven identical-pair conditions. This participant-specific pooled false-alarm rate was then used to calculate $d^{\prime }$for each different-pair condition. To avoid infinite d′ values, hit and false-alarm rates of 0 or 1 were corrected using the standard log-linear correction. Sensitivity was then calculated as $d^{\prime }=Z(H)-Z(FA)$. A global d′ value was also calculated by pooling responses across all different-stimulus trials and all identical-stimulus trials.

*Passive Listening Sessions 2–5.* Following the active discrimination session, four passive listening sessions were conducted. During these sessions, participants were exposed to auditory stimuli without performing any behavioral tasks. Each session consisted of a continuous sequence of auditory stimuli, with each sound lasting 200 ms and presented consecutively with a 300-ms interval between the onset of consecutive sounds, totaling 2,400 presentations. The stimuli included seven click trains randomly selected from a pool of 10 different click trains. One click train was presented most frequently (2,160 times) and served as the standard stimulus, while the other six types were used as deviant stimuli, each presented 40 times and interspersed among the standard sounds. The configuration ensured that each deviant stimulus was preceded by 7–11 standard stimuli, preventing consecutive presentations of deviant stimuli.

- Session 2: Standard stimulus was ‘Con’; deviant stimuli included ‘Asc’, ‘RN_10_’, ‘RN_40_’, ‘RN_60_’, ‘TW_30_’, and ‘TW_50_’.
- Session 3 (Fig 2A): Standard stimulus was ‘TW_30_’; deviant stimuli included ‘Asc’, ‘RN_10_’, ‘RN_40_’, ‘RN_60_’, ‘TW_10_’, and ‘TW_50_’.
- Session 4: Standard stimulus was ‘RN_60_′; deviant stimuli included ’Con’, ‘RN_10_’, ‘TW_10_’, ‘TW_30_’, ‘TW_50_’, and ‘TW_70_’.
- Session 5: Standard stimulus was ‘RN_10_’; deviant stimuli included ‘Con’, ‘Asc’, ‘RN_40_’, ‘TW_10_’, ‘TW_30_’, and ‘TW_50_’.

Participants were instructed to maintain a stationary head position throughout the experiment and were allowed a 5-min break after each session to prevent fatigue and ensure attentiveness.

#### ECoG experimental procedures.

The study included three sessions specifically designed for ECoG recordings. In each session, we selected two click trains from the previously described set of 10 click trains to form a stimulus pair, which was used to create classic oddball sequences (Fig 3A). Each oddball sequence consisted of 300 stimulus presentations with an inter-stimulus interval (ISI) of 300 ms. Within each sequence, one click train served as the standard stimulus, presented 270 times (90% of trials), and the other served as the deviant stimulus, presented 30 times (10% of trials). The roles of the two stimuli were reversed in a second sequence, so each click train was presented once as a standard and once as a deviant. The only difference across Sessions 1, 2, and 3 was the choice of stimulus pairs, while all other experimental parameters were identical. Specifically, for *Session 1*, the stimulus pair comprised ‘RN_10_’ and ‘TW_50_’; for *Session 2*, the stimulus pair comprised ‘RN_60_’ and ‘Con’; whereas for *Session 3*, it consisted of ‘Asc’ and ‘TW_30_’.

#### Extracellular recording experimental procedures.

The study included six sessions specifically designed for extracellular recordings.

*Session 1 Frequency screening procedure*. A sequence of tones was presented randomly from a speaker positioned contralateral to the recording site. Each tone lasted 100 ms with a rise-fall time of 5 ms. The frequency range spanned from 0.5 kHz to 48 kHz, divided into 26 logarithmic steps. The intensity of these tones varied from 0 to 70 dB SPL in increments of 10 dB. Each combination of frequency and intensity was repeated five times with a 300-ms interval between stimuli. This session aimed to measure basic neuronal response properties, specifically the frequency response area (FRA) and characteristic frequency (CF).

*Session 2–4 Oddball procedure*. These sessions employed the same oddball paradigm as described for the ECoG experiments, with the only difference being a shorter ISI of 250 ms. Stimulus pairs were selected from the previously described set of 10 click trains. Each oddball sequence consisted of 300 presentations, with 270 standards (90%) and 30 deviants (10%). Each stimulus in the pair was presented once as a standard and once as a deviant. The stimulus pairs used were: *Session 2 -* ‘RN_10_’ and ‘TW_50_’; *Session 3* - ‘RN_60_’ and ‘Con’; and *Session 4* - ‘Asc’ and ‘TW_30_’.

*Session 5 Complex Tone Oddball procedure*. To complement the findings obtained from click train pairs, we designed an oddball sequence using two logarithmically spaced tone complexes. One tone complex comprised frequency components spaced at 1/8-octave intervals, beginning at 600 Hz. The component frequencies (e.g., 654, 712, 777, …, 39,910 Hz) followed this logarithmic progression. The other tone complex began at 680 Hz, also with 1/8-octave spacing between adjacent components. Thus, the two complex tones did not share similar frequencies. Except for the frequency components, other stimulus parameters for this oddball procedure were identical to those employed for the click trains.

*Session 6 Spectrally matched click-train oddball procedure.* To further test whether click-train temporal organization could affect neural responses when long-term spectral magnitude was matched, we generated two pairs of click trains with identical FFT magnitude spectra but different time-domain structures (S4 Fig). Within each pair, the two stimuli had the same spectral magnitude and overall power but differed in temporal waveform. These stimuli were presented in the same two-sequence oddball design used for *Sessions 2–4*, with each stimulus serving once as the standard and once as the deviant. All other stimulus parameters and analysis procedures were identical to those used for the main click-train oddball experiments.

### Data collection and pre-processing

#### EEG recording.

EEG recording was conducted using a 64-channel electrode cap (10–20 system, NeuSen W series, Neuracle, China) connected to a wireless amplifier (NeuSen W series, Neuracle, China). Of the 64 channels in the electrode cap, five electrodes (‘ECG’, ‘HEOR’, ‘HEOL’, ‘VEOU’, ‘VEOL’) were dedicated to recording electrooculography (EOG) and electrocardiogram (ECG), leaving 59 channels (refer to Fig 2B) for processing signals. Additionally, two electrodes positioned slightly behind and in front of the Fz electrode served as the reference electrode (REF) and the ground electrode (GND), respectively, for referencing and grounding purposes. EEG data and the time of trial onset were simultaneously recorded at a sampling rate of 1 kHz. Participants were comfortably positioned with head support in an acoustically isolated room during the experimental sessions. They were instructed to minimize eye blinks and facial movements to reduce artifacts.

The raw EEG data were band-pass filtered between 0.5 and 40 Hz. Continuous EEG data were epoched from −750 to 1,000 ms relative to the onset of each deviant and the immediately preceding standard stimulus. Baseline correction was then applied by subtracting the mean responses within the rest signals before the sequence start. To identify and reject motion artifacts, a relative threshold approach was utilized for each epoch. Epochs containing data points exceeding predefined thresholds were excluded. The relative threshold was determined based on the percentage of bad samples within a trial. A sample was flagged as a “bad sample” if it fell outside the range of “Mean” ± 3 × “SD” for a trial (epoch) across specific channels. Trials with over 20% of bad samples were categorized as “bad trials”. Additionally, channels with over 10% of bad trials were marked as “bad channels”. Initially, bad channels were excluded by evaluating bad samples using data from all channels. Subsequently, bad trials were excluded from all channels by computing bad samples using data from the remaining good channels. Finally, independent component analysis (ICA) was employed using FieldTrip Toolbox [60] to remove additional artifacts, specifically those related to EOG and ECG.

#### ECoG recording.

ECoG recording involved positioning a reference wire from the ECoG array into the subdural space, with the ground wire connected to a titanium screw at the front of the skull. Lead wires from the ECoG array were then connected to micro connectors (ZIF-Clip headstage adapters, Tucker-Davis Technologies, TDT, Alachua, FL). The signals were subsequently amplified using an amplifier (RZ5, TDT), digitally sampled at a rate of 12 kHz, and stored on hard drives for later detailed analysis.

The raw ECoG signal underwent down-sampling to 600 Hz and was segmented into epochs lasting 1.75 seconds each (comprising 0.75 s before the deviant or standard stimulus and 1 s after). Subsequently, baseline correction was applied, and any identified bad channels or trials were excluded from further analysis. The pre-processing methodology mirrored that used for EEG data.

#### Extracellular recording.

Extracellular recording involved the use of Version 3A Neuropixels silicon probes (Imec, Leuven, Belgium; https://github.com/cortex-lab/neuropixels/wiki/About_Neuropixels) during each session. Each puncture was precisely positioned based on pre-established markers. A flexible ground wire was soldered to the electrode’s PCB, with the ground and reference contacts shorted. Throughout the experiments, this ground wire was connected to a titanium screw connector at the front of the skull. The probes were cautiously inserted vertically through 0.3−0.5 mm long slots in the dura, avoiding visible blood vessels to minimize damage to brain tissue. A single-axis motorized micromanipulator, controlled from outside the soundproofed room, facilitated the insertion process. The average insertion depth for A1 was approximately 2 ± 0.1 mm (mean ± SD, *n* = 12), MGB: 7 ± 0.5 mm (*n* = 14), IC: 6.1 ± 0.4 mm (*n* = 12) below the brain surface, in accordance with a standard rat brain atlas [61]. White noise stimuli were employed to ascertain the presence of auditory responses.

The signals were recorded using the Neuropixels PXIe control system (Imec, Leuven, Belgium) at a sampling rate of 30 kHz for spike data and 2.5 kHz for local field potentials (LFPs). Neuronal spike trains were sorted offline using the Kilosort algorithm, followed by manual curation in Phy [62]. Subsequently, for each neuron, the response window (0–100 ms) and baseline window (–20 to 0 ms) were defined relative to the onset of each standard or deviant stimulus. As the response and baseline windows were not equal in duration, spike counts in each window were converted to firing rates (spikes per second) prior to analysis. We also calculated the Poisson cumulative distribution function from the baseline to assess the likelihood that the observed spike counts at each time point could occur randomly [63], and determined the latency as the time point of the first spike where the Poisson probability falls below the specified threshold (1e−3). Neurons were classified as responsive according to three criteria: 1) the firing rate in the response window significantly exceeded that in the baseline window (right-tailed paired *t* test, α = 0.05); 2) the response latency could be detected; 3) the average spike count in the response window had to be at least one spike higher than that in the baseline window. Neurons meeting these criteria were defined as auditory-responsive and were utilized for subsequent analytical calculations. For LFPs, we downsampled the signals to 300 Hz for subsequent current source density (CSD) analysis.

All recordings of AC data utilized for subsequent analysis were conducted exclusively from the A1 in the left hemisphere, identified by the tonotopic gradient of CF. Additionally, upon completion of the entire recording session, the Neuropixels probes were coated with DiI and inserted into the MGB and IC areas for precise positioning [30]. MGB recordings included neurons located mainly in the ventral and dorsal subdivisions of MGB, whereas IC recordings included neurons located mainly in the central and external subdivisions of IC, as verified by stereotaxic coordinates, electrode depth, and histological reconstruction of probe tracks. Subsequently, the rat underwent transcardial perfusion in a fume hood. An example of the recording location in the rat cortex is illustrated in Fig 4C.

In total, recordings were obtained from 671 neurons in the A1, 632 neurons in the MGB, and 940 neurons in the IC. All recorded neurons were included in the subsequent analysis. Furthermore, a subset of these neurons underwent control experiments utilizing complex tone oddball paradigms (*Session 5* in Extracellular recording experimental procedures). For the primary control analysis, to assess the statistical significance of the CSI for each neuron, we employed a nonparametric bootstrap procedure with 1,000 iterations. Trials were resampled with replacement within each stimulus-role condition, and CSI was recomputed for each bootstrap iteration. A neuron was classified as having a significantly positive CSI when the lower bound of the 95% bootstrap confidence interval exceeded zero. Only neurons with CSI values significantly greater than zero were included in this control dataset. This procedure yielded 94 neurons in A1, 109 neurons in MGB, and 140 neurons in IC. To further reduce possible circular-selection bias, we performed a split-half cross-validation analysis on the complex-tone trials. For each neuron, trials were randomly divided into two independent subsets. Neurons were first selected using one subset of the trials based on significantly positive CSI, applying the same bootstrap criterion described above. CSI was then recomputed for these selected neurons using the independent held-out subset of trials. This procedure allowed us to test whether the positive complex-tone SSA effect remained present in data not used for selection.

### Data analysis

All steps of the mentioned off-line EEG, ECoG, and Extracellular data processing were performed using MATLAB R2022b and the FieldTrip toolbox [60].

#### EEG data analysis.

EEG data analysis involved computing the relative response magnitude to quantify the difference between the responses to deviant and standard stimuli, utilizing average evoked response potential (ERP) data from each channel across trials for each subject. For individual subjects, we selected the 0–300 ms window relative to the onset of both deviant and standard stimuli as the calculation window. A grand-average ERP waveform was then obtained by averaging data across a predefined ROI comprising 27 temporal, parietal, and posterior scalp electrodes, selected a priori based on our previous study [9] from each subject (refer to Fig 2D). Furthermore, we computed the mean amplitude of the average response from each channel. For this computation, we applied a sliding window approach with a window size and step size of 5 ms. Subsequently, an independent-samples *t* test with FDR correction was conducted to determine if there was a significant difference between the mean values of the deviant and standard conditions across channels.

For population analysis, we averaged the grand-average waveform across all subjects (refer to Fig 2E–2H). We also calculated the mean value on every grand-averaged waveform using the same sliding window parameters and conducted an independent-samples *t* test with FDR correction to assess the differences between the deviant and standard stimulus conditions across subjects. If the *p*-value for the difference between the deviant and standard stimuli is less than 0.05 during this time period, the corresponding time points are marked in gray.

#### ECoG data analysis.

For each rat, we computed the average ERP from each channel across all trials. Using the time window of 0–200 ms, we calculated the amplitudes for both deviant and standard stimuli. Subsequently, paired *t*-tests were conducted to evaluate whether there were significant differences between the responses to deviant and standard stimuli on each channel.

For population-level analysis, we calculated the mean amplitude of the ECoG waveforms within a 0–200 ms window following the onset of each deviant stimulus and compared it with the mean amplitude during a 50 ms baseline window preceding the stimulus. Channels from each rat where the response exceeded 2 standard deviations above the baseline were selected and pooled together (*N* = 3 rats, *n* = 36 channels). These pooled channels were averaged to create a population-level waveform. In this combined dataset, we followed the same statistical testing approach described above to determine time periods where significant differences existed between deviant and standard stimuli. Time points where the *p*-value for the difference between deviant and standard stimuli was less than 0.05 during this period were marked in gray.

#### Extracellular data analysis.

For oddball paradigms, we employed one SSA index consistent with previously published work [20,33,64,65] to describe the adaptive degree of SSA.

The Common-SSA Index (CSI) was computed using the formula:


$$\begin{array}{c} CSI=\frac{d_{\text{1}}+d_{\text{2}}-s_{\text{1}}-s_{\text{2}}}{d_{\text{1}}+d_{\text{2}}+s_{\text{1}}+s_{\text{2}}} \end{array}$$

Here, s_1_ and d_1_ represent the neuronal responses to the first click train in an oddball pair when it was the standard and deviant, respectively, while s_2_ and d_2_ denote the responses to the second click train in the pair under the same conditions. The SSA index CSI equals 1 when adaptation is complete (i.e., no response to the standard, and significant response to the deviant), and 0 when there is no adaptation (i.e., the response to the standard and deviant is equal). Meanwhile, to assess whether the CSI of each unit was significantly greater than zero, we used a nonparametric bootstrap procedure with 1,000 iterations. Trials were resampled with replacement within each stimulus-role condition, and the CSI was recalculated for each iteration. A unit was considered to have a significantly positive CSI when the lower bound of the resulting 95% bootstrap confidence interval exceeded zero. Neurons passing this criterion were highlighted in red to indicate their distribution in the corresponding figure.

Following spike count computation, the window was consistently chosen from 5 to 200 ms relative to the onset of the deviant stimuli or the ninth standard stimuli. It should be noted that all data analyzed using ANOVA or *t* test in this study were assessed for normality using the Shapiro–Wilk test prior to analysis. When normality was violated, the Wilcoxon signed-rank test or Mann–Whitney *U* test was used.

For all peri-stimulus time histograms (PSTHs), the bin size was set to 5 ms, step was set to 5 ms. To generate the PSTH heatmap (Fig 5), the neural responses of the population to each deviant and standard stimulus were quantified and normalized using z-score transformation. The z-scored responses to standard stimuli were subtracted from those to deviant stimuli for each neuron. The resulting difference values were then used to construct the heatmap, where each row represents an individual neuron and the color scale reflects the z-scored difference.

#### Current source density analysis.

For laminar recordings in A1, Neuropixels probes were inserted as close as possible to perpendicular to the cortical surface. After recording, probe tracks were reconstructed histologically to verify the trajectory through auditory cortex. Layer assignment was based on two complementary criteria: recording depth along the probe relative to published rat auditory-cortex layer depths, and the noise-evoked CSD profile calculated from LFP responses (example shown in S6 Fig). Based on the combination of anatomical depth and CSD sink/source patterns, neurons were assigned to supragranular, middle, or deep cortical compartments.

One-dimensional current source density (CSD) profiles were calculated from the second spatial derivative of the LFP [31,66–68], which can be approximated using the following formula:


$$\begin{array}{c} \frac{\partial^{\text{2}}\theta (z)}{\partial z^{\text{2}}}\approx \frac{\theta (z+n\Delta z)-\text{2}\theta (z)+\theta (z-n\Delta z)}{{\left(n\Delta z\right)}^{\text{2}}} \end{array}$$

where θ is the field potential, z is the spatial coordinate perpendicular to the cortical laminae, Δz is the spatial sampling interval (Δz = 80 µm), and n is the differentiation grid (*n* = 2). To reduce spatial noise, a five-point Hamming filter was applied [67]. The LFP data used for this analysis were selected from channels with an 80 µm sampling interval, as we used a dense channel configuration with Neuropixel electrodes. The sound stimulus was a 100-ms noise burst. In the resulting laminar CSD profiles, current sinks are shown in red and current sources in blue. Visualization of laminar profiles was enhanced using linear channel interpolation.

In this text, we describe and discuss our A1 results in terms of cortical layers. Our layer-assignment procedure is primarily based on two criteria: first, we compared the electrode penetration depth with previously published depth information of cortical layers [69,70]; second, we utilized CSD profiles. Specifically, our CSD sink latency depth profiles showed excellent agreement with those previous studies [31,67,68,71], from which we adopted their layer classification methodology. Based on these two criteria, we divided the cortical layers into three parts (supra: layer I/II, middle: layer III/IV, and deep: layer V/VI) for our analysis.

## Supporting information

S1 Fig Peripheral auditory representations of click-train stimuli. Cochleagram representations of the 10 click trains and two additional spectrally matched click-train pairs computed using an ERB-spaced gammatone filterbank implemented in the Auditory Modeling Toolbox (AMT).
(EPS)

S2 Fig Population neuronal response to oddball paradigm with temporally merged click-train in A1 and subregions of IC, MGB. **(A)** Distribution of CSI in A1 (*n* = 671), MGD (*n* = 398), MGV (*n* = 225), EIC (*n* = 524), and CIC (*n* = 414) in the oddball paradigm (RN_10_, TW_50_). Arrows indicate the mean. Red bars highlight neurons with CSI significantly greater than 0, determined by a nonparametric bootstrap procedure with 1,000 iterations (*p* < 0.05). **(B, C)** Distribution of CSI values for two additional sound pairs in the oddball paradigm (RN60, Con) and (Asc, TW_30_). Source data are available in Zenodo (see Data availability).
(EPS)

S3 Fig Population neuronal responses to complex tones and temporally merged click-train in the oddball paradigm. **(A)** Population PSTHs displaying responses to two complex tones as deviant (red) and standard (blue) stimuli across three auditory stations: A1 (*n* = 94, top row), MGB (*n* = 109, middle row), and IC (*n* = 140, bottom row). Only neurons with significant CSI values, determined by a nonparametric bootstrap procedure with 1,000 iterations (*p* < 0.05), were included in these datasets. **(B)** Distribution of CSI in A1 (top row), MGB (middle row), and IC (bottom row). Arrows point to the mean. Red bars highlight neurons with CSI significantly greater than 0, determined by a nonparametric bootstrap procedure with 1,000 iterations (*p* < 0.05). **(C)** Comparison of CSI among A1, MGB, and IC. Error bars represent standard error. ***p < 0.001, ANOVA with post-hoc test. **(D–F)** Population responses from the same datasets featured in panels (A-C) to the sound pair in the oddball paradigm (RN_10_, TW_50_): Adheres to the same conventions as panels (A-C). **(G–I)** Population responses from the same datasets featured in panels (A-C) to the sound pair in the oddball paradigm (RN_60_, Con): Adheres to the same conventions as panels (A-C). **(J–L)** Population responses from the same datasets featured in panels (A-C) to the sound pair in the oddball paradigm (Asc, TW_30_): Adheres to the same conventions as panels (A-C). Source data are available in Zenodo (see Data availability).
(EPS)

S4 Fig Population neuronal responses to spectrally matched click-train pairs in the oddball paradigm recorded in IC, MGB, and A1. **(A)** FFT magnitude spectra of the first stimulus pair used in **(B–D)**. Black solid and orange dashed lines represent the two stimuli within the pair, which overlap because the stimuli were matched in long-term spectral magnitude. (B) Population PSTHs showing averaged neuronal responses to S-Pair1A (left) and S-Pair1B (right) as deviant (red) and standard (blue) stimuli in A1 (*n* = 466, top row), MGB (*n* = 348, middle row), and IC (*n* = 634, bottom row). (C) Distribution of CSI values calculated for neurons in A1, MGB, and IC within 5–200 ms from stimulus onset. Red bars indicate neurons with CSI significantly greater than 0, determined by a nonparametric bootstrap procedure with 1,000 iterations (*p* < 0.05). Arrows indicate the mean. (D) Comparison of CSI values among A1, MGB, and IC. Error bars represent SEM. ***p < 0.001, ANOVA with post hoc test. **(E–H)** Population responses to a second spectrally matched stimulus pair in the oddball paradigm, following the same conventions as panels (A–D). Source data are available in Zenodo (see Data availability).
(EPS)

S5 Fig Effect of AC cooling on the response in A1 and oddball paradigm with complex tones in MGB. **(A)** Neuronal responses in the AC before, during, and after AC cooling. Raster plots display the responses to the noise sound (duration = 100 ms) in each of the three conditions: before cooling (top row), during cooling (middle row), 2 hours after cooling (bottom row). **(B–D)** Population responses to two complex tones in the oddball paradigm, adhering to the same conventions as Fig 6, *p* = 0.53, paired *t* test. Source data are available in Zenodo (see Data availability).
(EPS)

S6 Fig Histological reconstruction and laminar CSD profile of a Neuropixels penetration in A1. **(A)** Histological reconstruction of the Neuropixels probe track in auditory cortex. The orange signal marks the probe track within AC, and the dashed line indicates the estimated trajectory of the probe. Scale bar, 1 mm. **(B)** Laminar LFP and current source density profiles evoked by noise stimulation from the same penetration. Left, LFP responses recorded along the probe from superficial to deep cortical depths. Middle, schematic of the 384-channel Neuropixels probe, with channels selected every 80 µm for CSD analysis. Right, CSD profile calculated from the selected LFP channels and smoothed for visualization. Sources are shown in blue and sinks in red. Horizontal dashed lines indicate approximate boundaries between cortical layers. Vertical dashed lines indicate noise onset. Source data are available in Zenodo (see Data availability).
(EPS)

S1 Table Description of the 10 sets of click trains. (DOCX)

S1 Audio Audio files of click-train stimuli and spectrally matched control stimuli. This file contains the 10 click trains used as auditory stimuli and two additional pairs of click trains with identical frequency-domain magnitude spectra but different time-domain structures. **001_Con.wav**: click train with a constant inter-click interval (ICI) of 4 ms; **002_Ascend.wav**: click train with the ICI progressively increasing from 2 to 6 ms; **003_Descend.wav**: click train with the ICI progressively decreasing from 6 to 2 ms; **004_TW10.wav**: click train with the ICI varying randomly between 3.6 and 4.4 ms (4 ± 10%); **005_TW30.wav**: click train with the ICI varying randomly between 2.8 and 5.2 ms (4 ± 30%); **006_TW50.wav**: click train with the ICI varying randomly between 2 and 6 ms (4 ± 50%); **007_TW70.wav**: click train with the ICI varying randomly between 1.2 and 6.8 ms (4 ± 70%); **008_RN10.wav**: click train with the ICI varying randomly within the range of 3.6–4.4 ms (4 ± 10%); **009_RN40.wav**: click train with the ICI varying randomly within the range of 2.4–5.6 ms (4 ± 40%); **010_RN60.wav**: RN60 click train with the ICI varying randomly within the range of 1.6–6.4 ms (4 ± 60%); **011_S-Pair1A.wav**: stimulus A of spectrally matched click-train Pair 1, generated using a multi-sine modulation of the inter-click intervals; **012_S-Pair1B.wav**: time-reversed counterpart of S-Pair1A, with the same frequency-domain magnitude spectrum but a different temporal structure; **013_S-Pair2A.wav**: stimulus A of spectrally matched click-train Pair 2, generated using an exponentially varying inter-click interval pattern with added random noise; **014_S-Pair2B.wav**: time-reversed counterpart of S-Pair2A, with the same frequency-domain magnitude spectrum but a different temporal structure.
(RAR)

## Abbreviations

AC: auditory cortex
AMT: auditory modeling toolbox
CF: characteristic frequencies
CSD: current source density
CSI: common stimulus-specific adaptation index
DMS: delayed match-to-sample
EEG: electroencephalogram
ERP: evoked response potential
FRA: frequency response area
IC: inferior colliculus
ICA: independent component analysis
ICIs: inter-click intervals
ISI: inter-stimulus interval
LFPs: local field potentials
MGB: medial geniculate body
PSTHs: peri-stimulus time histograms
REF: reference electrode
SEM: standard error of the mean
SSA: stimulus-specific adaptation
