# Supplementary material for: Distinct millisecond-scale click-train patterns evoke discriminable behavioral and neural responses in humans and rats
Source: PLoS Biol. 2026 Oct 8;24(10):e3004046. doi: 10.1371/journal.pbio.3004046 (PMC13686464; doi:10.1371/journal.pbio.3004046)
Supplement: Supplementary file 1 [file pbio.3004046.s007.docx]

| **Num** | **Name** | **Average ICI (ms)** | **Pulse Num** | **Description** |
| --- | --- | --- | --- | --- |
| 1 | Con | 4 | 51 | ICI remains constant at 4ms |
| 2 | Asc | 4 | 51 | ICI increases progressively from 2 ms to 6 ms (decreasing repetition rate; descending pitch trend) |
| 3 | Dec | 4 | 51 | ICI decreases progressively from 6 ms to 2 ms (increasing repetition rate; ascending pitch trend) |
| 4 | TW_10_ | 4 | 51 | ICI varies randomly between two values: 3.6 ms and 4.4 ms (4±10%) |
| 5 | TW_30_ | 4 | 51 | ICI varies randomly between two values: 2.8 ms and 5.2 ms (4±30%) |
| 6 | TW_50_ | 4 | 51 | ICI varies randomly between two values: 2 ms and 6 ms (4±50%) |
| 7 | TW_70_ | 4 | 51 | ICI varies randomly between two values: 1.2 ms and 6.8 ms (4±70%) |
| 8 | RN_10_ | 4 | 51 | ICI varies randomly within the range of 3.6 ms to 4.4 ms (4±10%) |
| 9 | RN_40_ | 4 | 51 | ICI varies randomly within the range of 2.4 ms to 5.6 ms (4±40%) |
| 10 | RN_60_ | 4 | 51 | ICI varies randomly within the range of 1.6 ms to 6.4 ms (4±60%) |

**S1 Table.**
