# Supplementary material for: Distinct millisecond-scale click-train patterns evoke discriminable behavioral and neural responses in humans and rats
Source: PLoS Biol. 2026 Oct 8;24(10):e3004046. doi: 10.1371/journal.pbio.3004046 (PMC13686464; doi:10.1371/journal.pbio.3004046)
Supplement: Supplementary file 2 [file pbio.3004046.s011.docx]

**Response to reviewers**

Reviewer #1: This manuscript presents an ambitious, multi-level study combining human psychophysics, human EEG, rat ECoG, large-scale extracellular recordings, laminar analysis, and cortical cooling to investigate sensitivity to millisecond-scale temporal configuration in click trains. The cross-species scope and technical breadth are impressive, and the finding that temporally merged click trains can nevertheless be discriminated is interesting and potentially novel.

However, despite the richness of the dataset, the study remains largely descriptive. The central conceptual claim, that millisecond-scale temporal configuration constitutes a feature-like dimension of sound, is not supported by clear mechanistic insight or principled stimulus-to-neural mapping. The links between behavioral, cortical, subcortical, and circuit-level results are often inferential rather than explanatory. As a result, the manuscript does not yet achieve the level of biological insight implied by its title and framing.

Below I outline several major concerns that, in my view, cannot be resolved without substantial redesign or new experiments.

**Our Response**

We thank you for this careful and thoughtful overall assessment of our study. We appreciate your recognition of the breadth of the dataset, the cross-species scope of the work, and the potentially interesting nature of the main phenomenon.

We also agree with your central concern that, in the previous version, the manuscript was framed too strongly relative to the level of mechanistic insight provided by the current data. In particular, we agree that the original wording overstated the extent to which the present study established millisecond-scale temporal configuration as a feature-like auditory dimension supported by a principled stimulus-to-neural computational mapping.

In the revised manuscript, we have therefore substantially reframed the study to make its scope and conclusions more conservative. More specifically, we no longer present the work as establishing a new, purely temporal auditory feature. Instead, we now frame the study more cautiously as demonstrating behavioral and neural sensitivity to different click-train temporal patterns, while explicitly acknowledging that these patterns also carry associated spectrotemporal consequences. We have revised the title, abstract, introduction, results, and discussion accordingly.

In addition to this conceptual reframing, we have also added a new experiment using stimuli with matched spectral content but different temporal structures. This new result does not by itself resolve all of the mechanistic issues you raised, but it does strengthen the revised manuscript by showing more directly that temporal organization itself contributes importantly to the observed effects, while remaining consistent with the more cautious overall interpretation of the study.

More broadly, we now state more explicitly that the present study is primarily a multi-level phenomenological and systems-level characterization, rather than a complete mechanistic dissection of the encoded temporal statistics or underlying circuit computations. We also now discuss more clearly the main unresolved issues raised by your review, including the specific acoustic/statistical variables driving discrimination, the interpretation of behavioral–neural dissociations, the limitations of cross-species inference, and the uncertainty in the mechanistic interpretation of the cooling results. These points are now presented as limitations of the present study and as important directions for future work.

Accordingly, in the revised manuscript we have substantially narrowed the central claims, added new supporting data, and improved the discussion of the limits of the current dataset. We believe the revised version now presents a more balanced and defensible contribution: not a definitive mechanistic account of a new auditory feature, but a broad experimental demonstration that different click-train temporal patterns can drive discriminable perceptual and neural responses, with these effects becoming more prominent in higher auditory structures.

We are grateful for this comment because it prompted a substantial improvement in the framing, experimental support, and interpretive balance of the manuscript.

1. Lack of explicit explanation of what temporal statistics are encoded: The authors define "temporal configuration" as the ordering of inter-click intervals within a temporally merged click train, and they convincingly show that differences in such configurations can drive behavioral discrimination and neural deviance responses. They further argue, at a conceptual level, that predictive coding and corticothalamic feedback are likely involved. However, the manuscript does not identify which statistical properties of the click trains are actually encoded or compared by the auditory system. Across experiments, different click trains are effectively treated as categorical auditory objects, rather than as stimuli parametrized along defined temporal dimensions.
Specifically:
- No explicit temporal statistic is tested or quantified, such as variance, monotonicity, transition probability, entropy, or higher-order correlations.
- Neural responses are not related to any formal description of the temporal structure.
- The study does not examine whether neural sensitivity varies continuously with any measurable temporal parameter.
As a result, it remains unclear how millisecond-scale temporal structure is represented in neural activity, beyond the observation that some configurations elicit stronger deviant responses than others. The claim that temporal configuration is encoded as a feature therefore remains descriptive rather than mechanistic.

What would be needed to address this concern:
- Parametric manipulation of a single temporal statistic while holding others constant.
- Regression or encoding analyses relating neural responses or CSI values to explicit temporal metrics.
- Model-based analyses comparing alternative candidate statistics that could explain behavioral and neural discriminability.
These analyses would require substantial additional work and are not straightforward extensions of the current dataset.

**Our Response**

We thank you for this important and constructive comment. We agree with the central point. Although the present study demonstrates that different click-train temporal patterns can drive reliable behavioral discrimination and differential neural responses, it does not yet identify which formal temporal statistics are explicitly encoded or compared by the auditory system. In particular, the current dataset does not determine whether the relevant representations depend on statistics such as variance, monotonicity, transition probability, entropy, higher-order temporal correlations, or other candidate descriptors.

In the revised manuscript, we have therefore substantially narrowed and clarified our claims. Specifically, we no longer present the study as establishing that a mechanistically defined temporal feature has been identified or that the neural system is shown here to encode a specific temporal statistic. Instead, we now frame the work more cautiously as demonstrating sensitivity to different click-train temporal patterns, defined operationally by the arrangement of inter-click intervals within temporally merged click trains.

We also now state explicitly that the present study treats these click-train patterns as a structured stimulus set rather than as points along a formally parameterized temporal feature axis. Accordingly, we acknowledge that the current results are descriptive at the level of stimulus-class sensitivity and do not yet provide a principled mapping from specific temporal statistics to behavioral or neural responses.

In the revised Discussion and Limitations sections, we now make clear that resolving this issue would require the kinds of analyses and experiments you suggest: parametric manipulation of individual temporal statistics while controlling others, regression or encoding analyses relating neural responses or CSI values to explicit temporal metrics, and formal model comparison among alternative candidate descriptors. We agree that such work would require substantial additional experimental and analytical effort and is not a straightforward extension of the present dataset.

Accordingly, we have revised the manuscript throughout to avoid overstating the mechanistic implications of the present results. Our conclusion is now more limited and, we believe, more accurate: the current study establishes that distinct millisecond-scale click-train temporal patterns can produce differential behavioral and neural responses across multiple levels of the auditory system, but it does not yet determine which specific temporal statistics are encoded or how those statistics are represented computationally.

We are grateful for this comment because it helped us sharpen the conceptual scope of the manuscript and more clearly separate the phenomenon demonstrated here from the mechanistic questions that remain open.

**Revised text in the manuscript:**

“A key open question is which acoustic or temporal statistics drive the observed behavioral discrimination. Although the present study shows that discrimination cannot be explained simply by mean inter-click interval or by long-term spectral matching, it does not identify the specific computational variable extracted by the auditory system. Several candidate accounts remain possible, including temporal regularity-related cues, pitch-like perceptual consequences of fast click trains, modulation-based representations, asymmetric temporal filtering/integration, or other higher-order temporal descriptors. Because the current stimulus set was not designed to isolate these alternatives parametrically, the present data do not allow a decisive distinction among them.” (P.25, Line 17- P.26, Lines 4)

“First, although our results show that different click-train temporal patterns can drive reliable behavioral and neural differences, the present dataset does not identify which specific temporal or acoustic statistics are encoded or compared by the auditory system. The stimulus sets used here were not designed to isolate a single descriptor, such as temporal regularity, monotonicity, transition probability, entropy, higher-order temporal correlations, pitch-related cues, or modulation-based representations, while holding others constant. Thus, the current findings demonstrate sensitivity to click-train temporal patterns, but do not yet establish the precise computational variables underlying that sensitivity.” (P.31, Lines 2-11)

2. Insufficient justification of stimulus design and unexplained behavioral-neural dissociations: The manuscript employs a large set of click trains (Asc, Dec, TW10-70, RN10-60), which are clearly described procedurally. However, the rationale for choosing these specific configurations, and their hypothesized perceptual or computational relevance, is not articulated. More importantly, several results reveal clear dissociations that are not addressed. For example:
- Human participants fail to behaviorally discriminate TW10 versus TW30, yet these same stimuli elicit robust neural differences in EEG oddball paradigms.
- The manuscript does not discuss what such dissociations imply about perceptual access, task demands, or neural readout.
Without a principled account of why particular configurations should be similar or different, both behaviorally and neurally, the interpretation of these results remains ambiguous. In the absence of such an account, the millisecond-scale temporal structure risks becoming incidental rather than explanatory.

What would be needed to address this concern:
- A theoretical or computational justification for the chosen stimulus classes.
- Analyses explicitly comparing behavioral discriminability with neural discriminability across stimulus pairs.
- Discussion, or direct testing, of whether neural differences reflect subthreshold representations, predictive mismatch, or task-independent encoding.
Addressing these points would likely require new behavioral paradigms or explicit modeling, beyond revision-level changes.

**Our Response**

We thank you for this important and very helpful comment. We agree with the central point. In the previous version, the manuscript did not sufficiently distinguish between demonstrating sensitivity to different click-train temporal patterns and identifying the specific temporal statistics encoded or compared by the auditory system. Our current dataset shows that different click-train temporal patterns can drive behavioral discrimination and differential neural responses, but it does not yet establish which formal temporal descriptors—such as variance, monotonicity, transition probability, entropy, or higher-order correlations—are explicitly extracted, nor does it define a continuous temporal feature space along which neural sensitivity can be mapped mechanistically.

In the revised manuscript, we have therefore substantially narrowed and clarified our claims. Specifically, we no longer present the study as identifying a mechanistically defined temporal feature dimension, nor do we claim that the present data reveal how millisecond-scale temporal structure is formally encoded in neural activity. Instead, we now describe the work more cautiously as showing behavioral and neural sensitivity to different click-train temporal patterns, defined operationally by the arrangement of inter-click intervals within temporally merged click trains.

We have also revised the Discussion to explicitly acknowledge the precise limitations you raise. We now state clearly that: (1) the current stimulus set was not designed to isolate a single temporal statistic while holding others constant; (2) the present neural analyses do not relate response magnitude or CSI values to a formal set of candidate temporal metrics; and (3) the dataset does not allow a decisive comparison among alternative models based on distinct temporal descriptors. We further note that multiple candidate properties may contribute to the observed discrimination and deviance sensitivity, including local interval transitions, monotonic trends, regularity, sequential predictability, higher-order temporal structure, and potentially the spectrotemporal consequences associated with different click timing patterns.

We also agree that the most direct way to address this issue would require a new experimental and analytical framework, including parametric manipulation of individual temporal statistics while controlling others, regression or encoding analyses linking neural responses to explicit temporal metrics, and formal model comparison among alternative candidate statistics that might explain behavioral and neural discriminability. We have now added this point explicitly to the revised manuscript as an important direction for future work. We agree that these analyses would require substantial additional work and are not straightforward extensions of the current dataset.

Accordingly, we have revised the manuscript throughout to avoid overstating the mechanistic implications of the present results. Our conclusion is now more limited and, we believe, more accurate: the current study establishes that distinct millisecond-scale click-train temporal patterns can produce reliable behavioral discrimination and differential neural responses across multiple levels of the auditory system, but it does not yet determine which explicit temporal statistics are encoded or how those statistics are computationally represented. We are grateful for this comment because it helped us sharpen the conceptual scope of the manuscript and more clearly separate the phenomenon demonstrated here from the mechanistic questions that remain open.

**Revised text in the manuscript:**

“The Asc and Dec stimuli therefore represented ordered monotonic interval patterns, the TW stimuli represented two-way interval patterns, and the RN stimuli represented random interval patterns with different degrees of spread. These stimulus classes were chosen to sample several qualitatively different forms of millisecond-scale temporal pattern variation under a shared mean inter-click interval, thereby enabling structured behavioral and neural comparisons across distinct temporal-pattern categories.” (P.7, Line 11-17)

“A related unresolved issue concerns the dissociation between behavioral and neural sensitivity. For example, some stimulus pairs that were weakly discriminable in the delayed match-to-sample task (e.g. TW10 versus TW30 in Fig.1C) nevertheless elicited measurable neural differences (e.g. TW10 versus TW30 in Fig.2G) in the oddball paradigms. This suggests that the neural signals measured here may reflect processing stages that are not identical to explicit perceptual report. Such dissociations could arise if oddball-related responses capture task-independent or pre-attentive sensitivity to click-train pattern differences, context-dependent deviance or predictive mismatch signals, or representations that require additional readout, memory, or decision processes before they influence overt behavior. The present data do not distinguish decisively among these possibilities.” (P.26, Line 4-14)

“Limitations of the present study

Several important limitations of the present study should be acknowledged. First, although our results show that different click-train temporal patterns can drive reliable behavioral and neural differences, the present dataset does not identify which specific temporal or acoustic statistics are encoded or compared by the auditory system. The stimulus sets used here were not designed to isolate a single descriptor, such as temporal regularity, monotonicity, transition probability, entropy, higher-order temporal correlations, pitch-related cues, or modulation-based representations, while holding others constant. Thus, the current findings demonstrate sensitivity to click-train temporal patterns, but do not yet establish the precise computational variables underlying that sensitivity. Second, the underlying neuronal circuit mechanisms also remain incompletely resolved. In particular, although cortical cooling provided causal evidence that cortical input contributes to MGB sensitivity under our paradigm, the present data do not determine whether this effect reflects predictive signaling, gain control, adaptation dynamics, changes in inhibitory-excitatory balance, or other forms of context-dependent modulation. Third, cross-species comparisons must be interpreted with particular caution. While humans and rats showed analogous neural sensitivity to the same stimulus classes, we did not behaviorally validate in rats that these click trains occupy the same perceptual regime as in humans, and therefore the present study does not establish perceptual equivalence across species. More broadly, several observed behavioral–neural dissociations indicate that neural sensitivity to temporal-pattern differences may exceed what is accessible under a particular behavioral task, and the current study does not provide a formal model linking perceptual discrimination, population coding, and deviance-related responses across levels of the auditory pathway. Addressing these issues will require future work using more controlled parametric stimulus designs, formal model comparison, direct neural decoding analyses, and more specific circuit manipulations.” (Discussion, P.31-32)

3. Cross-species generality is asserted without validating perceptual equivalence: A central claim of the paper is that sensitivity to temporal configuration is conserved across species. This claim is based on the use of identical stimuli in humans and rats, and the observation of MMN or MMN-like responses in both. However, the perceptual status of these stimuli is never validated in rats. In humans, the study explicitly relies on the assumption that the click trains are perceptually merged and that individual ICIs are not consciously detectable. Whether the same assumption holds in rats is not tested.

Given known species differences in temporal resolution and auditory processing, it is not trivial to assume that identical stimuli occupy equivalent perceptual regimes across species. Without behavioral validation or alternative controls, the claim of conserved temporal configuration processing remains indirect.

What would be needed to address this concern:
- Behavioral assays in rats testing discrimination or generalization across click-train configurations.
- Physiological validation that the stimuli fall below perceptual segregation thresholds in rats.
- At minimum, control stimuli demonstrating that rat responses are not driven by simpler cues.
These additions would constitute major new experiments.

**Our Response**

We thank you for this important and constructive comment. We agree that the cross-species interpretation in the previous version was too strong. Although humans and rats were tested with the same stimulus classes and both showed differential neural responses to changes in click-train temporal patterns, the present study does not establish that these stimuli occupy equivalent perceptual regimes in the two species.

In humans, our interpretation relies in part on psychophysical evidence that these short-ICI click trains are temporally merged under the present stimulus conditions. In rats, however, we did not perform behavioral experiments to determine whether the same click trains are perceptually merged, consciously discriminable, or segregated in an equivalent way. We therefore agree that the present data do not justify a strong claim that temporal configuration processing is conserved at the perceptual level across species.

In the revised manuscript, we have therefore substantially narrowed this claim. Specifically, we no longer state that the study demonstrates conserved perceptual processing of temporal configuration across species. Instead, we now describe the results more cautiously as showing that analogous neural sensitivity to the same classes of click-train temporal patterns can be observed in both humans and rats under the present experimental conditions. We also now explicitly acknowledge that this cross-species comparison is limited by the absence of behavioral validation in rats.

In the revised Discussion and Limitations sections, we now state clearly that establishing perceptual equivalence across species would require substantial additional work, such as rat behavioral discrimination or generalization assays, or other physiological or behavioral controls that more directly assess whether these stimuli fall within a comparable perceptual regime in rats. We agree that such experiments would constitute major additions beyond the scope of the present revision.

Accordingly, we have revised the manuscript throughout to avoid overstating the cross-species implications of the current dataset. Our conclusion is now more limited and, we believe, more accurate: the study demonstrates cross-species similarity in neural sensitivity to these click-train temporal patterns, but it does not yet establish perceptual equivalence or a fully conserved perceptual mechanism across species.

We are grateful for this comment because it helped us clarify an important limitation of the cross-species interpretation and present the comparative findings in a more balanced way.

**Revised text in the manuscript:**

“In awake rats, ECoG recordings also showed context-dependent cortical response differences across click-train conditions (Fig. 3).” (Discussion, P.23, Lines 3-5)

“Third, cross-species comparisons must be interpreted with particular caution. While humans and rats showed analogous neural sensitivity to the same stimulus classes, we did not behaviorally validate in rats that these click trains occupy the same perceptual regime as in humans, and therefore the present study does not establish perceptual equivalence across species.” (Discussion, P.31, Lines 16–20)

4. Cortical cooling results lack a mechanistic account: The cooling experiments provide convincing evidence that corticothalamic feedback contributes to configuration sensitivity in MGB. The authors appropriately interpret this within a predictive coding framework. However, the mechanistic implications of the cooling results are not fully developed. In particular:
- Cooling largely abolishes enhanced deviant responses, but does not clearly enhance standard responses.
- The manuscript does not explain why removal of cortical predictions affects deviant responses selectively.
- Alternative mechanisms, such as gain control, adaptation dynamics, or changes in inhibitory balance, are not explored.
Thus, while the causal involvement of cortex is well supported, the circuit-level computation remains underspecified.

What would be needed to address this concern:
- Analyses separating changes in baseline, standard, and deviant responses.
- Tests of competing mechanistic models that could explain the observed CSI changes.
- Potentially, additional manipulations targeting specific corticothalamic pathways or layers.
These steps would go well beyond clarification or reanalysis.

**Our Response**

We thank you for this important and constructive comment. We agree that, although the cooling experiments provide strong causal evidence that cortical feedback contributes to MGB sensitivity to click-train temporal patterns, the previous version of the manuscript did not sufficiently separate this causal conclusion from a fully specified mechanistic interpretation.

In the revised manuscript, we have therefore substantially narrowed and clarified our interpretation of the cooling results. Specifically, we no longer present these data as establishing a unique predictive-coding mechanism. Instead, we now state more cautiously that cortical input contributes importantly to MGB sensitivity under our paradigm, while the precise circuit-level computation remains unresolved.

We also agree that one important feature of the data is that cooling strongly reduces the enhanced deviant responses, without providing a comparably clear enhancement of standard responses. In the revised manuscript, we now avoid treating this pattern as direct proof of prediction-specific signaling. Instead, we describe it more conservatively as showing that cortical feedback contributes to the context-dependent expression of deviant-enhanced responses in MGB under the oddball conditions used here. We further acknowledge that the present data do not determine why this effect appears more evident in deviant than in standard responses.

Importantly, we now explicitly recognize alternative interpretations. In addition to a predictive-coding account, the cooling-induced reduction in CSI could in principle reflect changes in context-dependent gain control, altered adaptation dynamics across standard and deviant conditions, shifts in inhibitory-excitatory balance, or other forms of contextual modulation. We have therefore revised the manuscript so that predictive coding is no longer presented as the only plausible mechanistic explanation of the cooling effect.

We also agree that the most direct way to resolve this issue would require analyses and experiments that go beyond straightforward revision, including separate quantification of baseline, standard, and deviant response changes under cooling, formal comparison of competing mechanistic models, and more selective manipulations of corticothalamic pathways or layers. We now state this explicitly as an important direction for future work.

Accordingly, our revised conclusion is more limited and, we believe, more accurate: the cooling experiments provide strong causal evidence that cortical feedback contributes to MGB sensitivity to click-train temporal patterns, but they do not yet establish the specific circuit mechanism underlying this effect.

We are grateful for this comment because it helped us present the cooling results in a more balanced and mechanistically cautious way.

**Revised text in the manuscript:**

“Thus, cortical feedback contributes importantly to MGB sensitivity to these click-train temporal patterns, but the present data do not determine the specific circuit mechanism underlying this effect.” (P.21, Lines 5–7)

“Second, the underlying neuronal circuit mechanisms also remain incompletely resolved. In particular, although cortical cooling provided causal evidence that cortical input contributes to MGB sensitivity under our paradigm, the present data do not determine whether this effect reflects predictive signaling, gain control, adaptation dynamics, changes in inhibitory-excitatory balance, or other forms of context-dependent modulation.” (Discussion, P.31, Lines 11–16)

In summary, this manuscript presents a compelling collection of phenomena and an impressive experimental effort. However, the central claims regarding feature-like encoding of millisecond-scale temporal configuration, hierarchical computation, and cross-species conservation are not supported by sufficient mechanistic insight or principled stimulus-response mapping. Addressing the major concerns outlined above would require substantial new experiments, theoretical development, and modeling, rather than revision of presentation or analysis alone. For this reason, I recommend rejection at this stage. That said, the dataset is rich, and with a more focused scope or a clearer mechanistic framework, parts of this work could form the basis of a strong future manuscript.
**Our Response**

We thank you for this thoughtful and constructive overall assessment of our study. We appreciate your recognition of the breadth of the dataset, the technical scope of the work, and the potential value of the phenomena described here.

We also understand and take seriously your central concern: namely, that the previous version of the manuscript framed the study too strongly relative to the level of mechanistic insight and formal stimulus-response mapping actually provided by the current data. In particular, we agree that the original presentation overstated the extent to which the study established feature-like encoding of millisecond-scale temporal configuration, a fully mechanistic hierarchical computation, or conserved perceptual processing across species.

In the revised manuscript, we have therefore substantially narrowed the scope of our claims. We no longer describe the study as establishing a new or purely temporal feature of sound, nor do we claim that the present data provide a complete mechanistic account of how click-train temporal patterns are encoded. Instead, we now frame the work more cautiously as a multi-level demonstration that distinct click-train temporal patterns can drive measurable behavioral and neural differences across multiple stages of the auditory system, while explicitly acknowledging that the current dataset does not yet determine which formal temporal statistics are encoded, how those statistics are represented computationally, or whether equivalent perceptual regimes are present across species.

In addition, beyond reframing and clarification, we have strengthened the revised manuscript in several substantive ways. These include new acoustic and peripheral-auditory characterizations of the stimulus set, a new experiment using stimuli with matched spectral content but different temporal structures, clarification of the behavioral task structure and criterion-free sensitivity analysis, a more conservative interpretation of the human EEG, rat ECoG, cortical cooling, and IC control analyses, and an expanded Limitations of the present study section that directly addresses the unresolved mechanistic issues raised in your review.

We fully agree that a more decisive mechanistic resolution of the major issues you outline would require substantial additional work, including new behavioral paradigms, explicit modeling, more formal stimulus parameterization, and more selective circuit-level manipulations. We now state this clearly in the revised manuscript and present these as important directions for future study.

Accordingly, while the revised manuscript remains based on the same core dataset, we believe it now presents a substantially more accurate and appropriately bounded contribution: not a definitive mechanistic account of a new auditory feature, but a rich and systematically reframed study showing that different click-train temporal patterns can produce reliable behavioral and neural differences, with these effects becoming more prominent in higher auditory structures and being influenced by corticothalamic feedback.

We are very grateful for your careful reading and for the clarity of your critique, which has helped us substantially improve the scope, balance, and scientific precision of the manuscript.

Reviewer #2: The paper is an extensive study of the coding of click trains with different types of structure in human as well as in rat auditory systems. This is a valuable dataset, and the interspecies comparison is interesting and done reasonably well given the inherent difficulties in such endeavor.

Nevertheless, the manuscript has a significant weakness. This is the claim that the authors study in some way purely temporal aspects of sound organization. While the authors manipulate purely time variables, the inevitable duality of time and frequency implies that they also modify spectral features of these sounds. See below for specific comments. The authors should at least run their stimuli through one of the ubiquitous toolboxes simulating peripheral auditory processing (e.g. <https://amtoolbox.org/>) to get a valid spectro-temporal description of their stimuli. For example, the CON stimulus has a strong pitch at 250 Hz and is in fact a purely harmonic stimulus composed of harmonics of a 250 Hz fundamental (all with cosine phase). The ASC and DEC stimuli have a decreasing and an increasing pitch, doing from 160 Hz to 500 Hz or back (corresponding to periods of 6 ms and 2 ms). They are constructed in the frequency domain from chirps that move appropriately in frequency, composing their individual harmonics at each point in time. The random stimuli have similar spectro-temporal interpretation.

I would urge the authors to reduce the scope of their claims (see the major comment on the discussion). There is no 'novel acoustic feature of sounds' - they study a range of time variations that have been previously studied in some detail. The beauty of this manuscript is in the extensive manipulations and the range of parameters used here, as well as with the understanding that it develops about early processing of these sounds using the rat data. I think this is sufficient novelty, no need to make insubstantial and wrong claims.

**Our Response**

We thank you for this thoughtful and important overall comment. We appreciate your positive assessment of the dataset and the interspecies comparison. We also agree with your central concern that, in the previous version, the manuscript overstated the extent to which the studied manipulations could be interpreted as purely temporal. Although the stimuli were generated by manipulating inter-click timing, we agree that such manipulations inevitably also shape the short-term spectral and broader spectrotemporal structure of the resulting sounds. We therefore agree that it was not appropriate to present the original study as probing a purely temporal auditory dimension or as establishing a “novel acoustic feature of sounds.”

In response, we have substantially revised the conceptual framing of the manuscript throughout. Specifically, we no longer describe the study as identifying a new or purely temporal feature of sound. Instead, we now frame the work more cautiously as examining sensitivity to different click-train temporal patterns, while explicitly acknowledging that these patterns necessarily carry associated spectrotemporal consequences. We have revised the title, abstract, introduction, results, and discussion accordingly, and we have removed or rewritten statements that implied that only temporal structure differed or that other acoustic dimensions were largely controlled in a strict sense.

Following your suggestion, we have also added additional acoustic characterization of the stimulus set and now make the corresponding stimulus audio files and spectral analyses available in the Supporting Information. In the revised manuscript, we explicitly state that differences in inter-click timing give rise not only to temporal differences, but also to associated short-term spectrotemporal representations. These revisions have led us to reinterpret the data more carefully: the observed behavioral and neural discriminability should not be regarded as evidence for a purely temporal effect, but rather as evidence that distinct click-train temporal patterns, together with their associated spectrotemporal consequences, are differentially represented across the auditory system.

In addition, to address this issue more directly, we have added a new experiment using click-train pairs with matched long-term FFT magnitude spectra but different time-domain structures. This additional result strengthens the conclusion that temporal organization itself contributes importantly to the observed effects, while remaining fully consistent with your point that the original click-train stimuli are not spectrally neutral and should not be interpreted as isolating a purely temporal dimension.

We also fully agree that the main novelty of the study does not lie in introducing short-interval click trains per se, nor in claiming a previously unknown acoustic feature. In the revised manuscript, we now emphasize instead what we believe is the more appropriate contribution of the work: the systematic and multi-level demonstration that different click-train temporal patterns are behaviorally and neurally discriminable, that these effects become more prominent in higher auditory structures, and that corticothalamic feedback contributes to this sensitivity.

We are grateful for this comment because it prompted a substantial improvement in the framing, analysis, and interpretation of the manuscript.

Major comments:
Stimuli: the stimuli differ in their short-term spectral structure, and this is reflected also in their neurogram representation. Thus, the fact that they are discriminable is not only due to their temporal structure, but also to the way nearby clicks interact in the spectral domain. Similar effects have been described in other perceptual phenomena that are related to fast click trains, such as precedence effects (where the click representing a direct sound and the click representing an echo may interact monaurally and spectrally; one such paper in humans is Verhulst, Bianchi and Dau 2013, but there are many, starting with the work of Hartung and Trahiotis in the early 2000's, as far as I remember). While this doesn't reduce the importance of the data studied here, it does require a re-interpretation of the results - this is far from being a purely temporal effect. Claims such as (p. 7, end of paragraph) 'only the millisecond-scale temporal configuration differed' are wrong
- such click trains by necessity create also spectral features.

**Our Response**

We thank you for this important comment. We agree with your central point that the differences among our click-train stimuli cannot be interpreted as purely temporal in a strict acoustic or auditory-processing sense. Although the stimuli were generated by manipulating inter-click intervals, such manipulations necessarily alter short-term spectral structure and the resulting spectrotemporal/neurogram representation through interactions among nearby clicks. We therefore agree that our previous wording—especially statements such as “only the millisecond-scale temporal configuration differed”—was too strong and not accurate.

In the revised manuscript, we have addressed this issue in several ways. First, we have substantially revised the framing throughout the paper to avoid describing the studied effects as purely temporal. We now refer more cautiously to differences in click-train temporal patterns, while explicitly acknowledging that these temporal manipulations also give rise to associated spectral and spectrotemporal consequences. Correspondingly, we have removed or rewritten statements implying that other acoustic dimensions were unchanged or largely controlled in a strict sense.

Second, we have added acoustic characterizations of the stimulus set and now provide the corresponding audio files and spectral analyses in the Supporting Information. These additions make clear that the discriminability of the stimuli may arise not only from the temporal arrangement of clicks per se, but also from the spectrotemporal consequences generated by that arrangement. Accordingly, we now interpret the results as reflecting sensitivity to temporally patterned click trains that inherently carry associated spectrotemporal structure, rather than as isolating a purely temporal effect.

Third, we have revised the Discussion to acknowledge more explicitly the broader literature on fast click trains and related perceptual phenomena, including work showing that closely spaced clicks can interact monaurally and spectrally. We now make clear that the present findings should be interpreted within this broader framework, rather than as evidence for a strictly independent temporal feature dimension. In other words, we now interpret our results as showing sensitivity to temporally patterned click trains that inherently carry associated spectrotemporal structure, rather than as isolating a purely temporal effect.

In addition, to address this concern more directly, we have added a new experiment using click-train pairs with matched long-term FFT magnitude spectra but different time-domain structures. This new result further supports the conclusion that temporal organization itself contributes meaningfully to the observed neural differences, while remaining fully consistent with your point that the original click-train stimuli are not spectrally neutral. We also explicitly state that this control does not guarantee identical peripheral or neurogram representations and therefore should not be interpreted as isolating a purely temporal auditory feature.

We are grateful for this comment because it led us to correct an overstatement in the original manuscript and to adopt a more accurate and defensible interpretation of the stimulus manipulations and their perceptual and neural consequences.

**Revised text in the manuscript:**

“Because all ICIs were very short (≤7 ms), these click trains were designed to fall within the temporally merged regime examined in this study. Accordingly, the stimuli differed in their inter-click timing patterns and, as a consequence, also in their associated short-term spectrotemporal representations. All audio files and corresponding spectral analyses of the click-train stimuli are provided in the Supporting Information (S1 File).” (P.7, Line 17-22)

“Several candidate accounts remain possible, including temporal regularity-related cues, pitch-like perceptual consequences of fast click trains, modulation-based representations, asymmetric temporal filtering/integration, or other higher-order temporal descriptors. Because the current stimulus set was not designed to isolate these alternatives parametrically, the present data do not allow a decisive distinction among them.” (P.25, Line 21-P.26, Line 4)

“This broader interpretation is consistent with previous work showing that fast click trains can generate pronounced pitch-related and spectrotemporal effects, and that closely spaced clicks may interact monaurally and spectrally in related perceptual paradigms.” (P.26, Lines 14-17)

“To further examine whether neural sensitivity to click-train temporal patterns could be observed when long-term spectral magnitude was matched, we performed an additional oddball experiment using two pairs of click trains with identical frequency-domain magnitude spectra but different time-domain structures (S3 Fig). For each stimulus pair, FFT spectra were overlapping, confirming matched long-term spectral magnitude across the two sounds within a pair (S3A and S3E Fig.). Despite this spectral matching, population responses still showed deviant–standard differences, particularly in A1 and MGB (S3B and S3F Fig.). CSI distributions were shifted positively in A1 and, to a lesser extent, in MGB, whereas IC showed weaker effects (S3C and S3G Fig.). Across auditory levels, CSI was significantly higher in A1 than in IC for both spectrally matched stimulus pairs (S3D and S3H Fig.). These results indicate that differences in temporal organization can contribute to oddball/context sensitivity even when the paired stimuli are matched in long-term spectral magnitude, although this control does not exclude contributions from peripheral nonlinearities or other spectrotemporal consequences of the waveform.” (P.19, Line 13- P.20, Line 5)

“The additional spectrally matched control experiment further supports this conservative interpretation. In that experiment, stimulus pairs were constructed to have overlapping long-term FFT magnitude spectra while differing in time-domain organization. These stimuli still produced oddball/context-dependent differences in neuronal responses, with stronger CSI in A1 than in IC (S3 Fig). This result suggests that temporal organization contributes to the observed neural sensitivity beyond differences in long-term spectral magnitude alone. However, because matched FFT magnitude does not guarantee identical peripheral or neurogram representations, especially for rapidly varying click trains, this control should not be interpreted as isolating a purely temporal auditory feature. Rather, it strengthens the conclusion that click-train temporal patterning, and its spectrotemporal consequences are differentially represented along the auditory pathway.” (P.26, Line 18-P.27 Line 7)

“Session 6: Spectrally matched click-train oddball procedure. To further test whether click-train temporal organization could affect neural responses when long-term spectral magnitude was matched, we generated two pairs of click trains with identical FFT magnitude spectra but different time-domain structures (S3 Fig). Within each pair, the two stimuli had the same spectral magnitude and overall power but differed in temporal waveform. These stimuli were presented in the same two-sequence oddball design used for Sessions 2–4, with each stimulus serving once as the standard and once as the deviant. All other stimulus parameters and analysis procedures were identical to those used for the main click-train oddball experiments.” (P.39, Line 18- P.40, Line 4)

Behavioral test: The description is unclear, both in the main text and in the Methods. Where all types of sequences compared in a single block or each in its own block? I'm not sure that 50% is the correct random choice in this task - according to the Methods, there were 7 pairs of identical sounds and 13 of different sounds, so that chance probability was 7/20=35%. This may account for the <50% performance in the 'hard' conditions (TW10-Tw30 and the other cases at the left side of Fig. 1C).

**Our Response**

We thank you for pointing out this important issue. You are correct that the description of the behavioral task in the previous version was not sufficiently clear, and that this ambiguity affected the interpretation of the behavioral baseline.

In the revised manuscript, we have rewritten the description of the behavioral experiment in both the main text and the Methods to state the procedure explicitly. In particular, we now clarify that the task used 20 predefined stimulus pairs, including 7 identical pairs and 13 different pairs, and that each pair was presented 40 times per session. All 20 predefined sound pairs were presented within the same active discrimination session, with each pair presented 40 times.

Accordingly, you are correct that the relevant reference proportions were not 50%/50%. Because the stimulus set contained 7 identical pairs and 13 different pairs, the corresponding reference proportions were 35% for “same” trials and 65% for “different” trials. We have therefore revised the corresponding Results and Methods text so that the lower-performance conditions are interpreted relative to the correct task structure rather than an oversimplified 50% criterion.

We are grateful for this comment because it identified a genuine problem in the previous presentation of the behavioral analysis, and correcting it has improved the transparency and accuracy of the manuscript.

**Revised text in the manuscript:**

“On average, the correct response rates for the TW10-TW30, Con-RN10 and RN10-TW10 pairs were 0.34, 0.35, and 0.40 (Fig 1C), respectively, and did not differ significantly from the reference proportion of 0.35 (p > 0.05 for all three pairs, Kolmogorov–Smirnov test). In contrast, the correct response rates for the Con-Asc, Asc-TW30, Asc-Dec, RN60-TW10 and RN60-TW70 pairs were 0.97, 0.96, 0.92, 0.92 and 0.91, respectively (Fig 1C), all significantly greater than the reference proportion of 0.65 (p < 0.01 for all five pairs, Kolmogorov–Smirnov test)” (P.9, Line 6-13)

“We selected 20 different sound pairs, as shown in Fig 1C, each presented 40 times per session. Among these 20 pairs, 7 were identical pairs and 13 were different pairs. Accordingly, the corresponding reference proportions in the task were 35% for ‘same’ trials and 65% for ‘different’ trials .” (P.33, Line 6-9)

In the methods (p. 27) the 'passive listening sessions' section starts by 'Following two active discrimination sessions'; however, in the previous section, only a single active session is described ('Active Listening Session 1'). If the active session was repeated twice, this should be clearly stated.

Our Response

We thank you for carefully reading the Methods section and for catching this inconsistency. You are correct that the previous text referred to “two active discrimination sessions” while only describing “Active Listening Session 1,” which created unnecessary confusion.

In fact, there was only one active discrimination session. The phrase “Following two active discrimination sessions” in the previous version was incorrect and has now been corrected in the revised manuscript. We have also revised the subsequent wording to refer to a single active discrimination session before the passive listening sessions. The sequence of experimental sessions is now accurate and internally consistent.

We are grateful for this comment because it helped us improve the accuracy and clarity of the methodological description.

**Revised text in the manuscript:**

“The study comprised five experimental sessions, including one active discrimination session and four passive listening sessions. (P.32, Lines 19-20, 22)

Active Listening Session (Fig 1B)”

“Following the active discrimination session, four passive listening sessions were conducted.” (P.33, Line 21-22)

P. 10-11, discussion of Fig. 2: Since the sequences used as 'standard' and 'deviant' are different in Fig. 2C-F, it is not surprising that the evoked responses are different. What is the point here? Later in the text, it seems that the authors interpret the higher amplitude responses to the rare stimuli as mismatch negativity (MMN). However, MMN is usually expressed as a negative deflection in the ERP while here the deflection is positive - what is going on? Finally, combining both comments, to show that the difference between standard and deviants are MMN, it is necessary to compare the responses to the same physical sound in the two roles (this is standard in the animal physiology field - see e.g. Parras et al. 2017 and many other papers from the Malmierca lab, Geffen lab, Nelken lab; also in this manuscript, Figs. 4-7). This is possible, but has not been done, in the data in Figs. 2G-H. I would start to describe the data with these panels, and then switch to the 'multi-deviant' paradigm of Fig. 2A-F. See also Näätänen, R., Pakarinen, S., Rinne, T., & Takegata, R. (2004). The mismatch negativity (MMN): Towards the optimal paradigm. Clinical Neurophysiology, 115(1), 140-144. <https://doi.org/10.1016/j.clinph.2003.04.001> for a similar paradigm.

**Our Response**

We thank you for this important and thoughtful comment. We agree that, at the neuronal level, a standard oddball interpretation requires comparing the same physical stimulus in its standard and deviant roles, because single-neuron responses can directly differentiate between two sounds. For this reason, in our extracellular-recording analyses we used the standard two-set oddball design and compared each sound in both roles (Figs. 4–7), which provides a direct measure of stimulus-specific adaptation.

For the human EEG data, however, our rationale followed the logic commonly used in the human EEG oddball literature. At the scalp EEG level, the signal is not typically interpreted as directly reflecting fine-grained coding differences between individual sounds in the same way as neuronal recordings. Instead, one sound is commonly used as the standard and another as the deviant within a single oddball sequence, and the resulting deviant–standard difference is interpreted as an MMN response. In this sense, the purpose of the human EEG oddball design was to test whether a change in click-train temporal pattern evokes an MMN response under oddball context.

At the same time, we agree that this logic should have been explained more clearly in the previous version, and that the distinction between the EEG-level MMN analysis and the single-unit oddball analysis was not presented with sufficient care. Following your suggestion, we have revised the Results and Fig. 2 legend so that the population-level EEG description now begins with comparisons in which the same physical sound is examined across different contextual roles (Figs. 2E–F), and only then moves to the multi-deviant paradigm (Figs. 2G–H). This order makes the logic of the MMN analysis clearer and better aligns the EEG presentation with the standard oddball framework.

Regarding the polarity issue, we agree that this also required clarification. In human EEG, the apparent polarity of the MMN waveform depends on the reference scheme and recording configuration. In our experiment, the EEG signals were referenced to Cz, and under this reference the MMN difference was expressed as a positive-going deflection. We have now clarified this point in the revised manuscript so that the observed polarity is interpreted in light of the reference configuration rather than as contradicting the MMN interpretation.

In the revised manuscript, we now make clear that the human EEG MMN analysis and the neuronal SSA analysis address related but not identical levels of processing: the former reflects scalp-level MMN responses measured under the standard human oddball framework, whereas the latter can more directly assess stimulus-specific neuronal sensitivity by examining each stimulus in both standard and deviant roles.

We are grateful for this comment because it helped us improve both the logic and the presentation of Fig. 2.

**Revised text in the manuscript:**

“EEG signals were recorded using a 64-channel system and were referenced to Cz throughout the experiment.” (P.32, Lines 20-21)

“Fig 2E and 2F show such examples, where significant MMN responses were observed when RN_10_ and Con were paired as the standard and deviant (Fig 2E), and likewise when RN_10_ and TW_30_ were paired as the standard and deviant (Fig 2F). We then examined the multi-deviant oddball paradigm. Fig 2G and 2H show examples in which the standard was TW_30_ (Fig 2G) and RN_10_ (Fig 2H), respectively; the grey horizontal bars indicate time ranges in which the response to the deviant differed significantly from the response to the standard (p < 0.05, independent-samples t-test, with FDR correction applied).” (P.12, Lines 7-14)

P. 11-12, Fig. 3: These are data from an animal model, I would expect the authors to keep to the standards of the field. In auditory cortex, different stimuli are expected to elicit different responses. Thus, I frankly don't understand the point of the figure - what is the message?
Furthermore, in animal studies, responses to deviants are usually larger than responses to standards, but here in essentially all panels it's the reverse - the stimulus that is presumably standard gives rise to larger responses than the deviants. This is incompatible both with previous animal studies, with the claim that these are MMN-like responses, and with the reports of unit recordings in rats in this manuscript.

**Our Response**

We thank you for this important comment. We agree that the previous presentation of Fig. 3 did not make the purpose of this analysis sufficiently clear, and that the interpretation was too strong relative to what these rat ECoG data can support.

Our intention in including Fig. 3 was not to claim that these rat cortical signals should be interpreted in exactly the same way as single-unit oddball responses in animal physiology. Rather, the purpose of this figure was to test whether changes in click-train temporal patterns evoke context-dependent cortical population responses at the mesoscopic signal level in awake rats, thereby providing a cross-species comparison with the human EEG findings.

Importantly, in our rat auditory-cortex ECoG recordings, different click-train stimuli presented in a random, non-oddball context evoked broadly similar responses across recording sites. In other words, the ECoG signal did not show large stimulus-specific differences of the kind commonly observed at the single-neuron level. For clarification, we include below an additional control example showing ECoG responses to randomly presented click trains across 32 channels in one example rat. Each stimulus type was repeated 30 times with 2-s intervals, and the waveforms evoked by different stimuli largely overlapped within each channel, indicating broadly similar population-level evoked potentials across stimulus types.

**Comment Fig 1. ECoG responses to randomly presented click trains in one example rat.**

This observation is consistent with the human scalp EEG data discussed above, where different click-train stimuli also evoked broadly similar population-level responses outside the oddball contrast. Thus, in both EEG and ECoG, the deviant–standard differences are best interpreted as context-dependent population responses rather than as direct evidence for fine-grained stimulus-specific coding at the single-neuron level.

At the same time, we agree with your point that the standard way to demonstrate deviance-related responses in animal recordings is to compare the same physical sound across standard and deviant roles. We have therefore performed an additional ECoG experiment and revised the Fig. 3 analysis accordingly. In the revised version, the ECoG responses are now presented using comparisons in which the same click-train stimulus is evaluated in its standard and deviant roles. This revision makes the logic of the ECoG analysis more consistent with the standards used in animal physiology and with the logic of the single-unit analyses in Figs. 4–7.

We also agree that the polarity and direction of the effect required clearer explanation. Because these are ECoG signals rather than spike counts, a more negative deflection corresponds to a stronger cortical response. In our data, the deviant responses were stronger in the negative direction, and this is the relevant sense in which the deviant responses exceeded the standard responses. We have now clarified this point in the revised manuscript so that the ECoG polarity is interpreted appropriately and is not confused with spike-based response magnitude.

Accordingly, in the revised manuscript, we now present the rat ECoG results more carefully as complementary cortical population-level evidence for context-dependent deviance-related responses. We no longer use Fig. 3 alone as definitive evidence for stimulus-specific neuronal adaptation. Instead, we interpret it together with the extracellular recordings, where each physical stimulus was explicitly examined in both standard and deviant roles.

We are grateful for this comment because it helped us clarify both the interpretation and the presentation of the rat ECoG data.

**Revised text in the manuscript:**

“We used a two-sequence oddball paradigm (Fig. 3A), in which each stimulus in a pair of temporally merged click trains was presented once as the standard and once as the deviant. In one sequence, one click train was repeated nine times as the standard, followed by the other click train as the deviant; this pattern of nine standards followed by one deviant was repeated for 30 cycles. In the second sequence, the standard and deviant roles of the two click trains were reversed.” (P.12, Line 20-P.13, Line 3)

“Across rats, deviant responses were generally stronger in the negative direction than standard responses when the same physical click-train stimulus was compared across its standard and deviant roles. For example, RN10 and TW50 both showed enhanced negative-going responses when presented as deviants compared with when the same stimuli were presented as standards (Fig. 3C). Similar context-dependent differences were also observed for additional stimulus pairs, including RN60–Con and Asc–TW30 (Fig. 3D–E). These results indicate that click-train temporal patterns can evoke deviance-related cortical population responses in awake rats. Together with the human EEG results, these findings suggest analogous neural sensitivity to click-train temporal patterns across species, although the present data do not establish perceptual equivalence between humans and rats.” (P.14, Lines 5-15)

The unit recordings in Figs. 4-6 were performed using the standard approach of comparing the responses to the same stimuli in different conditions, and therefore their interpretation is easier. As expected, deviant responses tend to be somewhat larger than standard responses (controlling for stimulus identity). One comment - in Figs. 5A,B,C,F,G, one stimulus shows std>dev (peak blue above red) and the other dev>std (peak blue below red). Thus, in Fig. 5A, RN10, the peak standard response is larger than the peak deviant response but for the TW50 the reverse occurs, and so on in the other panels. This is expressed also in the lower panels, where there are significant number of neurons in which the early responses show std>dev (deep blue). This suggests that at least some of the effects are non stimulus-specific (e.g. Harpaz et al. 2021 already referred in this manuscript, who provide some discussion of non stimulus-specific effects that can mask as stimulus-specific differences). Such effects may contaminate some of the unit data presented here.

**Our Response**

We thank you for this careful and insightful comment. We agree that, although the unit-recording analyses are more straightforward to interpret because they compare the same physical stimulus across standard and deviant roles, some neurons and time windows show standard > deviant effects, indicating that the observed oddball-related responses are not uniformly stimulus-specific.

At the same time, the majority of neurons showed effects in the expected direction, with deviant responses tending to be larger than standard responses, as reflected by the overall positive shift in the CSI distributions. Thus, while some neurons exhibited standard > deviant effects—particularly in early response windows—the population-level pattern remained biased toward deviant > standard, supporting the conclusion that the population responses show overall deviant-enhanced sensitivity to click-train temporal patterns under oddball context.

We agree, however, that the presence of a subset of neurons showing standard > deviant effects suggests that non-stimulus-specific processes may also contribute. In the revised manuscript, we therefore do not change the reported results, but we have added discussion clarifying that these mixed patterns may reflect the combined influence of stimulus-specific adaptation, broader contextual modulation, adaptation dynamics, latency-dependent effects, or other non-specific factors of the kind discussed by Harpaz et al. (2021). Accordingly, we now interpret the unit data more cautiously, while retaining the main result that the dominant population tendency is toward stimulus-specific deviant enhancement.

We are grateful for this comment because it helped us clarify the interpretation of the unit-recording data and present the result in a more balanced way.

**Revised text in the manuscript:**

“However, a subset of neurons and response windows showed standard > deviant effects, suggesting that the observed oddball-related responses are not exclusively stimulus-specific and may also include contributions from broader contextual or adaptation-related processes.” (P.28, Lines 14-17)

P. 17, Discussion of Fig. S2. I don't accept the conclusion that this shows that IC has reduced ability to discriminate temporal configurations. It could be that IC neurons show strong SSA to only a specific subset of all stimuli - this is implied by the selection procedure of the units studied in this analysis (those who showed large CSI for the complex tone stimuli). Thus, the comparison here is between average over all recorded data (for the click train) and highly selected subset of neurons (for the complex tone stimuli). To argue as they do, the authors have to show that NO SUBSET of neurons in IC show high level of CSI to click trains, and this is obviously wrong (e.g. Fig. 5D, where there is a substantial population of IC neurons with high CSI for two configurations of click trains).

**Our Response**

We thank you for this important comment. We agree that the conclusion in the previous version was stated too strongly.

The purpose of the complex-tone (CT) control analysis was not to argue that IC lacks click-train-sensitive neurons. Rather, it was introduced to address a more specific concern motivated by prior literature, namely that IC neurons often show weaker stimulus-specific adaptation (SSA) than neurons in MGB and A1. For this reason, we examined a subset of neurons showing significantly positive CSI for the CT condition and then asked whether the hierarchical pattern observed for click-train responses persisted within this CT-selected population.

In the revised manuscript, we have therefore substantially softened the interpretation of this analysis. Specifically, we no longer state that IC has a generally reduced ability to discriminate click-train temporal patterns. Instead, we now state more cautiously that the weaker click-train CSI observed in IC relative to A1 is unlikely to be explained solely by the generally weaker SSA often reported in IC. At the same time, we now explicitly acknowledge that this interpretation does not exclude the existence of IC subpopulations with substantial sensitivity to particular click-train pairs, as also suggested by the distribution of CSI values in Fig. 5D.

We are grateful for this comment because it helped us correct an overinterpretation in the original manuscript and present the IC results in a more accurate and balanced way.

**Revised text in the manuscript:**

“Taken together, these control analyses suggest that the weaker click-train CSI observed in IC relative to A1 is unlikely to be explained solely by the generally weaker SSA often reported in IC.” (P.19, Lines 5-7)

“However, this analysis does not exclude the existence of IC subpopulations with substantial sensitivity to specific click-train pairs, nor does it fully exclude contributions from stimulus dependence, population heterogeneity, or other selection-related factors.” (P.19, Lines 7-10)

P. 17-8, Fig. 6: these results are strangely inconsistent with previous results of the same group (Antunes and Malmierca 2011), where cortical cooling resulted also in a significant and consistent reduction of firing rates in the MGB. While I don't doubt the data, this requires an explanation. Maybe cooling was less effective? But then the interpretation of the smaller CSI during cooling is in doubt - maybe this is only due to habituation of the responses (as in Harpaz et al. 2021 )?

**Our Response**

We thank you for this important and thoughtful comment. We agree that the relationship between our cooling results and those reported previously by Antunes and Malmierca (2011) should have been discussed more explicitly in the original manuscript.

In the revised manuscript, we now acknowledge this point directly. Specifically, we note that although cortical cooling reduced CSI and attenuated deviant–standard differences in our click-train dataset, the observed corticofugal effects may depend on the experimental context. In particular, differences in stimulus design, analysis windows, and recording conditions may influence the apparent effect of cortical cooling on MGB responses.

We also agree that the difference from Antunes and Malmierca (2011) requires a cautious interpretation. In our click-train dataset, cortical cooling did not produce a similarly strong and consistent overall reduction in MGB firing rates. However, when we examined responses to noise stimuli in the same experimental preparation, cortical cooling did reduce MGB firing rates, consistent with the previous report. This argues against ineffective cooling as the sole explanation and suggests that the apparent discrepancy may be related to the stimulus condition and analysis context. We have included the corresponding cooling effect on noise-evoked MGB responses below for clarification.

**Commemt Fig 2. Cooling effect on noise-evoked MGB responses in the same experimental preparation.**

We also agree that, if overall firing-rate suppression under cooling is weak or variable for click-train stimuli, then reduced CSI should not automatically be interpreted as evidence for a specific top-down predictive mechanism. In the revised manuscript, we therefore no longer present the cooling effect as supporting a unique mechanistic account. Instead, we state more cautiously that cortical cooling altered MGB oddball/context sensitivity, but that the precise basis of the CSI reduction remains unresolved. As you suggest, this could in principle reflect multiple factors, including altered corticothalamic contextual modulation, changes in adaptation dynamics, or gradual response changes such as habituation or state effects of the kind discussed by Harpaz et al. (2021).

Accordingly, we have substantially narrowed the interpretation of Fig. 6. Our revised conclusion is that the cooling data provide causal evidence that cortical input contributes to MGB sensitivity to click-train temporal patterns, but they do not by themselves determine whether the reduced CSI arises from predictive signaling, general contextual modulation, habituation-related effects, or a combination of these mechanisms.

We are grateful for this comment because it led us to place the cooling results in a more appropriate context relative to prior work and to present their interpretation in a more balanced and cautious way.

**Revised text in the manuscript:**

“Thus, cortical feedback contributes importantly to MGB sensitivity to these click-train temporal patterns, but the present data do not determine the specific circuit mechanism underlying this effect.” (P.21, Lines 7-9)

“However, compared with previous studies of corticofugal modulation of MGB responses, differences in stimulus design, analysis windows, and recording conditions may influence the observed corticofugal effects.” (P.30, Lines 1-4)

“Second, the underlying neuronal circuit mechanisms also remain incompletely resolved. In particular, although cortical cooling provided causal evidence that cortical input contributes to MGB sensitivity under our paradigm, the present data do not determine whether this effect reflects predictive signaling, gain control, adaptation dynamics, changes in inhibitory-excitatory balance, or other forms of context-dependent modulation.” (P.31, Lines 11-16)

P. 19, Fig. 7: For this analysis to be valid, the neuropixel electrodes had to be introduced strictly perpendicular to the surface, so that the neurons from each layer were sampled from the same column - otherwise , if the electrodes were slanted, different layers were sampled at different locations in auditory cortex, and the difference in CSI magnitude could be due to that rather than to the layer composition (this is an old and consistent finding - CSIs are not uniform on the cortical surface; it goes back as far as the initial reports on such effects, e.g. Ulanovsky et al. 2004 in J. Neurosci.). The authors must have the relevant data (histological reconstructions of their Neuropixels tracks).
**Our Response**

We thank you for this important comment. We agree that the validity of the laminar analysis depends critically on accurate verification of probe trajectory and layer assignment. If the Neuropixels probe were substantially slanted relative to the cortical surface, apparent layer-dependent differences in CSI could in principle be confounded by sampling neurons from different horizontal locations in auditory cortex rather than from a comparable cortical column.

In the revised manuscript, we have therefore added histological and physiological documentation of the laminar recordings. Specifically, we now provide a new supplementary figure showing the reconstructed Neuropixels track in auditory cortex, together with the corresponding laminar LFP and current source density profiles evoked by noise stimulation. The histological reconstruction shows that the probe track passed through AC with an approximately radial trajectory in the reconstructed example, and the CSD profile provides an independent physiological basis for assigning recording depth to supragranular, middle, and deep cortical layers.

We have also revised the Methods to clarify how cortical layers were assigned. The laminar classification was based on two criteria: the estimated recording depth relative to published rat auditory-cortex layer depths, and the CSD sink/source profile across the electrode shaft. Based on these criteria, neurons were grouped into supragranular, middle, and deep layers for the Fig. 7 analysis.

At the same time, we agree that laminar conclusions in this type of in vivo preparation should be interpreted cautiously. Although the added histological and CSD evidence supports the validity of the laminar assignment, we cannot completely exclude residual sampling variability across nearby cortical locations. Accordingly, we have tempered the wording of the Results and Discussion. We now describe the finding as layer-associated differences in CSI within the reconstructed A1 recordings, rather than as definitive proof of a strictly columnar laminar gradient independent of all spatial sampling factors.

We are grateful for this comment because it prompted us to strengthen the anatomical and physiological documentation of the laminar analysis and to present the Fig. 7 interpretation more rigorously.

**Revised text in the manuscript:**

“Since AC contributed importantly to sensitivity to click-train temporal patterns, we next asked whether this sensitivity differed across cortical layers. To support laminar assignment, Neuropixels probe tracks were reconstructed histologically, and layer boundaries were further estimated from noise-evoked LFP and current source density (CSD) profiles (S5 Fig.). Based on recording depth and CSD profiles, A1 neurons were grouped into supragranular layers (layers I/II, n = 30), middle layers (layers III/IV, n = 176), and deep layers (layers V/VI, n = 465) for analysis.” (P.21, Line 24- P.22, Line5)

“For laminar recordings in A1, Neuropixels probes were inserted as close as possible to perpendicular to the cortical surface. After recording, probe tracks were reconstructed histologically to verify the trajectory through auditory cortex. Layer assignment was based on two complementary criteria: recording depth along the probe relative to published rat auditory-cortex layer depths, and the noise-evoked CSD profile calculated from LFP responses (example shown in S5 Fig). Based on the combination of anatomical depth and CSD sink/source patterns, neurons were assigned to supragranular, middle, or deep cortical compartments.” (P.48, Lines 4-11)

“S5 Fig. Histological reconstruction and laminar CSD profile of a Neuropixels penetration in A1. (A) Histological reconstruction of the Neuropixels probe track in auditory cortex. The orange signal marks the probe track within AC, and the dashed line indicates the estimated trajectory of the probe. Scale bar, 1 mm. (B) Laminar LFP and current source density profiles evoked by noise stimulation from the same penetration. Left, LFP responses recorded along the probe from superficial to deep cortical depths. Middle, schematic of the 384-channel Neuropixels probe, with channels selected every 80 µm for CSD analysis. Right, CSD profile calculated from the selected LFP channels and smoothed for visualization. Sources are shown in blue and sinks in red. Horizontal dashed lines indicate approximate boundaries between cortical layers. Vertical dashed lines indicate noise onset.” (P.56, Lines 1-11)

Discussion: There are some fundamental errors of fact and interpretation here. Temporal structure at the ms range is encoded both temporally and spectrally. For example, a click train with a 4 ms interval has an extremely strong pitch at 250 Hz, and such click trains and their modifications have been studied in great details as part of studies of pitch. The authors ignore this literature completely. Thus, the novelty of the current study is certainly not in using short-interval click trains as auditory stimuli. Also, the ability of listeners to discriminate between such sequences, as shown here in Fig. 2, is not in itself surprising - different stimuli evoke different responses, and these click trains evoke very distinctive response patterns in the auditory nerve array. Thus, the 'new feature of sound' (P. 21) aspect of this study is likely over-played. Similarly, in the paragraph spanning P. 21 and 22, '…click trains offer a particularly useful approach, because they allow
temporal configuration to be manipulated while largely controlling other acoustic dimensions.' Is wrong, because of the spectral features that are inherent in the use of such click trains (with intervals that are shorter than the typical ringing time of the basilar membrane, creating spectral features in the responses). Just the fact that you are capable of manipulating easily the intervals doesn't mean that you have an independent perceptual feature. This comment is pertinent to further places in the discussion where the authors discuss the 'purely temporal' manipulations they presumably used.

**Our Response**

We thank you for this important comment. We agree that the Discussion in the previous version contained overstatements and did not adequately acknowledge the established literature showing that millisecond-range click-train structure has both temporal and spectral consequences, including strong pitch-related and broader spectrotemporal representations. We also agree that the novelty of the present study does not lie in the mere use of short-interval click trains as auditory stimuli, nor in demonstrating that such stimuli can in principle be discriminated.

In the revised manuscript, we have therefore substantially rewritten the Discussion to correct these issues. First, we no longer describe the studied phenomenon as a “new feature of sound,” nor do we claim that our stimuli isolate a purely temporal auditory dimension. Instead, we now frame the study more cautiously as examining sensitivity to different click-train temporal patterns, while explicitly acknowledging that these manipulations necessarily also generate spectral and spectrotemporal structure, including pitch-related consequences for some stimulus classes.

Second, we have revised the Discussion to place our work more appropriately in the context of prior literature on short-interval click trains, pitch, and fast temporal-pattern processing. We now make clear that the present study is not intended to introduce short-interval click trains as a novel stimulus class, but rather to use them as a tool for systematically probing how different click-train temporal patterns, together with their associated spectrotemporal consequences, are represented across multiple levels of the auditory system.

Third, we have removed or rewritten statements implying that click trains allow temporal structure to be manipulated while largely controlling other acoustic dimensions in a strict sense. We agree that this wording was inaccurate. In the revised manuscript, we now state explicitly that although inter-click timing is the manipulated variable, nearby clicks interact within auditory processing and therefore also shape spectral and broader spectrotemporal representations. Accordingly, the present findings should not be interpreted as evidence for an independent purely temporal perceptual feature.

More broadly, we have revised the novelty claim of the paper to better reflect what the data actually support. In the revised Discussion, we now emphasize that the contribution of this study lies in the systematic and multi-level characterization of sensitivity to different click-train temporal patterns across human and rat auditory systems, the hierarchical differences observed along the auditory pathway, and the causal contribution of corticothalamic feedback, rather than in claiming the discovery of a fundamentally new acoustic feature.

We are grateful for this comment because it prompted a major improvement in the factual accuracy and conceptual balance of the Discussion.

**Revised text in the manuscript:**

“Accordingly, the stimuli differed in their inter-click timing patterns and, as a consequence, also in their associated short-term spectrotemporal representations.” (P.7, Lines 19-20)

“In this context, click trains offer a particularly useful approach because they allow millisecond-scale temporal patterns to be manipulated systematically, even though such manipulations also give rise to associated short-term spectrotemporal consequences.” (P.24, Lines 17-20)

“These findings indicate that distinct click-train temporal patterns, together with their associated spectrotemporal consequences, are differentially represented across the auditory system, with stronger sensitivity emerging in higher auditory structures. However, the present data do not establish that these stimuli isolate a purely temporal auditory dimension, nor do they identify the exact acoustic or temporal statistics encoded by the underlying neural circuits.” (P.23, Line 21-P.24, Line 3)

“A key open question is which acoustic or temporal statistics drive the observed behavioral discrimination. Although the present study shows that discrimination cannot be explained simply by mean inter-click interval or by long-term spectral matching, it does not identify the specific computational variable extracted by the auditory system. Several candidate accounts remain possible, including temporal regularity-related cues, pitch-like perceptual consequences of fast click trains, modulation-based representations, asymmetric temporal filtering/integration, or other higher-order temporal descriptors. Because the current stimulus set was not designed to isolate these alternatives parametrically, the present data do not allow a decisive distinction among them.” (P.25, Line 17-P.26, Line 4)

“This broader interpretation is consistent with previous work showing that fast click trains can generate pronounced pitch-related and spectrotemporal effects, and that closely spaced clicks may interact monaurally and spectrally in related perceptual paradigms.” (P.26, Lines 14-17)

Minor comments

Top of P. 7, description of the stimuli: the nature of the individual event used as click is undefined. Was it a single sample of the digital waveform? If so, what was the sampling rate used?
**Our Response**

We thank you for pointing out this omission. You are correct that the previous description of the stimuli did not define the physical nature of the individual click event clearly enough.

In the revised manuscript, we have now clarified that each click was not a single-sample digital impulse, but a brief pulse with a duration of 200 µs. We have also specified the sampling rates used for stimulus generation and playback in the human EEG and animal experiments. Specifically, for human EEG recordings, the click sounds were synthesized at 384 kHz with 32-bit precision, whereas for the animal ECoG and extracellular-recording experiments, the acoustic stimuli were digitally generated at 97,656 Hz using the TDT system. We have added this information to the stimulus description in the Methods so that the physical definition and temporal precision of the click events are fully transparent.

We are grateful for this comment because it helped us improve the completeness and reproducibility of the stimulus description.

**Revised text in the manuscript:**

“We developed three experimental procedures for EEG, ECoG, and extracellular recording, employing ten unique click trains as depicted in S1 Table. Each click train lasted 200 ms, consisting of 51 fixed pulses, with each pulse lasting 200 µs and an average inter-click interval (ICI) of 4 ms.” (P.37, Lines 4-7)

“For human EEG recordings, click sounds were synthesized using MATLAB R2022b (The MathWorks, Inc., Natick, MA, USA) on a computer equipped with a high-fidelity sound card (Creative, AE-7). These clicks were produced with a sampling rate of 384 kHz and 32-bit precision and then broadcast through a stereo speaker system (Golden Field M23), ensuring the precise presentation of click train sequences. In contrast, animal ECoG and Extracellular recording experiments were conducted within a sound-proof room, with stimuli played contralaterally to the recording site in rats. These acoustic stimuli were digitally generated using a computer-controlled Auditory Workstation (RZ6, TDT) at a sampling rate of 97656 Hz and delivered through magnetic speakers (MF1, TDT) within the TDT systems.” (P.37, Lines 12-21)

P. 8, success rate in the 'easy' conditions is reported with too many significant digits (the error bars are much larger than 0.001%). Two significant digits would be sufficient.

**Our Response**

We thank you for this helpful comment. We agree that the behavioral success rates in the easy conditions were reported with unnecessary numerical precision in the previous version.

In the revised manuscript, we have rounded the reported accuracy values to two decimal places, which better reflects the precision of the behavioral measurements and the variability across participants. We have also retained the individual participant values in Fig. 1C to show the distribution of responses across participants.

We are grateful for this suggestion because it helped us present the behavioral data more appropriately.

**Revised text in the manuscript:**

“In contrast, the correct response rates for the Con-Asc, Asc-TW30, Asc-Dec, RN60-TW10 and RN60-TW70 pairs were 0.97, 0.96, 0.92, 0.92 and 0.91, respectively (Fig 1C), all significantly greater than the reference proportion of 0.65 (p < 0.01 for all five pairs, Kolmogorov–Smirnov test), indicating that participants recognized the two temporally merged auditory objects as distinct entities.” (P.9, Lines 9-14)

P. 14, middle paragraph: The A1 neuron in Fig. 4F showed similar responses to standards and deviants, with slight advantage to deviants. It shouldn't be described as having 'robust responses to deviants' without reference to its robust responses to standards.

**Our Response**

We thank you for this accurate observation. We agree that the previous wording overemphasized the deviant response and did not adequately acknowledge that the example A1 neuron also showed robust responses to the standard condition.

In the revised manuscript, we have corrected this description to reflect the data more accurately. Rather than describing the neuron as showing “robust responses to deviants,” we now state that the neuron showed robust responses in both the standard and deviant conditions, with the deviant response being clearly stronger. This revision better matches the actual response pattern and avoids overstating the selectivity of the example neuron.

We are grateful for this comment because it helped us improve the accuracy and balance of the description of the single-neuron example.

**Revised text in the manuscript:**

“In the pair RN10 and TW50, the A1 neuron showed robust responses in both the standard and deviant conditions, with the deviant response being clearly stronger (raster plots in red, Fig 4D), and significant differences between deviant and standard responses were observed for both trains.” (P.15, Lines 22-25)

Fig. 5, lower (color) panels of A-C and on: I found them confusing. Maybe show scatter plots of the spike counts that serve for the t-tests reported in the text, rather than the time-resolved firing rate differences which are affected by many stimulus-dependent and independent factors.

**Our Response**

We thank you for this helpful suggestion. We agree that the lower heatmap panels in Fig. 5 can be difficult to interpret, especially because time-resolved firing-rate differences may reflect a mixture of stimulus-specific and non-stimulus-specific factors.

At the same time, our intention in retaining these panels was to show the temporal evolution of the deviant–standard differences across the neuronal population, rather than only the summary spike-count comparison underlying the statistical tests. We believe that these plots remain useful for visualizing when in the response window the population differences emerge and how they vary across auditory levels and stimulus pairs.

In the revised manuscript, we have therefore improved the presentation of these panels rather than removing them. Specifically, we have revised the heatmaps to use a diverging colormap centered at 0, so that positive and negative differences can be distinguished more clearly. We have also clarified the figure legend to state explicitly that these panels show z-scored deviant–standard PSTH differences across neurons. The statistical tests reported in the text remain based on the corresponding response measures described in the Methods.

We are grateful for this suggestion because it helped us improve the clarity and interpretability of Fig. 5.

**Revised text in the manuscript:**

“Bottom panels: Heatmaps illustrating the z-scored differences (deviant − standard) in PSTHs of firing rates for each neuron across the population. A diverging colormap centered at 0 was used, with warmer colors indicating positive differences and cooler colors indicating negative differences.” (Fig. 5 legend)

P. 16, last few words are corrupted. Similarly Middle of the top paragraph of P. 17, (S2 Fig.) is reversed in many references to the supp info figure. This is so also in other places in the manuscript.
**Our Response**

We thank you for noticing these textual and cross-referencing errors. In the revised manuscript, we have corrected the corrupted text at the end of p. 16 and systematically checked and corrected the references to S2 Fig. and other supplementary figures throughout the manuscript. We are grateful for this comment because it helped us improve the clarity and editorial quality of the manuscript.

**Revised text in the manuscript:**

“The results from the subdivisions of MGB and IC are summarized in S1 Fig.” (P.18, Line 10)

“including neurons from A1 (n = 94), MGB (n = 109), and IC (n = 140) (S2 Fig).” (P.18, Line 16)

Methods, P. 32, complex tones: These are not 'harmonic' - harmonic complexes have frequencies that are, well, harmonics of a fundamental (so equal differences in frequency). The tone complexes used here had components that were equally spaced on a logarithmic scale (equal ratios).

**Our Response**

We thank you for this correction. We agree that our use of the term “harmonic” in this context was inaccurate. As you correctly point out, a harmonic complex should consist of frequency components that are integer multiples of a fundamental, whereas the stimuli used here were constructed with components equally spaced on a logarithmic frequency scale, that is, equal frequency ratios.

In the revised manuscript, we have corrected this terminology throughout the Methods and elsewhere in the text where relevant. We now refer to these stimuli more accurately as logarithmically spaced tone complexes, rather than harmonic complexes.

We are grateful for this comment because it helped us improve the technical accuracy of the stimulus description.

**Revised text in the manuscript:**

“To complement the findings obtained from click train pairs, we designed an oddball sequence using two logarithmically spaced tone complexes. One tone complex comprised frequency components spaced at 1/8-octave intervals, beginning at 600 Hz. The component frequencies (e.g., 654, 712, 777, …, 39,910 Hz) followed this logarithmic progression. The other tone complex began at 680 Hz, also with 1/8-octave spacing between adjacent components. Thus, the two complex tones did not share similar frequencies.” (P.39, Lines 9-15)

Reviewer #3: Reviewer comments:

This study investigates whether listeners can discriminate distinct patterns of rapidly presented temporal clicks and the neural mechanisms that may underlie such discrimination. The authors first show that listeners can discriminate click patterns with the same average inter-click interval (ICI) even when presented at a rapid rate such that the clicks fuse into a single percept. They then show that temporal pattern deviants produce an MMN in human EEG recordings, as well as in ECoG recordings in rat auditory cortex. They use an SSA paradigm to investigate these responses via extracellular recording in the inferior colliculus, medial geniculate, and auditory cortex. They use laminar probes to investigate layer-wise organization in the cortex and cortical cooling to investigate the impact of top-down cortico-thalamic effects.
Overall, this is a nice study. The question being addressed is novel and significant. The methods are generally high quality. The paper is clearly written and easy to read.
**Our Response**

We thank you for this positive and encouraging assessment of our work. We are pleased that you found the study question novel and significant, the methods generally high quality, and the manuscript clear and easy to read.

We also appreciate your concise summary of the study and its multi-level approach, spanning human behavior, human EEG, rat ECoG, single-unit recordings, laminar analysis, and cortical cooling. We are grateful for your encouraging evaluation and have carefully considered the specific comments below to improve the manuscript further.

Major comments & critiques:

As far as I can tell, it is not possible to listen to the sounds. I, for example, could not find a link that would enable me to listen to the different click trains presented in the study. I do not think it is possible to thoroughly review this manuscript without being able to listen to the sounds.

**Our Response**

We thank you for this important comment. We agree that access to the stimulus sounds is very helpful for evaluating the manuscript, especially for a study centered on perceptual and neural responses to different click-train patterns.

In the revised manuscript, we have addressed this issue by making the stimulus sounds available to readers. Specifically, all audio files corresponding to the click-train stimuli are now provided in the Supporting Information. We have also added an explicit statement in the main text to guide readers to these files.

We are grateful for this suggestion because it improves the transparency, accessibility, and evaluability of the work.

**Revised text in the manuscript:**

“All audio files and corresponding spectral analyses of the click-train stimuli are provided in the Supporting Information (S1 File).” (P.7, Lines 20-22)

Discrimination performance cannot be explained by the mean inter-click interval or the spectrum, since these are the same. But there is nothing in the study that addresses what acoustic statistics drive listeners' performance. Can the results be explained by a measure of temporal regularity related to pitch perception? Could a temporal modulation filterbank with asymmetric temporal tuning (e.g., fast onset, slow offset) explain this? Being able to listen to the sounds would be very helpful in generating hypotheses.

**Our Response**

We thank you for this important and insightful comment. We agree that, although the present study shows that discrimination cannot be explained simply by mean inter-click interval or long-term spectral magnitude alone, it does not yet determine which more specific acoustic or temporal statistics drive listeners’ performance. We also agree that several plausible candidate mechanisms remain open, including measures related to temporal regularity, pitch-related processing, temporal modulation analysis, or asymmetric temporal integration of the kind you suggest.

In the revised manuscript, we have therefore substantially clarified the scope of our claims. Specifically, we no longer imply that the present data identify the exact acoustic statistic or computational operation underlying behavioral discrimination. Instead, we now state more cautiously that the results demonstrate sensitivity to differences in click-train temporal patterns, while leaving open which specific descriptors or neural computations best account for that sensitivity.

We have also revised the Discussion to address this issue explicitly. In particular, we now note that the observed behavioral differences could in principle arise from multiple candidate representations, including temporal regularity-related cues, pitch-like perceptual consequences of fast click trains, modulation-based representations, asymmetric temporal filtering or integration, or other higher-order temporal descriptors. We further clarify that the present dataset was not designed to distinguish decisively among these alternatives.

In addition, as you suggested, we have made the stimulus sounds available in the revised manuscript so that readers can listen to the click trains directly. Specifically, all audio files corresponding to the click-train stimuli are now provided in the Supporting Information.

More broadly, we now explicitly state that identifying the statistics that best explain behavioral performance will require future work using more formal modeling and targeted stimulus designs, for example by comparing alternative temporal-regularity, pitch-related, and modulation-based accounts within a more parametric framework.

We are grateful for this comment because it helped us sharpen an important limitation of the current study and improve the Discussion accordingly.

**Revised text in the manuscript:**

“All audio files and corresponding spectral analyses of the click-train stimuli are provided in the Supporting Information (S1 File).” (P.7, Lines 20-22)

“However, the present behavioral data do not determine which specific acoustic or temporal descriptors best account for this discrimination.” (P.10, Lines 1-2)

“A key open question is which acoustic or temporal statistics drive the observed behavioral discrimination. Although the present study shows that discrimination cannot be explained simply by mean inter-click interval or by long-term spectral matching, it does not identify the specific computational variable extracted by the auditory system. Several candidate accounts remain possible, including temporal regularity-related cues, pitch-like perceptual consequences of fast click trains, modulation-based representations, asymmetric temporal filtering/integration, or other higher-order temporal descriptors. Because the current stimulus set was not designed to isolate these alternatives parametrically, the present data do not allow a decisive distinction among them.” (P.25 Line 17 –P.26, Line4)

All of the neural sensitivity measures are based on deviance/SSA, which seems odd to me. The most basic question one might ask is whether the neural populations being studied can discriminate the sounds tested, analogous to their initial behavioral study. It's interesting to examine SSA, but it feels to me like the authors are missing a step.

**Our Response**

We thank you for this insightful comment. We agree that the manuscript moved too quickly from the behavioral demonstration of perceptual discrimination to deviance/SSA-based neural analyses, without making sufficiently explicit the more basic question of whether the recorded neural populations can, in principle, distinguish the tested click-train patterns.

In the revised manuscript, we have addressed this issue by clarifying the logic of our neural analyses. Our original motivation for using oddball/SSA paradigms was that they provide a well-established framework for testing whether differences among click-train patterns are reflected in context-dependent neural responses, and for comparing such sensitivity across auditory levels in relation to deviance detection. However, we agree with you that this does not fully substitute for the more basic question of neural discriminability.

Accordingly, we have revised the manuscript to make this limitation explicit and to avoid implying that SSA-based sensitivity is equivalent to a complete neural discrimination analysis. We now state more clearly that the present neural results demonstrate context-dependent neural sensitivity to click-train patterns under oddball paradigms, but do not by themselves provide a full population-decoding account analogous to the behavioral discrimination experiment. We also now emphasize in the Discussion that a more direct neural analogue of the behavioral task would require explicit decoding or representational analyses testing how well neural populations can distinguish the click-train patterns independently of the oddball framework.

We are grateful for this comment because it helped us clarify the distinction between context-dependent deviance sensitivity and basic neural discriminability, and it has improved the conceptual framing of the neural analysis in the revised manuscript.

**Revised text in the manuscript:**

“To examine whether these perceptually discriminable click-train patterns were also differentially represented in neural activity, we next used oddball paradigms to test whether changes in click-train pattern elicited context-dependent neural response differences across multiple levels of the auditory system.” (P.10, Lines 5-8)

“More broadly, several observed behavioral–neural dissociations indicate that neural sensitivity to temporal-pattern differences may exceed what is accessible under a particular behavioral task, and the current study does not provide a formal model linking perceptual discrimination, population coding, and deviance-related responses across levels of the auditory pathway.” (P.31, Line 20-P.32, Line 3)

“Addressing these issues will require future work using more controlled parametric stimulus designs, formal model comparison, direct neural decoding analyses, and more specific circuit manipulations.” (P.32, Lines 3-5)

The behavioral forced choice accuracy is a little hard to interpret because the reported measures are dependent on listeners' criterion (explaining below-chance accuracy). It would be useful to have a criterion-free measure of sensitivity, which could be compared with their neural data.

**Our Response**

We thank you for this important comment. We agree that raw forced-choice accuracy can be difficult to interpret when response bias or decision criterion may influence performance, especially in conditions where accuracy approaches or falls below the nominal reference level. We also agree that a criterion-free sensitivity measure provides a clearer basis for interpreting the behavioral data and for relating them more appropriately to the neural findings.

In the revised manuscript, we have addressed this issue by adding a criterion-free sensitivity analysis using d′, which is now shown in a new panel of Fig. 1D. This analysis provides a criterion-free measure of perceptual sensitivity and offers a clearer summary of sensitivity across both difficult and easy stimulus pairs. We have also revised the Results, Methods, and figure legend to explain how d′ was calculated and how it should be interpreted.

We are grateful for this suggestion because it improves both the clarity of the behavioral analysis and its connection to the neural findings.

Revised text in the manuscript:

“To provide a criterion-free measure of behavioral sensitivity, we calculated d′ values for each stimulus pair (Fig 1D). This analysis showed a graded pattern of sensitivity across stimulus pairs, with lower d′ values for difficult pairs such as TW10–TW30, Con–RN10, and RN10–TW10, and higher d′ values for more easily discriminable pairs such as Con–Asc, Asc–TW30, Asc–Dec, RN60–TW10, and RN60–TW70” (P.9, Lines 15-20)

“To provide a criterion-free measure of behavioral sensitivity, we calculated d′ for each stimulus pair using signal detection theory. For each different-stimulus pair, the hit rate was defined as the proportion of trials in which participants responded “different” to that pair. The false-alarm rate was defined as the proportion of trials in which participants responded “different” to identical-pair trials. Because multiple identical-pair conditions were included, the false-alarm rate was estimated using responses pooled across all identical-pair trials. To avoid infinite d′ values, hit and false-alarm rates of 0 or 1 were corrected using the standard log-linear correction. d′ was then calculated as z (hit rate) − z (false-alarm rate). A global d′ value was also calculated by pooling responses across all different-stimulus trials and all identical-stimulus trials.” (P.33, Lines 10-19)

“(D) d′ values calculated from the behavioral responses shown in (C). For each different-stimulus pair, d′ was calculated from the hit rate and false-alarm rate derived from the same/different responses. The hit rate was defined as the proportion of “different” responses for that stimulus pair, whereas the false-alarm rate was estimated from responses to identical-pair trials. For the identical-pair reference, all identical control conditions were included. The “Global” category summarizes d′ values calculated by aggregating responses across all different-stimulus trials and all identical-stimulus trials. Box plots show the distribution of d′ values across participants, with the central line indicating the mean, box edges representing the interquartile range, and whiskers showing the full range. Orange points denote individual participant scores. The horizontal dashed line at d′ = 0 indicates chance-level sensitivity.” (Legend of Fig.1D)

The analysis where they select units in IC that show SSA for harmonic tones is nice. This analysis should be cross-validated, however, to make sure they are not just picking up on noise. Please use a subset of data to select the units and then make sure they still show enhanced SSA for harmonic tones in left out data.

**Our Response**

We thank you for this valuable suggestion. We agree that the selection analysis for IC units showing strong SSA to the complex-tone stimuli should be cross-validated to ensure that the observed effect is not driven by noise or by circular selection on the same dataset used for evaluation.

In the revised manuscript, we have addressed this point by implementing a split-half cross-validation procedure. Specifically, we now use one subset of the data to identify IC units with significantly positive CSI for the complex-tone stimuli, and then test whether these same units continue to show positive SSA in an independent held-out subset of trials. This procedure allows us to verify that the effect is reproducible and not simply a consequence of selection bias.

We have added the corresponding description to the Methods and revised the Results text accordingly to report the cross-validated outcome. More generally, we now make clear that the purpose of this analysis is to identify a CT-SSA-positive subpopulation in a conservative manner, rather than to infer population-wide properties from a potentially circular selection procedure.

We are grateful for this comment because it has strengthened the rigor of this analysis and improved confidence in the interpretation of the selected IC subpopulation.

**Revised text in the manuscript:**

“To reduce possible selection bias, we further performed a split-half cross-validation analysis, in which neurons were selected using one subset of the CT trials based on significantly positive CSI, and CSI was then recomputed for these neurons using an independent held-out subset of trials. The selected neurons continued to show positive CT-evoked SSA in the held-out data, indicating that this control effect was not attributable solely to circular selection or noise.” (P.18, Line 18-P.19, Line 1)

“To further reduce possible circular-selection bias, we performed a split-half cross-validation analysis on the complex-tone trials. For each neuron, trials were randomly divided into two independent subsets. Neurons were first selected using one subset of the trials based on significantly positive CSI, applying the same bootstrap criterion described above. CSI was then recomputed for these selected neurons using the independent held-out subset of trials. This procedure allowed us to test whether the positive complex-tone SSA effect remained present in data not used for selection.” (P.44, Lines 13-20)

I think one possible explanation of their findings is that SSA is more general in the cortex, i.e., neurons show adaptation for a wider range of sounds. I don't feel that the authors need to rule out this explanation, but I think it should be explicitly acknowledged in the Discussion.

**Our Response**

We thank you for this thoughtful comment. We agree that a broader or more generalized form of SSA in cortex is a plausible interpretation of the present results and should be acknowledged explicitly.

In the revised Discussion, we now state more clearly that the stronger oddball/SSA-related effects observed in cortex do not necessarily imply a mechanism uniquely specialized for the particular click-train patterns used here. Instead, an alternative and fully plausible explanation is that cortical neurons exhibit adaptation or context sensitivity over a broader range of sounds than subcortical neurons, such that the enhanced effects observed in cortex reflect a more general expansion of SSA-like processing at higher auditory levels.

We also clarify that the present data do not distinguish decisively between specifically enhanced sensitivity to these click-train temporal patterns and a more general increase in cortical adaptation or context sensitivity across diverse stimulus classes. Accordingly, we present the cortical results more cautiously and discuss this broader cortical-SSA interpretation as an important alternative account.

We are grateful for this suggestion because it improves the balance of the Discussion and helps us avoid overinterpreting the cortical findings.

**Revised text in the manuscript:**

“An alternative interpretation is that SSA is more broadly expressed in cortex than in subcortical structures, such that the stronger cortical effects observed here may reflect a more general expansion of adaptation/context sensitivity across a wider range of sounds, rather than a mechanism uniquely specialized for the click-train patterns used in this study. The present data do not distinguish decisively between these possibilities, and the cortical findings should therefore be interpreted with appropriate caution.” (P.28, Line 18-P.29, Line 2)

As far as I can tell, there is no mention of what nucleus they are recording from in the IC or MGB. Prior SSA studies, I believe, have shown big differences between nuclei, so this seems important to engage with.
**Our Response**

We thank you for this important comment. We agree that specifying the recording sites within the inferior colliculus (IC) and medial geniculate body (MGB) is important, given prior studies showing substantial functional differences between subnuclei in these structures.

In the revised manuscript, we have clarified the anatomical location of the recorded neurons. Specifically, we now describe the IC subregions and MGB subdivisions represented in our recordings, based on stereotaxic coordinates, electrode depth, and histological verification where available. We also now provide a subdivision-level summary in S1 Fig. and interpret the population-level results with appropriate caution in light of possible subnuclear variability.

Accordingly, we have revised the manuscript to indicate the relevant anatomical subdivisions where appropriate and to improve the anatomical specificity and interpretive rigor of our IC and MGB analyses.

We are grateful for this comment because it allowed us to improve the anatomical specificity and interpretive rigor of our IC and MGB analyses.

**Revised text in the manuscript:**

“The results from the subdivisions of MGB and IC are summarized in S1 Fig.” (P.18, Line 10)

“S1 Fig. Populational neuronal response to oddball paradigm with temporally merged click-train in A1 and subregions of IC, MGB. (A) Distribution of CSI in A1 (n = 671), MGD (n = 398), MGV (n = 225), EIC (n = 524) and CIC (n = 414) in the oddball paradigm (RN_10_, TW_50_). Arrows indicate the mean. Red bars highlight neurons with CSI significant greater than 0, determined by a nonparametric bootstrap procedure with 1,000 iterations (p < 0.05). **(B-C)** Distribution of CSI values to additional two sound pairs in the oddball paradigm (RN60, Con) and (Asc, TW_30_).” (S1 Fig. legend)

“MGB recordings included neurons located mainly in the ventral and dorsal subdivisions of MGB, whereas IC recordings included neurons located mainly in the central and external subdivisions of IC, as verified by stereotaxic coordinates, electrode depth, and histological reconstruction of probe tracks.” (P.43 Lines 19-22)

It is not always clear what the sample dimension is over which they are computing statistics. For example, if you are bootstrapping a statistic, it needs to be clear what you are bootstrapping. The same goes for a t-test.
**Our Response**

We thank you for this important comment. We agree that the previous version of the manuscript did not always make it sufficiently clear what the sampling unit was for each statistical analysis, including bootstrap procedures and t-tests. This lack of clarity could make it difficult to evaluate exactly what level of variability was being quantified.

In the revised manuscript, we have revised the Methods, figure legends, and relevant Results text to better specify the sample dimension used in the main analyses. In particular, we now clarify whether analyses were performed across channels, subjects, pooled ECoG channels, or neurons, and we specify the response windows used for these comparisons.

For bootstrap analyses of CSI, we now define that significance was assessed at the single-unit level using a nonparametric bootstrap procedure with 1,000 iterations to generate a null distribution of CSI values. We also clarify that units were considered to have significantly positive CSI when the lower bound of the 95% confidence interval exceeded zero. For t-tests and related comparisons, we now specify the relevant observation level, such as channels for individual EEG/ECoG analyses, subjects for EEG population analysis, and neurons for extracellular population comparisons.

We are grateful for this comment because it helped us improve the rigor, transparency, and interpretability of the statistical reporting throughout the manuscript.

**Revised text in the manuscript:**

“For individual subjects, we selected the 0-300 ms window relative to the onset of both deviant and standard stimuli as the calculation window. A grand-average ERP waveform was then obtained by averaging data across selected interesting channels from each subject (refer to Fig 2D). These channels were situated on the temporal, parietal, and occipital lobes. In total, 27 channels were utilized in following processing. Furthermore, we computed the mean amplitude of the average response from each channel. For this computation, we applied a sliding window approach with a window size and step size of 5 ms. Subsequently, an independent-samples t-test with FDR correction was conducted to determine if there was a significant difference between the mean values of the deviant and standard conditions across channels.” (P.45 Lines 6-15)

“For population analysis, we averaged the grand-average waveform across all subjects (refer to Fig 2E-2H). We also calculated the mean value on every grand-averaged waveform using the same sliding window parameters and conducted an independent-samples t-test with FDR correction to assess the differences between the deviant and standard stimulus conditions across subjects.” (P.45 Lines 16-20)

“For population-level analysis, we calculated the mean amplitude of the ECoG waveforms within a 0–200 ms window following the onset of each deviant stimulus and compared it with the mean amplitude during a 50 ms baseline window preceding the stimulus. Channels from each rat where the response exceeded 2 standard deviations above the baseline were selected and pooled together (N = 3 rats, n = 36 channels). These pooled channels were averaged to create a population-level waveform.” (P.46 Lines 7-12)

“Meanwhile, to assess the significance of the CSI for each unit, we employed a nonparametric bootstrap procedure with 1,000 iterations to generate a null distribution of the CSI values. The 95% confidence interval (CI) of the bootstrapped distribution was then computed. A unit was considered to have a significantly greater-than-zero CSI if the lower bound of the 95% CI exceeded zero.” (P.47 Lines 6-10)

“Following spike count computation, the window was consistently chosen from 5 to 200 ms relative to the onset of the deviant stimuli or the ninth standard stimuli. It should be noted that all data analyzed using ANOVA or t-test in this study were assessed for normality using the Shapiro-Wilk test prior to analysis. Otherwise, the Wilcoxon signed-rank test or Mann-Whitney U test was used.” (P.47 Lines 13-17)

Minor notes:

When plotting images where 0 is meaningful (e.g., Figure 5), the authors should use a diverging colormap that makes it easy to see when values are below or above 0 (e.g., blue(-1):white(0):red(1)).

**Our Response**

We thank you for this helpful suggestion. We agree that, for plots in which 0 is a meaningful reference value, a diverging colormap is more appropriate and improves interpretability.

In the revised manuscript, we have updated the relevant heatmap panels in Fig. 5 to use a diverging colormap centered at 0, so that positive and negative values can be distinguished more clearly. We have also revised the corresponding figure legend to clarify this presentation.

We are grateful for this suggestion because it improves the clarity and visual interpretability of the figure.

**Revised text in the manuscript:**

“Bottom panels: Heatmaps illustrating the z-scored differences (deviant - standard) in PSTHs of firing rates for each neuron across the population. A diverging colormap centered at 0 was used, with warmer colors indicating positive differences and cooler colors indicating negative differences.” (Fig. 5 legend)

I think the authors should give an example of TW Figure 1, as they do for other stimuli. Please also define the acronym at first usage.

**Our Response**

We thank you for this helpful suggestion. We agree that the TW stimulus class should be illustrated explicitly in Fig. 1, just as for the other stimulus types, and that the acronym should be defined at first use.

In the revised manuscript, we have therefore added an example of the TW stimulus to Fig. 1A and defined TW at its first appearance as two-way, indicating that the inter-click interval was randomly selected from two fixed values around the mean ICI. These changes make the stimulus set easier to understand and improve the consistency of the presentation across stimulus classes.

We are grateful for this comment because it helped us improve the clarity of the stimulus description for readers.

**Revised text in the manuscript:**

“TW10 (two-way, 10%) consisted of click trains in which each ICI was randomly selected as either 3.6 ms or 4.4 ms” (P.7, Lines 5-6)

“the TW stimuli represented two-way interval patterns” (P.7, Lines 12)

“The Asc and Dec stimuli therefore represented ordered monotonic interval patterns, the TW stimuli represented two-way interval patterns, and the RN stimuli represented random interval patterns with different degrees of spread.” (P.7, Lines 11-13)
