# Supplementary material for: Distinct millisecond-scale click-train patterns evoke discriminable behavioral and neural responses in humans and rats
Source: PLoS Biol. 2026 Oct 8;24(10):e3004046. doi: 10.1371/journal.pbio.3004046 (PMC13686464; doi:10.1371/journal.pbio.3004046)
Supplement: Supplementary file 3 [file pbio.3004046.s012.docx]

REVIEWS:

Reviewer #1: Thank you for the substantial effort invested in revising the manuscript. I have carefully read the revised manuscript, response letter, figures, and supporting information.

In my previous review, my main concern was that the manuscript made broad conceptual claims regarding feature-like encoding of millisecond-scale temporal configurations, hierarchical computation, and cross-species conservation without providing a sufficiently explicit stimulus-to-neural mapping or mechanistic account. I felt that supporting those claims in their original form would require substantial additional experiments, parametric analyses, and modeling.

The revisions address these concerns in a thoughtful and constructive manner. While the current manuscript still does not provide the missing parametric or encoding-model analyses, nor does it identify the specific acoustic or temporal statistics encoded by the auditory system, the authors have substantially narrowed the scope of their claims so that the central conclusions no longer depend on these stronger mechanistic interpretations. In particular, the manuscript no longer presents the findings as establishing a novel, purely temporal auditory feature, and it now acknowledges that the click-train manipulations necessarily produce spectrotemporal consequences. The stimulus set is also more appropriately described as an operationally defined family of click-train patterns rather than as samples drawn from a formally parameterized temporal feature space.

Several specific revisions further strengthen the study. The inclusion of a spectrally matched control is valuable, although it remains important to recognize that matching long-term spectral magnitude does not guarantee equivalent peripheral or neurogram-level representations. The revised ECoG analysis now employs a more appropriate comparison between responses to the same stimulus presented as standard versus deviant. The behavioral analyses have been corrected and supplemented with d′ measures. The IC selection analysis is now cross-validated, relevant subnuclei are specified, and the interpretation of the cortical-cooling experiment has been appropriately moderated so that predictive coding is no longer presented as the sole possible explanation.
Overall, the concerns regarding overstatement that motivated my original recommendation have largely been resolved. The manuscript is now more accurately framed as a systems-level characterization of behavioral and neural sensitivity to rapid click-train patterns, rather than as a mechanistic demonstration of a novel auditory feature.

**Our Response:**

We sincerely thank you for your careful reassessment of the revised manuscript and for recognizing the substantial changes made in response to the previous review. We appreciate your assessment that the manuscript is now scientifically sound and more appropriately framed as a systems-level characterization of behavioral and neural sensitivity to rapid click-train patterns.

We agree with both of the two remaining points. First, CSI/SSA measures do not by themselves distinguish use-dependent adaptation from genuine deviance sensitivity or prediction-error signaling. Second, the present work should be positioned as an extension of established findings on hierarchical stimulus-specific adaptation to rapid click-train patterns, rather than as a fundamentally new conceptual advance. Accordingly, we have further revised the Discussion to make both points explicit.

Two limitations regarding the broader conceptual impact remain.

First, the principal neural findings continue to rely on CSI/SSA measures, namely comparisons between responses to the same stimulus when presented as a deviant versus as a standard, across IC, MGB, and A1. Such measures alone cannot distinguish genuine deviance sensitivity or prediction-error signaling from use-dependent adaptation, for example differential adaptation of narrowly tuned feedforward inputs. Resolving this issue would require explicit and falsifiable modeling approaches, such as fitting an adaptation model and testing whether deviant responses exceed model predictions. The present study does not undertake such analyses, and therefore the neural mechanisms underlying the observed effects remain open to interpretation.

**Our Response:**

We agree. CSI and SSA quantify context-dependent response differences between the same stimulus presented as a deviant and as a standard, but these measures alone cannot distinguish deviance-specific processing or prediction-error signaling from use-dependent adaptation, including differential adaptation of stimulus-selective feedforward inputs.

The present study was designed to characterize how neural sensitivity to the tested click-train patterns varies across the IC–MGB–A1 pathway, rather than to identify the specific mechanism underlying the deviant–standard differences. Distinguishing adaptation from additional deviance-related components would require an explicit and falsifiable adaptation model, followed by testing whether the observed deviant responses exceed the responses predicted by that model. We did not perform such modeling in the present study.

We have therefore revised the Discussion to make this mechanistic limitation explicit and to clarify that the CSI/SSA findings demonstrate context-dependent sensitivity to the tested click-train patterns without establishing prediction-error signaling or identifying any other specific underlying mechanism.

**Revised text in the manuscript:**

“Second, the underlying neuronal mechanisms remain incompletely resolved. The principal neuronal analyses rely on CSI/SSA measures, which quantify differences between responses to the same stimulus presented as a deviant or as a standard. However, these measures do not by themselves distinguish deviance-specific processing or prediction-error signaling from use-dependent adaptation, including differential adaptation of stimulus-selective feedforward inputs. Resolving these alternatives would require an explicit model of adaptation dynamics and testing whether deviant responses exceed model predictions. Similarly, although cortical cooling provided causal evidence that cortical input contributes to MGB sensitivity under our paradigm, the present data do not determine whether this effect reflects predictive signaling, gain control, adaptation dynamics, changes in inhibitory–excitatory balance, or other forms of context-dependent modulation. Accordingly, the present results characterize context-dependent sensitivity to the tested click-train patterns across the auditory pathway without specifying the underlying neuronal mechanism.” (P.31, Line 15– P.32, Line 7)

Second, the study should be considered in the context of prior work. The hierarchical organization of the reported effects largely recapitulates findings previously reported by the authors' own group for frequency oddballs along the same IC-MGB-A1 pathway (Parras et al., 2017). In addition, the authors appropriately position the present work as a temporal-domain counterpart to Harpaz et al. (2021), which demonstrated stimulus-specific adaptation to configural "tone-cloud" identity that emerges strongly in cortex and only weakly in subcortical structures. This comparison helps clarify both the contribution and its limitations. Harpaz et al. employed spectrally balanced stimuli specifically designed to minimize the contribution of frequency-specific adaptation, whereas the click-train stimuli used here retain spectrotemporal differences that are only partially controlled by the long-term FFT-matched condition. Consequently, I view the present work as a valuable extension of an established line of research, expanding from spectral to temporal configurations, rather than as a fundamentally new conceptual advance.

These remaining points primarily concern the level of conceptual advance and journal-level significance rather than scientific rigor or validity. In my view, the manuscript is now scientifically sound and substantially improved.

**Our Response:**

We agree that the present study should be positioned within the established literature on hierarchical stimulus-specific adaptation rather than as a fundamentally new conceptual advance. Previous work by Parras et al. demonstrated increasing SSA along the IC–MGB–A1 pathway using frequency oddballs, while Harpaz et al. showed stronger cortical than subcortical sensitivity to configural tone-cloud identity.

The present study extends this established line of research to rapid click-train patterns that differ in their millisecond-scale temporal organization. We also agree that, unlike the spectrally balanced tone clouds used by Harpaz et al., our click-train stimuli retain short-term spectrotemporal differences. Moreover, matching the long-term FFT magnitude does not guarantee equivalent peripheral or neurogram-level representations. We have therefore revised the Discussion to clarify how the present findings relate to these previous studies and to avoid implying that our results isolate a purely temporal mechanism.

**Revised text in the manuscript:**

“The present findings build on previous studies of hierarchical stimulus-specific adaptation, including the work of Parras et al., who demonstrated increasing SSA along the IC–MGB–A1 pathway using frequency oddballs ” (P.29, Lines 2-4)
“Thus, our findings extend this established line of research from frequency oddballs and configural tone clouds to rapid click-train patterns. However, because these stimuli retain spectrotemporal differences, and because matching the long-term FFT magnitude does not ensure equivalent peripheral or neurogram-level representations, the present findings should not be interpreted as isolating a purely temporal mechanism.” (P.29, Line 16-21)

Reviewer #2: The revised version is substantially improved. Nevertheless, I found reading the paper difficult, due to the tendency of the authors to make very wide-ranging, wrong, claims, and then do experiments that do not engage with these claims at all. The detailed comments below are full of such issues.
There are in addition some technical issues and figures that obviously show wrong data.
Problem settings and irrelevant claims: These are mostly issues in the introduction and the discussion.

**Our Response:**

We thank you for recognizing that the revised manuscript has been substantially improved. We also appreciate your thoughtful concern that some statements in the Introduction and Discussion remained broader than the questions addressed by our experiments, and that several technical analyses and figures required further clarification or correction.

In response, we have systematically revised the manuscript to remove unsupported generalizations, align the framing more closely with the specific millisecond-scale click-train experiments and the accompanying data, and distinguish our empirical observations from mechanistic interpretations that the present study cannot establish. We have also carefully re-examined the relevant analyses, figures, terminology, and methodological descriptions. The specific changes made in response to each concern are detailed below, point by point.

P.4, top paragraph: This is mixing up temporal organizations at many time scales, which contribute to perception in different ways and are processed in different ways and different structures. For example, speech and music have strong temporal modulations at slow rates - for example, the typical rate of modulation of speech is about 4 Hz. These are very different from the time scales studied here, where interclick intervals are a few ms. This whole paragraph doesn't make sense (see e.g. among others Meibauer's 'Aspects of a theory of bullshit', <https://doi.org/10.1075/pc.23.1.04mei>). I would strongly urge the authors to drop the slop.

P. 4, line 9: 'the distinction between "what" a sound is and "when" it occurs' - I sincerely protest. When a sound occurs is when it occurs - this is totally independent of what a sound is. See e.g. Anne Danielsen's work on the 'p-center' (the time point to which a sound is perceptually assigned). These statements are simply nonsense.

P. 4, lines 14 and on - these statements are correct, but totally irrelevant to the data here, because they occur at very different time scales.

P. 4, lines 16-18 - this is a half-truth. We know that this information is represented in a way that allows neural discrimination - starting from the auditory nerve fibers, that provide a veridical representation of click trains at the tested rates here (interclick intervals of a few ms). We also know that they are represented in a way that allows perceptual discrimination - for example, a periodic click train has a pitch while a Poisson click train with the same average ICI doesn't. The contribution of this paper is in following this representation along the ascending auditory system, and showing that it continues to carry enough information to account for perception.

Frankly, I would simply remove this paragraph (P. 4 up to line 18) and start with the next one, which engages with the actual content of this paper.

**Our Response:**

We agree with your assessment that the original opening paragraph conflated temporal organization across substantially different timescales. In particular, it linked the relatively slow temporal modulations found in speech, music, and other complex sounds with the fine-grained, millisecond-scale click-train patterns examined in the present experiments. These phenomena involve different perceptual processes and neural mechanisms and therefore should not have been discussed within the same conceptual framing.

Following your recommendation, we have deleted the entire first paragraph of the original Introduction (previously p. 4, lines 1–18), including the discussion of speech, music, hierarchical temporal integration windows, and the distinction between “what” a sound is and “when” it occurs. The revised Introduction now begins directly with temporal integration and perceptual merging at short inter-click intervals, thereby focusing on the timescale and stimulus class examined in this study.

We have also revised the subsequent text to acknowledge that different millisecond-scale click-train patterns are already known to be perceptually distinguishable and that rapid click timing can be represented with high temporal fidelity in the auditory nerve and early auditory pathway. Periodic and Poisson click trains with similar mean ICIs, for example, can produce different perceptual qualities. Accordingly, we no longer present perceptual or neural discrimination of millisecond-scale click-train patterns as an unresolved question. Instead, we now define the principal objective of the study more specifically: to characterize how context-dependent sensitivity to these patterns is expressed across successive, higher levels, or feedback.

**Revised text in the manuscript:**

“While it is known that different millisecond-scale temporal configurations—such as periodic versus Poisson click trains—are perceived differently and can be represented with high temporal fidelity in the auditory nerve and early auditory pathway, it remains less clear how sensitivity to these rapidly varying temporal patterns is expressed at higher levels of the auditory system. Specifically, it is unclear how changes in such patterns are reflected in automatic deviance-related responses and how context-dependent sensitivity varies along the ascending auditory pathway.” (P.4, Lines 10-16)

P. 5, lines 5-9: it is known that different ms-scale temporal configuarions are perceived as distinct (e.g. periodic vs. Poisson). What is unknown is how these unitary objects are represented neurally in higher stations of the auditory system (in auditory nerve and CN, they are likely represented largely veridically by the temporal sequence of spikes).
The final paragraph of the introduction is much better. This language should be adopted also in the earlier parts of the introduction.

**Our Response:**

We agree. Different millisecond-scale temporal patterns, including periodic and Poisson click trains, are already known to produce distinct perceptual qualities. We have therefore removed the previous statement implying that the perceptual discriminability of such patterns was unknown.

We have also revised the earlier part of the Introduction to acknowledge that rapid click timing can be represented with high temporal fidelity in the auditory nerve and cochlear nucleus. The unresolved question is now framed more specifically as how sensitivity to rapid temporal patterns is expressed in higher levels of the auditory system, particularly under different stimulus contexts.

Following your suggestion, we have adopted the more focused language of the final paragraph in the earlier part of the Introduction. The revised text now emphasizes behavioral discrimination, context-dependent neural sensitivity, and comparisons across successive levels of the ascending auditory pathway, rather than presenting millisecond-scale temporal configurations as a previously unidentified auditory feature.

**Revised text in the manuscript:**

“While it is known that different millisecond-scale temporal configurations—such as periodic versus Poisson click trains—are perceived differently and can be represented with high temporal fidelity in the auditory nerve and early auditory pathway, it remains less clear how sensitivity to these rapidly varying temporal patterns is expressed at higher levels of the auditory system. Specifically, it is unclear how changes in such patterns are reflected in automatic deviance-related responses and how context-dependent sensitivity varies along the ascending auditory pathway.” (P.4, Lines 10-16)

P. 20 L2-6: This result is not surprising - the auditory system doesn't measure the long-term spectral magnitude. Rather, it is sensitive to the short-term spectro-temporal structure. The fact that sounds with identical long-term power spectra may sound very different is very well known (e.g. the 'ramp and damp' papers of Roy Patterson). Another well-known example is the Schroeder-phase harmonic complexes, which come in two polarities (causing basically a rising or a falling chirp structure); these sounds evoke very different activities already in the inner ear, reflected in different ABR structure. So the attribution of 'contributions from peripheral non-linearities' is wrong. These sounds have different spectro-temporal structure (the members of a pair are time-reversed), and so evoke different responses.
Even in the context of CSI, this result is not novel - Harpaz et al. (referenced in the manuscript a number of times) did all their work with sounds that share the same long-term spectrum, and showed largely the same results (larger effects of deviance in A1 than in IC and MGB).

**Our Response:**

We agree. Matching the long-term power spectrum does not make two sounds equivalent for the auditory system, because their short-term spectrotemporal structures can remain different and can produce different peripheral and central auditory responses. In our control stimuli, the members of each pair were time-reversed. They therefore had identical long-term FFT magnitude spectra but different time-domain waveforms and spectrotemporal structures. We agree that attributing the resulting response differences to “peripheral nonlinearities” was inaccurate, and we have removed this statement.

We have revised the interpretation of this control accordingly. The result shows only that differences in long-term spectral magnitude are not necessary for the observed oddball/context sensitivity. It does not demonstrate sensitivity to a purely temporal feature, because the paired sounds differed in their short-term spectrotemporal organization and may therefore engage different auditory representations.

We also agree that this finding is not novel in the broader context of stimulus-specific adaptation. Harpaz et al. previously demonstrated context-dependent responses to stimuli sharing the same long-term spectrum and reported stronger deviance-related effects in A1 than in IC and MGB. We have therefore removed any implication that the present control establishes a new principle and now discuss it as being consistent with, and extending to rapid click-train patterns, those previous findings.

**Revised text in the Results:**

“These results indicate that differences in long-term spectral magnitude are not required for the observed context-dependent responses. However, because the paired stimuli differed in their short-term spectrotemporal organization, this control does not isolate a purely temporal component.” (P.20, Lines 10-13)

**Revised text in the Discussion:**

“The additional control using time-reversed stimulus pairs matched in long-term FFT magnitude produced oddball/context-dependent differences in neuronal responses, with stronger CSI in A1 than in IC (S4 Fig). Because time reversal preserves the long-term FFT magnitude spectrum while altering the time-domain waveform and short-term spectrotemporal structure, this control indicates that differences in long-term spectral magnitude are not required for the observed context sensitivity. However, it does not isolate a purely temporal component of the neural response or exclude feedforward mechanisms sensitive to short-term spectrotemporal differences. Harpaz et al. ^[30]^ similarly demonstrated stimulus-specific adaptation to sounds with matched long-term spectra and observed stronger effects in A1 than in IC and MGB. Their findings, like ours, argue against explanations based solely on adaptation within fixed spectral channels while remaining compatible with mechanisms sensitive to short-term spectrotemporal structure. Thus, the present findings are consistent with these previous observations and extend them to the rapid click-train stimuli examined here.” (P.26, Line 16- P.27, Line 7)

P. 24, Lines 2-4: If you do not make the claim, you don't need to apologize for making it. You should remove these lines.
Lines 7-14: back to these unsubstantiated, inexact claims. How the auditory system encodes temporal structure at the ms scale has been intensively studied, for example in the pitch literature. Such claims are wrong, but also unnecessary. Remove them from the paper. What shown here is novel and interesting enough without them.
Fine timing of water flow, bird song, and wind in trees is NOT at the ms scale. This is poetic and sounds nice, but is deeply misleading.
P. 24, L 15 and following: Same issues. Spoken language has hierarchical temporal integration, but ms scale is not one of them. For example, Regev et al. 2024 (Nat. Hum. Behav., Fedorenko's group) show temporal receptive windows of 1, 4, and 6 words, where one word is about 250 ms long, two orders of magnitude longer than the ms scale studied in this paper. You can go higher to syllabic rate, which would give you another order of magnitude, but still more like 50 ms than like 4 ms.
As in the introduction, once past these generalities, the text stabilizes at things that make sense. But I would remove these incoherent ramblings.

**Our Response:**

We agree. Because the revised manuscript no longer claims that the stimuli isolate a purely temporal auditory dimension, the qualifying statement at the beginning of the Discussion was unnecessary. We have therefore removed this sentence.

We also agree that the original opening of the subsection made broad claims about millisecond-scale temporal processing that were not needed and were not sufficiently precise. In particular, the examples involving water flow, birdsong, insect calls, wind, speech, music, and hierarchical language processing introduced temporal phenomena operating at substantially different timescales from the 2–7 ms inter-click intervals studied here. We have removed these general statements.

The revised subsection now begins directly with rapid click trains, temporal coding, repetition-rate coding, and pitch-related processing. We also now acknowledge that millisecond-scale click-train processing has been studied extensively, and we frame the contribution of the present study more narrowly as examining behavioral discrimination and oddball/context sensitivity to selected click-train patterns across successive levels of the auditory pathway.

**Revised text in the Discussion:**

In the opening summary of the Discussion, we deleted the sentence:

“However, the present data do not establish that these stimuli isolate a purely temporal auditory dimension, nor do they identify the exact acoustic or temporal statistics encoded by the underlying neural circuits.”

We also replaced the opening paragraphs of the subsection “Click-Train Temporal Patterns and Their Auditory Representation” with the following text:

“Rapid click trains have been studied extensively in relation to temporal coding, repetition-rate coding, and pitch perception. They provide a useful approach for systematically manipulating millisecond-scale temporal patterns, although such manipulations also produce associated short-term spectrotemporal differences. At ICIs below 30 ms, regular click trains are generally perceived as a single pitch-like sound, consistent with temporal integration. Different periodic and irregular click trains can nevertheless evoke distinguishable perceptual qualities and neural responses. The present study builds on this literature by examining behavioral discrimination and oddball/context sensitivity to selected click-train patterns across successive levels of the auditory pathway.” (P.24, Line 12- 21)

Paragraph spanning P. 25-6: as I repeat again and again, these sounds are perceptually discriminable because they evoke different activity in the auditory system. This is not stated anywhere, but it should be the starting point for any discussion of the discrimination between these sounds. Afterward, one can ask whether there are 'summary statistics' that can be used to discriminate between them, and here comes the list of features discussed by the authors.
This bleeds into the next paragraph, which discusses the fact that neural signals were more discriminable than human behavior. There is no problem with this - behavior is a function of the neural signals, and therefore, you expect neural representations to be at least as fine as behavior, but they can be finer. There is no real 'dissociation' here.
Paragraph spanning P. 27-8: the fact that there is SSA to spectrally matched stimuli has already been shown (Harpaz et al.); I think this should be mentioned in this paragraph. As they argued, this fact falsifies mechanisms that occur within channel; they are however definitely not falsifying any feedforward mechanisms, which may be sensitive to spectro-temporal differences. I would remove this from the paper - it's wrong, but also unimportant for the main thrust of the results.

**Our Response:**

We agree. We have revised this part of the Discussion so that the interpretation begins from the premise that perceptual discrimination of these click-train patterns must arise from differences in auditory activity evoked by the sounds. The revised text now states this explicitly before discussing which acoustic or neural variables may support the observed behavioral discrimination.

We have also clarified that the present behavioral results do not identify a unique “summary statistic” or computational variable. We now list several possible accounts, including temporal regularity-related cues, pitch-like perceptual consequences of fast click trains, modulation-based representations, asymmetric temporal filtering/integration, short-term spectrotemporal structure, and other higher-order temporal descriptors. We also state that the present stimulus set was not designed to parametrically isolate these alternatives.

We further agree that measurable neural responses to stimulus pairs that are weakly discriminated behaviorally should not be interpreted as a true dissociation. We have therefore revised this paragraph to explain that behavioral performance depends on additional factors, including task demands, sensory memory, attention, decision criteria, and response mapping. The revised text now states that neural responses may contain information that is at least as fine-grained as, and sometimes finer than, what is expressed in overt behavior.

Finally, we have revised the discussion of the long-term FFT-magnitude-matched control. We now explicitly mention Harpaz et al. as previous evidence for stimulus-specific adaptation to sounds sharing the same long-term spectrum, including stronger effects in A1 than in IC and MGB. We have also removed any claim that this control rules out feedforward mechanisms. The revised text states that such findings are inconsistent with mechanisms based solely on adaptation within fixed spectral channels, but do not exclude feedforward mechanisms sensitive to short-term spectrotemporal differences.

**Revised text in the Discussion:**

“In a delayed match-to-sample task, participants reliably discriminated click trains that differed in temporal pattern while sharing the same mean inter-click interval and overall duration, with performance showing a graded pattern across stimulus pairs (Fig 1). The behavioral discriminability of these click-train patterns must ultimately arise from differences in the auditory activity evoked by the sounds. The present behavioral results therefore show that the tested click-train pairs generated neural differences that were available to perceptual decision-making in the delayed match-to-sample task. However, these results do not by themselves identify the exact acoustic or temporal statistics underlying that sensitivity. Moreover, oddball paradigms using pairs such as RN10–Con, RN10–TW30, Asc–TW30, TW10–TW30, TW50–TW30, and RN40–TW30 elicited clear MMN responses (Fig 2E-H), indicating automatic cortical sensitivity to changes in these patterns even without an explicit task. Thus, human behavioral and EEG results reveal sensitivity to click-train pattern differences, while the acoustic descriptors and computations that support this sensitivity remain unresolved.”

“A key open question is which acoustic or temporal statistics contribute to the observed behavioral discrimination. Although the tested click-train pairs shared the same mean inter-click interval, the present study does not identify the specific computational variable extracted by the auditory system. Several candidate accounts remain possible, including temporal regularity-related cues, pitch-like perceptual consequences of fast click trains, modulation-based representations, asymmetric temporal filtering/integration, or other higher-order temporal descriptors. Because the current stimulus set was not designed to isolate these alternatives parametrically, the present data do not allow a decisive distinction among them. A related issue concerns the relationship between behavioral and neural sensitivity. Some stimulus pairs that were weakly discriminable in the delayed match-to-sample task, such as TW_10_ versus TW_30_ in Fig 1C, nevertheless elicited measurable EEG differences in the oddball paradigm, such as TW_10_ versus TW_30_ in Fig 2G. This should not be interpreted as a strict dissociation between perception and neural representation. Behavioral performance depends on additional factors, including task demands, sensory memory, attention, decision criteria, and response mapping. Neural responses can therefore contain information that is at least as fine-grained as, and sometimes finer than, what is expressed in overt behavior. We therefore interpret the EEG results as evidence for context-dependent neural sensitivity to click-train pattern differences, rather than as evidence for a dissociation between neural and behavioral discrimination.”

“Because time reversal preserves the long-term FFT magnitude spectrum while altering the time-domain waveform and short-term spectrotemporal structure, this control indicates that differences in long-term spectral magnitude are not required for the observed context sensitivity. However, it does not isolate a purely temporal component of the neural response or exclude feedforward mechanisms sensitive to short-term spectrotemporal differences. Harpaz et al. similarly demonstrated stimulus-specific adaptation to sounds with matched long-term spectra and observed stronger effects in A1 than in IC and MGB. Their findings, like ours, argue against explanations based solely on adaptation within fixed spectral channels while remaining compatible with mechanisms sensitive to short-term spectrotemporal structure. Thus, the present findings are consistent with these previous observations and extend them to the rapid click-train stimuli examined here.” (P.25-27)

Technical issues that need to be corrected:
The description of stimulus synthesis is much better now. Thanks for making the sound files available. However, spectro-temporal analysis is not described in detail (just in passing in a few lines spanning P. 7-8, with a wrong reference to the supplementary information file - this is not the name under which it is provided) and is wrong the way it is provided. The analysis is clearly on a linear frequency - you can see the linearly-spaced harmonics in some of the spectrograms - but the frequency axis shown on the right hand side of each panel is logarithmic. When performing time-frequency analysis, the windows that have been used must be specified. I suggest that the authors use one of the simulations of the peripheral auditory system for that purpose - there are many good ones around (<https://amtoolbox.org/>, for example; I had that cited already in the previous review).

**Our Response:**

We thank you for this constructive comment. We agree that the previous description of the spectro-temporal analysis was insufficiently detailed, and that the original figure presentation could be misleading because the displayed frequency axis did not match the underlying analysis. We have therefore revised this part of the manuscript and replaced the previous spectrogram-style stimulus representation with a peripheral auditory representation based on cochlear filtering.

Following your suggestion, we generated cochleagrams using an ERB-spaced gammatone filterbank implemented in the Auditory Modeling Toolbox. This analysis provides a model-based peripheral auditory representation of the click-train stimuli after cochlear filtering and avoids the ambiguity associated with displaying a linear-frequency spectrogram on a logarithmic frequency axis. Because this representation is based on an auditory filterbank rather than a conventional short-time Fourier transform spectrogram, the analysis does not rely on a single STFT window length. Instead, the relevant parameters are the auditory filterbank spacing, number of filters, center-frequency range, envelope extraction, and amplitude scaling, which are now specified in the Methods.

The click-train audio files, including the additional spectrally matched click-train stimuli, are now provided in Supplementary Information S1 (Sound File). The corresponding AMT-based peripheral auditory representations are provided separately in Supplementary Information S1 Fig.

**Revised text in the manuscript:**

“Accordingly, the stimuli differed in their inter-click timing patterns and exhibited distinct model-based peripheral auditory representations after cochlear filtering. The audio files of the click-train stimuli are provided in Supporting Information S1 (Sound File), and the corresponding cochleagrams generated using the auditory model are shown in S1 Fig.” (P.7, Lines 8-11).

The description of this analysis procedure has also been added to the Methods section:

“The original click trains and these additional matched pairs were analyzed using a model-based peripheral auditory representation. Specifically, cochleagrams were generated using an ERB-spaced gammatone filterbank implemented in the Auditory Modeling Toolbox (AMT) [59]. The filterbank consisted of 96 auditory filters with center frequencies ranging from 80 Hz to 40 kHz. The envelope outputs of each auditory filter were converted to logarithmic amplitude values (dB) for visualization. The corresponding audio files are provided in supplementary information S1 (Sound File), and the cochleagram representations generated from these stimulus waveforms are shown in S1 Fig.” (P.33, Line 20 – P.34, Line 7)

Revised legend:

“S1 Fig. Peripheral auditory representations of click-train stimuli. Cochleagram representations of the ten click trains and two additional spectrally matched click-train pairs computed using an ERB-spaced gammatone filterbank implemented in the Auditory Modeling Toolbox (AMT).”

“S1 Sound File. Audio files of click-train stimuli and spectrally matched control stimuli. This file contains the ten click trains used as auditory stimuli and two additional pairs of click trains with identical frequency-domain magnitude spectra but different time-domain structures.”

Figure 1D: something wrong in the calculation of the d'. While TW10-TW30 performance is at chance level (Fig. 1C), the d' values center on 1. But d' of 1 corresponds to a fair bias towards correct responses.
I think the d' it was computed using the standard equations (transformed hit rates - transformed FA rates), which assume equal probability classes, not taking into account the fact that the test was conducted with 35% chance for same pairs.

**Our Response:**

We thank you for raising this important concern. We agree that the original presentation of Fig. 1C could have been misleading because the 35% and 65% reference lines reflected the unequal probabilities of identical and different trials, rather than chance performance for each individual stimulus pair. We have therefore revised Fig. 1C by relabeling these reference lines, reorganizing the presentation of the stimulus pairs, and clarifying the unequal condition proportions in both the figure legend and Methods.

We respectfully clarify that the conventional signal-detection measure

$$d^{'}=Z\left( H \right)-Z\left( FA \right)$$

does not require equal numbers or equal prior probabilities of identical and different trials. Both *H* and *FA* are conditional response probabilities:

$$H=P\left( \text{“different”}\text{ }\text{response}\mid\text{different}\text{ }\text{pair} \right),$$

$$FA=P\left( \text{“different”}\text{ }\text{response}\mid\text{identical}\text{ }\text{pair} \right).$$

The unequal probabilities of identical and different trials may influence the participant’s decision criterion, but they are not incorporated as prevalence weights in the calculation of $d^{'}$. Moreover, $d^{'}$quantifies discrimination sensitivity rather than a bias toward correct responses. Response bias is quantified separately using the criterion:

$$c=-\frac{Z\left( H \right)+Z\left( FA \right)}{2}$$

To directly assess whether the unequal 13:7 distribution produced a systematic response bias, we additionally calculated the response criterion for each participant and each different-pair condition using the participant-specific pooled false-alarm rate. For reference, we provide the corresponding distributions in Response Fig. 1 below. This figure has not been included in the revised manuscript.

**Response Figure 1. Response criterion calculated from behavioral responses.** Criterion values were calculated as c=−0.5[Z(H)+Z(FA)], with positive values indicating a bias toward “identical” responses and negative values indicating a bias toward “different” responses. Box plots show the distribution across participants. Dots indicate individual participant values.

Taking the TW_10_–TW_30_ condition as an example, criterion values were predominantly positive, indicating that participants generally adopted a conservative response strategy and tended to classify the stimulus pairs as “identical” when discrimination was difficult. Importantly, this response tendency was opposite to that expected if participants’ responses were driven primarily by the unequal condition proportions. Given that different conditions were more frequent than identical conditions at a ratio of 13:7, a prevalence-driven response tendency would, if anything, favor “different” responses.

Therefore, the relatively low proportion of correct responses in the TW_10_–TW_30_ condition is not consistent with a global tendency to select the more frequent “different” category. Rather, it is consistent with a conservative decision criterion favoring “identical” responses under difficult discrimination conditions.

The positive $d^{'}$ value is also not inconsistent with the relatively low hit rate. For TW_10_–TW_30_, the mean hit rate was $H=0.34$, whereas the mean participant-specific pooled false-alarm rate was substantially lower, $FA=0.04$. For illustration, these group-level values yield

$$Z\left( 0.34 \right)-Z\left( 0.04 \right)\approx1.34,$$

demonstrating why a positive criterion can coexist with a relatively low condition-specific correct-response rate. All $d^{'}$ and criterion values were calculated separately for each participant before group-level summarization; therefore, this illustrative calculation is not intended to exactly reproduce the plotted group-mean $d^{'}$.

These results indicate that the positive $d^{'}$values cannot be attributed to a prevalence-driven tendency to respond “different”; rather, they were observed despite a response bias in the opposite direction.

**Revised text in the manuscript:**

“The mean response accuracies for the TW_10_-TW_30_, Con-RN_10_ and RN_10_-TW_10_ pairs were 0.34, 0.35, and 0.40 (Fig 1C), respectively, indicating that these pairs were difficult to discriminate behaviorally.” (P. 8, Lines 23-25)

**Fig. 1C legend:** “Behavioral performance across participants. Response accuracy is shown as the mean correct response rate for each tested click-train pair. … Dashed horizontal lines indicate the overall response accuracies expected from consistently choosing the more frequent trial type ("different", 0.65) or the less frequent trial type ("identical", 0.35), reflecting the unequal probabilities of the two trial types in the experimental design.”

**Methods:** “We selected 20 sound pairs, as shown in Fig. 1C, each presented 40 times per session. Among these 20 pairs, 7 were identical pairs and 13 were different-stimulus trials. Therefore, identical and different-stimulus trials accounted for 35% and 65% of all trials, respectively”. (P. 37, Lines 16-19)

P. 14, Fig. 3C and text: '…both showed enhanced negative-going responses when presented as deviants…' - I frankly don't see it. The red lines, if anything, are above the blue lines. If there's anything that I miss, please show that explicitly in the figures.
I already commented on that in the previous review. As presented, the data in Fig. 3 do not support the text that describe them. Some red lines are above the blue lines, some are below; in some the peak-to-peak seems to be larger for deviants than for standards, and some the reverse.
I would accept these data as evidence that the stimuli are represented differentially somewhere along the pathway, but I would remove the text about 'enhanced responses to deivants'. Frankly, I don't see anything consistent about that.

**Our Response:**

We agree. The ECoG responses in Fig. 3C do not show a uniform enhancement of negative-going responses when the stimuli were presented as deviants. As you noted, the direction and time course of the deviant–standard differences vary across stimulus conditions, with some deviant traces lying above and others below the corresponding standard traces.

We have therefore removed the statement that the stimuli showed “enhanced negative-going responses” when presented as deviants. We now describe the result more conservatively as context-dependent differences between deviant and standard responses in auditory cortical ECoG responses. We also revised the Fig. 3 legend accordingly, so that the figure is presented as evidence for context-dependent cortical sensitivity to click-train pattern changes, rather than as evidence for a consistent negative-going deviant response.

**Revised text in the Results:**

“Across rats, auditory cortical ECoG responses differed between standard and deviant presentations when the same physical click-train stimulus was compared across its two roles. However, the direction and time course of these deviant–standard differences varied across stimulus conditions, and deviant responses were not uniformly larger or more negative than standard responses. For example, RN_10_ and TW_50_ showed significant context-dependent differences between deviant and standard responses when the same stimuli were presented as standards or deviants (Fig 3C). Similar context-dependent differences were also observed for additional stimulus pairs, including RN60–Con and Asc–TW_30_ (Fig 3D–E).” (P. 14, Lines 7-15)

**Revised Fig. 3 legend:**

**(C-E)** “Population-level ECoG responses to the same physical click-train stimuli when presented as standards or deviants in the oddball paradigm illustrated in (A)”

Other comments, largely in the order in which they occur in the text:
P. 7 and Table S1: Note that the 'Asc' stimulus has a descending pitch, and the 'Dec' stimulus has an ascending pitch. This confused me at first.

**Our Response:**

We agree that the labels “Asc” and “Dec” could be confusing. These labels refer to the direction of change in inter-click interval change across the click train, not to perceived pitch direction. Because repetition rate and pitch-like perceptual correlates are inversely related to ICI, the Asc stimulus has progressively increasing ICIs and therefore a descending repetition rate and a descending pitch trend, whereas the Dec stimulus has progressively decreasing ICIs and therefore an increasing repetition rate and an ascending pitch trend.

We have clarified this point when the stimuli are first described and added an explanatory note to Table S1.

**Revised text in the manuscript:**

“These labels refer to the direction of change in ICI, not to perceived pitch direction: because repetition rate is inversely related to ICI, Asc corresponds to a descending repetition-rate/pitch trend, whereas Dec corresponds to an ascending repetition-rate/pitch trend.” (P.6, Lines 13-16)

**Revised Table S1 note:**

“ICI increases progressively from 2 ms to 6 ms (decreasing repetition rate; descending pitch trend)”

“ICI decreases progressively from 6 ms to 2 ms (increasing repetition rate; ascending pitch trend)”

P. 9 L6: drop 'psychological' - you likely mean 'behavioral'. Also, on the same page, 'reference proportion' is incorrect ('chance level' or something like that; at least, say 'probability' rather than 'proportion').

**Our Response:**

We agree. We have replaced “psychological” with “behavioral,” which more accurately describes the delayed match-to-sample performance measured in this experiment.

We have also revised the terminology concerning the 0.35 and 0.65 values. These values refer to the probabilities of same and different trials in the task design, rather than to a general psychological “proportion.” We therefore replaced “reference proportion” with “reference probability” and clarified that these probabilities are shown as reference lines in the behavioral performance figure.

**Revised text in the manuscript:**

“they exhibited similar behavioral response patterns characterized by lower discrimination performance between the TW10-TW30, Con-RN10, and RN10-TW10 pairs …” (P.8, Line 21)

“all significantly greater than the reference probability of 0.65 corresponding to consistently selecting the more frequent “different” response (p < 0.01 for all five pairs, Kolmogorov–Smirnov test), indicating that participants reliably discriminated these stimulus pairs.” (P.9, Lines 3-6)

P. 9, L. 15-16: you only know that the discriminated the stimuli, you don't know what features they used for that. Drop the line about the participants recognizing the objects as distinct entities ('distinct' and 'different' are not the same thing).

**Our Response:**

We agree. The behavioral task shows that participants could discriminate several click-train pairs, but it does not identify the specific acoustic or perceptual features that supported this discrimination. We have therefore removed the statement that participants “recognized the two temporally merged auditory objects as distinct entities.”

The revised text now states more conservatively that participants reliably discriminated these stimulus pairs, without specifying the cues or perceptual features underlying their performance.

**Revised text in the manuscript:**

“The mean response accuracies for the TW_10_-TW_30_, Con-RN_10_ and RN_10_-TW_10_ pairs were 0.34, 0.35, and 0.40 (Fig 1C), respectively, indicating that these pairs were difficult to discriminate behaviorally. In contrast, the mean response accuracies for the Con-Asc, Asc-TW_30_, RN_60_-TW_10_, Asc-Dec and RN_60_-TW_70_ pairs were 0.97, 0.96, 0.92, 0.92 and 0.91, respectively (Fig 1C), all significantly greater than the reference probability of 0.65 corresponding to consistently selecting the more frequent “different” response (p < 0.01 for all five pairs, Kolmogorov–Smirnov test), indicating that participants reliably discriminated these stimulus pairs.” (P.8, Line 23- P.9, Line 6)

P. 10, l4-5: the authors correctly state that the behavioral results do not determine the specific features that were used for discrimination, implying (at least to me) that they are going to do that. However, they do something completely different - they study the neural representations of these sounds. Remove the sentence 'However, the present behavioral data…'.

**Our Response:**

We agree. The original sentence could imply that the following analyses would identify the specific acoustic or temporal descriptors used by participants for behavioral discrimination. However, the purpose of the subsequent EEG experiments was instead to test whether changes among these click-train patterns elicited context-dependent differences in neural response in an oddball paradigm.

We have therefore deleted the sentence “However, the present behavioral data do not determine which specific acoustic or temporal descriptors best account for this discrimination” from this part of the Results. Following this deletion, the next sentence now directly introduces the EEG oddball experiment.

P 10 L 7-10: There's a subtle point here that is obscured by all the enveloping text. '…click-train patterns … differentially represented in neural activity' requires a different experiment than the use of oddball paradigms. In the same auditory station, different click train patterns can evoke the same neural activity, and yet also evoke differential deviant-standard responses. Oddball sequences demonstrate that over the whole pathway creating the responses, the two stimuli are represented differentially; not that the recorded neural response represents these stimuli differentially. It's completely fine that the authors used oddball design, and I'm very happy with their results, but yet again they shouldn't state things they didn't do.

**Our Response:**

We agree. An oddball paradigm does not by itself demonstrate that a recorded neural response at a specific auditory station directly encodes different click-train patterns as distinct stimulus representations. Rather, oddball responses reflect context-dependent differences between deviant and standard responses generated by the auditory system, indicating that the neural processes contributing to the recorded response are sensitive to changes between the tested stimuli.

We have therefore removed the phrase “differentially represented in neural activity” from this part of the Results. The revised text now describes the EEG and pathway-level experiments more conservatively as examining whether changes among click-train patterns produce context-dependent differences between deviant and standard responses across different levels of the auditory system.

**Revised text in the manuscript:**

“To examine whether changes among these click-train patterns elicited context-dependent differences between deviant and standard responses, we used several oddball paradigms to assess neural sensitivity to these stimuli.” (P.9, Lines 18-20)

Pp. 10-12, EEG recordings: P. 10 l7 and on, and also Fig. 2A, seems to imply that in the oddball sequences only TW30 was used as standard. However, later on in the text it turns out that in some recordings pairs of sounds were used alternatively as standard and deviant (Fig. 2E and 2F), and the in the multi-deviant sequences, sometimes RN10 was used as the standard. Please explain exactly what you did.

**Our Response:**

We agree that the previous description of the EEG oddball paradigms was not sufficiently explicit and could give the impression that TW30 was always used as the standard stimulus. In fact, the passive EEG experiment consisted of four oddball sessions, with different standard-deviant assignments across sessions, as described in the Methods section. (EEG experiment procedures Passive Listening Sessions 2-5).

Figure 2 was intended to illustrate representative examples of these oddball configurations rather than to present every standard-deviant assignment. Specifically, Fig. 2E and 2F show contextual comparisons in which the same physical stimulus was presented as a standard stimulus in one oddball session and as a deviant stimulus in another session, providing an opportunity to examine whether neural responses differed depending on stimulus context. Fig. 2G and 2H present representative results from two multi-deviant oddball sessions using TW30 and RN10 as the standard stimuli, respectively.

We have revised the Results to explicitly describe the standard and deviant assignments and clarified that Fig. 2 presents representative examples rather than the complete set of oddball configurations. The Fig. 2 legend has also been revised accordingly to clarify that the illustrated oddball configurations are representative examples rather than the full set of standard–deviant assignments used in the EEG experiments.

**Revised text in the manuscript:**

“To examine whether differences among click-train patterns elicited context-dependent differences between deviant and standard, we used several oddball paradigms to assess neural sensitivity to these stimuli. Across four passive EEG sessions, different click-train patterns were assigned as standard and deviant stimuli depending on the experimental design (see Methods, EEG experiment procedures, Passive Listening Sessions 2–5). Figure 2 presents representative examples of these oddball configurations. In the representative configuration shown in Fig 2A, TW_30_ was presented as the standard stimulus, whereas TW_50_, Asc, RN_40_, TW_10_, RN_10_, and RN_60_ served as deviant stimuli. Among these deviant stimuli, TW_50_, Asc, and RN_40_ were selected as representative examples for illustration. Deviant stimuli were randomly interspersed among repetitive standard stimuli, with an average of nine standard stimuli followed by one deviant stimulus at a 300-ms inter-stimulus interval.” (P. 9, Line 18- P.10, Line 7)

“In our human subject population, significant differences between deviant and standard emerged for multiple click-train patterns, particularly within the 100–200 ms post-stimulus window. To examine whether these differences varied with the contextual role of the stimulus, we compared neural responses to the same physical stimulus when it served as a standard stimulus in one passive EEG session and as a deviant stimulus in another session. Fig 2E and 2F show representative examples of such contextual comparisons, in which significant differences between deviant and standard responses were observed for the RN_10_–Con and RN_10_–TW_30_ comparisons, respectively. We then examined the multi-deviant oddball paradigm. Fig 2G and 2H show representative examples in which TW_30_ (Fig 2G) and RN_10_ (Fig 2H) served as the standard stimuli, respectively.” (P. 12, Lines 5-15)

Paragraph spanning Pp. 10 and 11 - why did you select these 27 channels and no others?

**Our Response:**

We agree that the rationale for selecting the 27 EEG channels was not sufficiently described. Following our previous study (Xu H, et al., Commun Biol, 2025), these channels were predefined as a region of interest over the temporal and occipital regions, where robust auditory responses to click trains had previously been identified. Thus, the channels were selected independently of the deviant–standard differences observed in the present dataset.

We have added this clarification to the Results section accordingly.

**Revised text in the manuscript:**

“Following our previous study [9], we predefined a region of interest comprising 27 EEG channels over the temporal and occipital regions, where robust auditory responses to click trains had previously been identified.” (P. 10, Lines 15-17)

P. 12 l. 7: …when the deviants were Asc, …. etc.' - remove the etc., and state exactly which conditions.

**Our Response:**

We have removed the previous statement containing “etc.” and revised the Results to avoid implying that the listed deviant stimuli represented the full set of experimental conditions. Instead, we now describe the stimuli shown in Fig. 2A as representative examples of the oddball configuration. The full set of standard–deviant assignments across all passive EEG sessions are provided in the Methods section (EEG experiment procedures, Passive Listening Sessions 2–5).

**Revised text in the manuscript:**

“Across four passive EEG sessions, different click-train patterns were assigned as standard and deviant stimuli depending on the experimental design (see Methods, EEG experiment procedures, Passive Listening Sessions 2–5). Figure 2 presents representative examples of these oddball configurations. In the representative configuration shown in Fig 2A, TW_30_ was presented as the standard stimulus, whereas TW_50_, Asc, RN_40_, TW_10_, RN_10_, and RN_60_ served as deviant stimuli. Among these deviant stimuli, TW_50_, Asc, and RN_40_ were selected as representative examples for illustration.” (P. 9, Line 20- P.10, Line 5)

“In our human subject population, significant differences between deviant and standard responses emerged for multiple click-train patterns, particularly within the 100–200 ms post-stimulus window.” (P. 12, Lines 5-7)

**Fig 2 legend revised**: “**(A)** Representative example of an oddball paradigm configuration used in the EEG experiments. In this example, the standard sound (TW30) is presented at a 300-ms inter-stimulus interval, followed by a deviant sound after 7-11 standard repetitions. The deviant sound is randomly chosen from a set of stimuli (TW_50_, Asc, RN_40_, TW_10_, RN_10_, and RN_60_). Different standard-deviant assignments were used across passive EEG sessions (see Methods).”

P. 12 L. 21, again 'psychological' instead of 'behavioral'.

**Our Response:**

We agree. We have replaced “psychological” with “behavioral” to describe the findings more accurately.

**Revised text in the manuscript:**

“Building on the behavioral and electrophysiological evidence obtained in humans” (P. 12, Line 21)

P. 18 L11 and other places: the reference to the supplemental figures is stragne ('…are summarized in S1 Fig.' and similar texts elsewhere.

**Our Response:**

We agree that phrases such as “are summarized in S1 Fig.” were awkward and did not specify clearly what was presented in the Supporting Information. We have revised this sentence to describe the contents of the figure directly and checked similar references to the Supporting Information throughout the manuscript for clarity and consistency.

**Revised text in the manuscript:**

“CSI distributions by subdivision for MGV, MGD, CIC, and EIC across the three click-train stimulus pairs are shown in S2 Fig.” (P.18, Lines 13-14)

“we analyzed a subset of neurons that showed significantly positive CSI values for a pair of complex tones (CT; see Methods, Session 5: Complex Tone Oddball Procedure) in the oddball paradigm, including neurons from A1 (n = 94), MGB (n = 109), and IC (n = 140). … Neuronal responses and corresponding CSI values for these neurons are shown in S3 Fig.” (P.18, Line 18- P.19, Line 3)

Fig. 5: this figures incidentally shows that at least in IC, different click trains indeed evoke different responses! This is obvious in 5C, 5H and 5M (line graphs). This is actually an interesting point: whereas in IC different stimuli give rise to different responses (and therefore can be discriminated by the actual responses), in MGB and A1 potentially these differences are reduced, but the two areas are still sensitive to the identity of the stimuli as shown by the deviant-standard contrast.

**Our Response:**

We thank you for this insightful observation. We agree that the population responses in Fig. 5C, H, and M show visibly different temporal response patterns for different click trains in the IC, whereas the corresponding response patterns in the MGB and A1 are more similar across stimuli.

One possible explanation is that IC neurons more precisely follow the individual clicks or other fine-scale temporal features of the click trains, thereby preserving stimulus-specific temporal response patterns. At higher auditory levels, this click-by-click temporal following may become less prominent, even though neurons remain sensitive to stimulus identity and contextual changes.

The present study did not formally quantify temporal following or the separability of stimulus-evoked temporal response patterns across auditory levels. We therefore consider this an interesting observation and a potentially important direction for future investigation.

P. 19, L6-8: I don't fully follow the logic, and the new text, while much clearer than the original version, still doesn't convince me. The CSI is a joint property of the neuron and of the stimuli used to test it. The units used in this analysis were selected by their large CSI on the complex tone, so why does the fact that the weaker click-train CSI in IC cannot be explained solely by the generally weaker SSA in IC? It could be that there is another subset of neurons with very large CSI for the click-trains.
What this controls show is that large CSI is, well, a joint property of the tested neuron and the tested stimulus. Large CSI for one stimulus does not imply large CSI for other stimuli. I frankly don't see anything else here.

**Our Response:**

We agree that CSI is jointly determined by the response properties of the neuron and the specific stimulus pair used to test it. Therefore, a high CSI for one stimulus pair does not necessarily predict a high CSI for another stimulus pair. We also agree that selecting neurons according to their complex-tone CSI cannot identify every neuron that might show a large CSI specifically for click trains.

The purpose of this control analysis was not to assume that complex-tone CSI directly reflects click-train CSI, but rather to test whether the reduced click-train CSI observed in the IC could be explained simply by a general difference in the capacity of IC neurons to express SSA. We therefore selected neurons that exhibited significantly positive CSI values for an independent complex-tone pair, thereby enriching the analysis for neurons that demonstrated SSA for at least one stimulus condition. Importantly, within this selected population, complex-tone CSI was significantly higher in the IC than in A1, indicating that the selected IC neurons were not generally less sensitive to the complex-tone context manipulation. Nevertheless, when these same neurons were examined using click-train stimuli, CSI remained significantly lower in the IC than in A1.

These results suggest that the lower click-train CSI in the IC cannot be attributed solely to a general reduction in SSA expression across IC neurons. We acknowledge that this analysis does not exclude the possibility of an IC subpopulation exhibiting particularly strong SSA specifically for click-train stimuli, because neuron selection was based on complex-tone rather than click-train CSI. We have therefore revised the manuscript to clarify the rationale and limitation of this control analysis and have moderated our interpretation accordingly.

**Revised text in the manuscript:**

“To examine whether a general difference in SSA expression across auditory regions could account for the reduced click-train CSI, we analyzed a subset of neurons that showed significantly positive CSI values for a pair of complex tones (CT; see Methods, Session 5: Complex Tone Oddball Procedure) in the oddball paradigm, including neurons from A1 (n = 94), MGB (n = 109), and IC (n = 140). This selection provided a population of neurons that exhibited SSA for an independent stimulus class, allowing us to test whether reduced click-train CSI persisted among neurons capable of expressing SSA. Neuronal responses and corresponding CSI values for this subset are shown in S3 Fig.” (P.18, Line 17- P.19, Line 3)

“Thus, the lower click-train CSI in the IC was not explained solely by a general difference in SSA expression across auditory regions. However, this analysis does not exclude the existence of an IC subpopulation with particularly large CSI for specific click-train pairs, because neurons were selected based on complex-tone CSI. Accordingly, we interpret the higher click-train CSI in A1 as evidence of stronger context-dependent sensitivity to the tested click-train patterns in A1 than in IC.” (P.19, Lines 14-20)
