# Supplementary material for: Distinct millisecond-scale click-train patterns evoke discriminable behavioral and neural responses in humans and rats
Source: PLoS Biol. 2026 Oct 8;24(10):e3004046. doi: 10.1371/journal.pbio.3004046 (PMC13686464; doi:10.1371/journal.pbio.3004046)
Supplement: Peer review history

# Editor report: Decision Letter 0

**DOI:** 10.1371/journal.pbio.3004046.r001

**Authors:** Christian Schnell, PhD

2 Dec 2025

Dear Manolo,

Thank you for submitting your manuscript entitled "Hierarchical Encoding of Millisecond-Scale Temporal Configuration of Sound in the Mammalian Auditory Pathway" for consideration as a Research Article by PLOS Biology.

Your manuscript has now been evaluated by the PLOS Biology editorial staff as well as by an academic editor with relevant expertise and I am writing to let you know that we would like to send your submission out for external peer review.

However, before we can send your manuscript to reviewers, we need you to complete your submission by providing the metadata that is required for full assessment. To this end, please login to Editorial Manager where you will find the paper in the 'Submissions Needing Revisions' folder on your homepage. Please click 'Revise Submission' from the Action Links and complete all additional questions in the submission questionnaire.

Once your full submission is complete, your paper will undergo a series of checks in preparation for peer review. After your manuscript has passed the checks it will be sent out for review. To provide the metadata for your submission, please Login to Editorial Manager (https://www.editorialmanager.com/pbiology) within two working days, i.e. by Dec 04 2025 11:59PM.

If your manuscript has been previously peer-reviewed at another journal, PLOS Biology is willing to work with those reviews in order to avoid re-starting the process. Submission of the previous reviews is entirely optional and our ability to use them effectively will depend on the willingness of the previous journal to confirm the content of the reports and share the reviewer identities. Please note that we reserve the right to invite additional reviewers if we consider that additional/independent reviewers are needed, although we aim to avoid this as far as possible. In our experience, working with previous reviews does save time.

If you would like us to consider previous reviewer reports, please edit your cover letter to let us know and include the name of the journal where the work was previously considered and the manuscript ID it was given. In addition, please upload a response to the reviews as a 'Prior Peer Review' file type, which should include the reports in full and a point-by-point reply detailing how you have or plan to address the reviewers' concerns.

During the process of completing your manuscript submission, you will be invited to opt-in to posting your pre-review manuscript as a bioRxiv preprint. Visit http://journals.plos.org/plosbiology/s/preprints for full details. If you consent to posting your current manuscript as a preprint, please upload a single Preprint PDF.

Feel free to email us at plosbiology@plos.org if you have any queries relating to your submission.

Kind regards,

Christian

Christian Schnell, PhD

Senior Editor

PLOS Biology

cschnell@plos.org

# Peer review: Decision Letter 1

**DOI:** 10.1371/journal.pbio.3004046.r002

**Authors:** Christian Schnell, PhD

27 Jan 2026

Dear Manolo,

Thank you for your patience while your manuscript "Hierarchical Encoding of Millisecond-Scale Temporal Configuration of Sound in the Mammalian Auditory Pathway" was peer-reviewed at PLOS Biology. Your manuscript has been evaluated by the PLOS Biology editors, an Academic Editor with relevant expertise, and by several independent reviewers.

As you will see in the reviewer reports, which can be found at the end of this email, although the reviewers find the work potentially interesting, they have also raised a substantial number of important concerns. Based on their specific comments and following discussion with the Academic Editor, it is clear that a substantial amount of work would be required to meet the criteria for publication in PLOS Biology. However, given our and the reviewer interest in your study, we would be open to inviting a comprehensive revision of the study that thoroughly addresses the reviewers' comments, focusing on the comments of Reviewer 2 and Reviewer 3. We would not expect you to experimentally address Reviewer 1's request for additional experimental data, but ask you to discuss these as opportunities for future studies and/or as limitations of the current study (unless of course you have some of the data already). Given the extent of revision that would be needed, we cannot make a decision about publication until we have seen the revised manuscript and your response to the reviewers' comments. Your revised manuscript would need to be seen by the reviewers again, but please note that we would not engage them unless their main concerns have been addressed.

Having discussed the reviews with the Academic Editor, we think you should overall reframe the paper to be more conservative in its approach (all reviewers agree that this is not a study of a uniquely temporal dimension). Furthermore, the spectral comments mentioned by Reviewer 2 and 3 will be very important to fully address.

In addition to these revisions, you will need to complete some formatting changes, which you will receive in a follow up email. A member of our team will be in touch with a set of requests shortly.

We appreciate that these requests represent a great deal of extra work, and we are willing to relax our standard revision time to allow you 6 months to revise your study. Please email us (plosbiology@plos.org) if you have any questions or concerns, or envision needing a (short) extension.

At this stage, your manuscript remains formally under active consideration at our journal; please notify us by email if you do not intend to submit a revision so that we may withdraw it.

**IMPORTANT - SUBMITTING YOUR REVISION**

Your revisions should address the specific points made by each reviewer. Please submit the following files along with your revised manuscript:

1. A 'Response to Reviewers' file - this should detail your responses to the editorial requests, present a point-by-point response to all of the reviewers' comments, and indicate the changes made to the manuscript.

*NOTE: In your point by point response to the reviewers, please provide the full context of each review. Do not selectively quote paragraphs or sentences to reply to. The entire set of reviewer comments should be present in full and each specific point should be responded to individually, point by point.

You should also cite any additional relevant literature that has been published since the original submission and mention any additional citations in your response.

2. In addition to a clean copy of the manuscript, please also upload a 'track-changes' version of your manuscript that specifies the edits made. This should be uploaded as a "Revised Article with Changes Highlighted " file type.

*Resubmission Checklist*

When you are ready to resubmit your revised manuscript, please refer to this resubmission checklist: https://plos.io/Biology_Checklist

To submit a revised version of your manuscript, please go to https://www.editorialmanager.com/pbiology/ and log in as an Author. Click the link labelled 'Submissions Needing Revision' where you will find your submission record.

Please make sure to read the following important policies and guidelines while preparing your revision:

*Published Peer Review*

Please note while forming your response, if your article is accepted, you may have the opportunity to make the peer review history publicly available. The record will include editor decision letters (with reviews) and your responses to reviewer comments. If eligible, we will contact you to opt in or out. Please see here for more details:

https://blogs.plos.org/plos/2019/05/plos-journals-now-open-for-published-peer-review/

*PLOS Data Policy*

Please note that as a condition of publication PLOS' data policy (http://journals.plos.org/plosbiology/s/data-availability) requires that you make available all data used to draw the conclusions arrived at in your manuscript. If you have not already done so, you must include any data used in your manuscript either in appropriate repositories, within the body of the manuscript, or as supporting information (N.B. this includes any numerical values that were used to generate graphs, histograms etc.). For an example see here: http://www.plosbiology.org/article/info%3Adoi%2F10.1371%2Fjournal.pbio.1001908#s5

*Blot and Gel Data Policy*

We require the original, uncropped and minimally adjusted images supporting all blot and gel results reported in an article's figures or Supporting Information files. We will require these files before a manuscript can be accepted so please prepare them now, if you have not already uploaded them. Please carefully read our guidelines for how to prepare and upload this data: https://journals.plos.org/plosbiology/s/figures#loc-blot-and-gel-reporting-requirements

*Protocols deposition*

To enhance the reproducibility of your results, we recommend that if applicable you deposit your laboratory protocols in protocols.io, where a protocol can be assigned its own identifier (DOI) such that it can be cited independently in the future. Additionally, PLOS ONE offers an option for publishing peer-reviewed Lab Protocol articles, which describe protocols hosted on protocols.io. Read more information on sharing protocols at https://plos.org/protocols?utm_medium=editorial-email&utm_source=authorletters&utm_campaign=protocols

Thank you again for your submission to our journal. We hope that our editorial process has been constructive thus far, and we welcome your feedback at any time. Please don't hesitate to contact us if you have any questions or comments.

Sincerely,

Christian

Christian Schnell, PhD

Senior Editor

PLOS Biology

cschnell@plos.org

-----------------------------------------

REVIEWS:

Reviewer #1: This manuscript presents an ambitious, multi-level study combining human psychophysics, human EEG, rat ECoG, large-scale extracellular recordings, laminar analysis, and cortical cooling to investigate sensitivity to millisecond-scale temporal configuration in click trains. The cross-species scope and technical breadth are impressive, and the finding that temporally merged click trains can nevertheless be discriminated is interesting and potentially novel.

However, despite the richness of the dataset, the study remains largely descriptive. The central conceptual claim, that millisecond-scale temporal configuration constitutes a feature-like dimension of sound, is not supported by clear mechanistic insight or principled stimulus-to-neural mapping. The links between behavioral, cortical, subcortical, and circuit-level results are often inferential rather than explanatory. As a result, the manuscript does not yet achieve the level of biological insight implied by its title and framing.

Below I outline several major concerns that, in my view, cannot be resolved without substantial redesign or new experiments.

1. Lack of explicit explanation of what temporal statistics are encoded: The authors define "temporal configuration" as the ordering of inter-click intervals within a temporally merged click train, and they convincingly show that differences in such configurations can drive behavioral discrimination and neural deviance responses. They further argue, at a conceptual level, that predictive coding and corticothalamic feedback are likely involved. However, the manuscript does not identify which statistical properties of the click trains are actually encoded or compared by the auditory system. Across experiments, different click trains are effectively treated as categorical auditory objects, rather than as stimuli parametrized along defined temporal dimensions.

Specifically:

- No explicit temporal statistic is tested or quantified, such as variance, monotonicity, transition probability, entropy, or higher-order correlations.

- Neural responses are not related to any formal description of the temporal structure.

- The study does not examine whether neural sensitivity varies continuously with any measurable temporal parameter.

As a result, it remains unclear how millisecond-scale temporal structure is represented in neural activity, beyond the observation that some configurations elicit stronger deviant responses than others. The claim that temporal configuration is encoded as a feature therefore remains descriptive rather than mechanistic.

What would be needed to address this concern:

- Parametric manipulation of a single temporal statistic while holding others constant.

- Regression or encoding analyses relating neural responses or CSI values to explicit temporal metrics.

- Model-based analyses comparing alternative candidate statistics that could explain behavioral and neural discriminability.

These analyses would require substantial additional work and are not straightforward extensions of the current dataset.

2. Insufficient justification of stimulus design and unexplained behavioral-neural dissociations: The manuscript employs a large set of click trains (Asc, Dec, TW10-70, RN10-60), which are clearly described procedurally. However, the rationale for choosing these specific configurations, and their hypothesized perceptual or computational relevance, is not articulated. More importantly, several results reveal clear dissociations that are not addressed. For example:

- Human participants fail to behaviorally discriminate TW10 versus TW30, yet these same stimuli elicit robust neural differences in EEG oddball paradigms.

- The manuscript does not discuss what such dissociations imply about perceptual access, task demands, or neural readout.

Without a principled account of why particular configurations should be similar or different, both behaviorally and neurally, the interpretation of these results remains ambiguous. In the absence of such an account, the millisecond-scale temporal structure risks becoming incidental rather than explanatory.

What would be needed to address this concern:

- A theoretical or computational justification for the chosen stimulus classes.

- Analyses explicitly comparing behavioral discriminability with neural discriminability across stimulus pairs.

- Discussion, or direct testing, of whether neural differences reflect subthreshold representations, predictive mismatch, or task-independent encoding.

Addressing these points would likely require new behavioral paradigms or explicit modeling, beyond revision-level changes.

3. Cross-species generality is asserted without validating perceptual equivalence: A central claim of the paper is that sensitivity to temporal configuration is conserved across species. This claim is based on the use of identical stimuli in humans and rats, and the observation of MMN or MMN-like responses in both. However, the perceptual status of these stimuli is never validated in rats. In humans, the study explicitly relies on the assumption that the click trains are perceptually merged and that individual ICIs are not consciously detectable. Whether the same assumption holds in rats is not tested.

Given known species differences in temporal resolution and auditory processing, it is not trivial to assume that identical stimuli occupy equivalent perceptual regimes across species. Without behavioral validation or alternative controls, the claim of conserved temporal configuration processing remains indirect.

What would be needed to address this concern:

- Behavioral assays in rats testing discrimination or generalization across click-train configurations.

- Physiological validation that the stimuli fall below perceptual segregation thresholds in rats.

- At minimum, control stimuli demonstrating that rat responses are not driven by simpler cues.

These additions would constitute major new experiments.

4. Cortical cooling results lack a mechanistic account: The cooling experiments provide convincing evidence that corticothalamic feedback contributes to configuration sensitivity in MGB. The authors appropriately interpret this within a predictive coding framework. However, the mechanistic implications of the cooling results are not fully developed. In particular:

- Cooling largely abolishes enhanced deviant responses, but does not clearly enhance standard responses.

- The manuscript does not explain why removal of cortical predictions affects deviant responses selectively.

- Alternative mechanisms, such as gain control, adaptation dynamics, or changes in inhibitory balance, are not explored.

Thus, while the causal involvement of cortex is well supported, the circuit-level computation remains underspecified.

What would be needed to address this concern:

- Analyses separating changes in baseline, standard, and deviant responses.

- Tests of competing mechanistic models that could explain the observed CSI changes.

- Potentially, additional manipulations targeting specific corticothalamic pathways or layers.

These steps would go well beyond clarification or reanalysis.

In summary, this manuscript presents a compelling collection of phenomena and an impressive experimental effort. However, the central claims regarding feature-like encoding of millisecond-scale temporal configuration, hierarchical computation, and cross-species conservation are not supported by sufficient mechanistic insight or principled stimulus-response mapping. Addressing the major concerns outlined above would require substantial new experiments, theoretical development, and modeling, rather than revision of presentation or analysis alone. For this reason, I recommend rejection at this stage. That said, the dataset is rich, and with a more focused scope or a clearer mechanistic framework, parts of this work could form the basis of a strong future manuscript.

Reviewer #2: The paper is an extensive study of the coding of click trains with different types of structure in human as well as in rat auditory systems. This is a valuable dataset, and the interspecies comparison is interesting and done reasonably well given the inherent difficulties in such endeavor.

Nevertheless, the manuscript has a significant weakness. This is the claim that the authors study in some way purely temporal aspects of sound organization. While the authors manipulate purely time variables, the inevitable duality of time and frequency implies that they also modify spectral features of these sounds. See below for specific comments. The authors should at least run their stimuli through one of the ubiquitous toolboxes simulating peripheral auditory processing (e.g. https://amtoolbox.org/) to get a valid spectro-temporal description of their stimuli. For example, the CON stimulus has a strong pitch at 250 Hz and is in fact a purely harmonic stimulus composed of harmonics of a 250 Hz fundamental (all with cosine phase). The ASC and DEC stimuli have a decreasing and an increasing pitch, doing from 160 Hz to 500 Hz or back (corresponding to periods of 6 ms and 2 ms). They are constructed in the frequency domain from chirps that move appropriately in frequency, composing their individual harmonics at each point in time. The random stimuli have similar spectro-temporal interpretation.

I would urge the authors to reduce the scope of their claims (see the major comment on the discussion). There is no 'novel acoustic feature of sounds' - they study a range of time variations that have been previously studied in some detail. The beauty of this manuscript is in the extensive manipulations and the range of parameters used here, as well as with the understanding that it develops about early processing of these sounds using the rat data. I think this is sufficient novelty, no need to make insubstantial and wrong claims.

Major comments:

Stimuli: the stimuli differ in their short-term spectral structure, and this is reflected also in their neurogram representation. Thus, the fact that they are discriminable is not only due to their temporal structure, but also to the way nearby clicks interact in the spectral domain. Similar effects have been described in other perceptual phenomena that are related to fast click trains, such as precedence effects (where the click representing a direct sound and the click representing an echo may interact monaurally and spectrally; one such paper in humans is Verhulst, Bianchi and Dau 2013, but there are many, starting with the work of Hartung and Trahiotis in the early 2000's, as far as I remember). While this doesn't reduce the importance of the data studied here, it does require a re-interpretation of the results - this is far from being a purely temporal effect. Claims such as (p. 7, end of paragraph) 'only the millisecond-scale temporal configuration differed' are wrong - such click trains by necessity create also spectral features.

Behavioral test: The description is unclear, both in the main text and in the Methods. Where all types of sequences compared in a single block or each in its own block? I'm not sure that 50% is the correct random choice in this task - according to the Methods, there were 7 pairs of identical sounds and 13 of different sounds, so that chance probability was 7/20=35%. This may account for the <50% performance in the 'hard' conditions (TW10-Tw30 and the other cases at the left side of Fig. 1C).

In the methods (p. 27) the 'passive listening sessions' section starts by 'Following two active discrimination sessions'; however, in the previous section, only a single active session is described ('Active Listening Session 1'). If the active session was repeated twice, this should be clearly stated.

P. 10-11, discussion of Fig. 2: Since the sequences used as 'standard' and 'deviant' are different in Fig. 2C-F, it is not surprising that the evoked responses are different. What is the point here? Later in the text, it seems that the authors interpret the higher amplitude responses to the rare stimuli as mismatch negativity (MMN). However, MMN is usually expressed as a negative deflection in the ERP while here the deflection is positive - what is going on? Finally, combining both comments, to show that the difference between standard and deviants are MMN, it is necessary to compare the responses to the same physical sound in the two roles (this is standard in the animal physiology field - see e.g. Parras et al. 2017 and many other papers from the Malmierca lab, Geffen lab, Nelken lab; also in this manuscript, Figs. 4-7). This is possible, but has not been done, in the data in Figs. 2G-H. I would start to describe the data with these panels, and then switch to the 'multi-deviant' paradigm of Fig. 2A-F. See also Näätänen, R., Pakarinen, S., Rinne, T., & Takegata, R. (2004). The mismatch negativity (MMN): Towards the optimal paradigm. Clinical Neurophysiology, 115(1), 140-144. https://doi.org/10.1016/j.clinph.2003.04.001 for a similar paradigm.

P. 11-12, Fig. 3: These are data from an animal model, I would expect the authors to keep to the standards of the field. In auditory cortex, different stimuli are expected to elicit different responses. Thus, I frankly don't understand the point of the figure - what is the message?

Furthermore, in animal studies, responses to deviants are usually larger than responses to standards, but here in essentially all panels it's the reverse - the stimulus that is presumably standard gives rise to larger responses than the deviants. This is incompatible both with previous animal studies, with the claim that these are MMN-like responses, and with the reports of unit recordings in rats in this manuscript.

The unit recordings in Figs. 4-6 were performed using the standard approach of comparing the responses to the same stimuli in different conditions, and therefore their interpretation is easier. As expected, deviant responses tend to be somewhat larger than standard responses (controlling for stimulus identity). One comment - in Figs. 5A,B,C,F,G, one stimulus shows std>dev (peak blue above red) and the other dev>std (peak blue below red). Thus, in Fig. 5A, RN10, the peak standard response is larger than the peak deviant response but for the TW50 the reverse occurs, and so on in the other panels. This is expressed also in the lower panels, where there are significant number of neurons in which the early responses show std>dev (deep blue). This suggests that at least some of the effects are non stimulus-specific (e.g. Harpaz et al. 2021 already referred in this manuscript, who provide some discussion of non stimulus-specific effects that can mask as stimulus-specific differences). Such effects may contaminate some of the unit data presented here.

P. 17, Discussion of Fig. S2. I don't accept the conclusion that this shows that IC has reduced ability to discriminate temporal configurations. It could be that IC neurons show strong SSA to only a specific subset of all stimuli - this is implied by the selection procedure of the units studied in this analysis (those who showed large CSI for the complex tone stimuli). Thus, the comparison here is between average over all recorded data (for the click train) and highly selected subset of neurons (for the complex tone stimuli). To argue as they do, the authors have to show that NO SUBSET of neurons in IC show high level of CSI to click trains, and this is obviously wrong (e.g. Fig. 5D, where there is a substantial population of IC neurons with high CSI for two configurations of click trains).

P. 17-8, Fig. 6: these results are strangely inconsistent with previous results of the same group (Antunes and Malmierca 2011), where cortical cooling resulted also in a significant and consistent reduction of firing rates in the MGB. While I don't doubt the data, this requires an explanation. Maybe cooling was less effective? But then the interpretation of the smaller CSI during cooling is in doubt - maybe this is only due to habituation of the responses (as in Harpaz et al. 2021 )?

P. 19, Fig. 7: For this analysis to be valid, the neuropixel electrodes had to be introduced strictly perpendicular to the surface, so that the neurons from each layer were sampled from the same column - otherwise , if the electrodes were slanted, different layers were sampled at different locations in auditory cortex, and the difference in CSI magnitude could be due to that rather than to the layer composition (this is an old and consistent finding - CSIs are not uniform on the cortical surface; it goes back as far as the initial reports on such effects, e.g. Ulanovsky et al. 2004 in J. Neurosci.). The authors must have the relevant data (histological reconstructions of their Neuropixels tracks).

Discussion: There are some fundamental errors of fact and interpretation here. Temporal structure at the ms range is encoded both temporally and spectrally. For example, a click train with a 4 ms interval has an extremely strong pitch at 250 Hz, and such click trains and their modifications have been studied in great details as part of studies of pitch. The authors ignore this literature completely. Thus, the novelty of the current study is certainly not in using short-interval click trains as auditory stimuli. Also, the ability of listeners to discriminate between such sequences, as shown here in Fig. 2, is not in itself surprising - different stimuli evoke different responses, and these click trains evoke very distinctive response patterns in the auditory nerve array. Thus, the 'new feature of sound' (P. 21) aspect of this study is likely over-played. Similarly, in the paragraph spanning P. 21 and 22, '…click trains offer a particularly useful approach, because they allow temporal configuration to be manipulated while largely controlling other acoustic dimensions.' Is wrong, because of the spectral features that are inherent in the use of such click trains (with intervals that are shorter than the typical ringing time of the basilar membrane, creating spectral features in the responses). Just the fact that you are capable of manipulating easily the intervals doesn't mean that you have an independent perceptual feature. This comment is pertinent to further places in the discussion where the authors discuss the 'purely temporal' manipulations they presumably used.

Minor comments

Top of P. 7, description of the stimuli: the nature of the individual event used as click is undefined. Was it a single sample of the digital waveform? If so, what was the sampling rate used?

P. 8, success rate in the 'easy' conditions is reported with too many significant digits (the error bars are much larger than 0.001%). Two significant digits would be sufficient.

P. 14, middle paragraph: The A1 neuron in Fig. 4F showed similar responses to standards and deviants, with slight advantage to deviants. It shouldn't be described as having 'robust responses to deviants' without reference to its robust responses to standards.

Fig. 5, lower (color) panels of A-C and on: I found them confusing. Maybe show scatter plots of the spike counts that serve for the t-tests reported in the text, rather than the time-resolved firing rate differences which are affected by many stimulus-dependent and independent factors.

P. 16, last few words are corrupted. Similarly Middle of the top paragraph of P. 17, (S2 Fig.) is reversed in many references to the supp info figure. This is so also in other places in the manuscript.

Methods, P. 32, complex tones: These are not 'harmonic' - harmonic complexes have frequencies that are, well, harmonics of a fundamental (so equal differences in frequency). The tone complexes used here had components that were equally spaced on a logarithmic scale (equal ratios).

Reviewer #3: Reviewer comments:

This study investigates whether listeners can discriminate distinct patterns of rapidly presented temporal clicks and the neural mechanisms that may underlie such discrimination. The authors first show that listeners can discriminate click patterns with the same average inter-click interval (ICI) even when presented at a rapid rate such that the clicks fuse into a single percept. They then show that temporal pattern deviants produce an MMN in human EEG recordings, as well as in ECoG recordings in rat auditory cortex. They use an SSA paradigm to investigate these responses via extracellular recording in the inferior colliculus, medial geniculate, and auditory cortex. They use laminar probes to investigate layer-wise organization in the cortex and cortical cooling to investigate the impact of top-down cortico-thalamic effects.

Overall, this is a nice study. The question being addressed is novel and significant. The methods are generally high quality. The paper is clearly written and easy to read.

Major comments & critiques:

As far as I can tell, it is not possible to listen to the sounds. I, for example, could not find a link that would enable me to listen to the different click trains presented in the study. I do not think it is possible to thoroughly review this manuscript without being able to listen to the sounds.

Discrimination performance cannot be explained by the mean inter-click interval or the spectrum, since these are the same. But there is nothing in the study that addresses what acoustic statistics drive listeners' performance. Can the results be explained by a measure of temporal regularity related to pitch perception? Could a temporal modulation filterbank with asymmetric temporal tuning (e.g., fast onset, slow offset) explain this? Being able to listen to the sounds would be very helpful in generating hypotheses.

All of the neural sensitivity measures are based on deviance/SSA, which seems odd to me. The most basic question one might ask is whether the neural populations being studied can discriminate the sounds tested, analogous to their initial behavioral study. It's interesting to examine SSA, but it feels to me like the authors are missing a step.

The behavioral forced choice accuracy is a little hard to interpret because the reported measures are dependent on listeners' criterion (explaining below-chance accuracy). It would be useful to have a criterion-free measure of sensitivity, which could be compared with their neural data.

The analysis where they select units in IC that show SSA for harmonic tones is nice. This analysis should be cross-validated, however, to make sure they are not just picking up on noise. Please use a subset of data to select the units and then make sure they still show enhanced SSA for harmonic tones in left out data.

I think one possible explanation of their findings is that SSA is more general in the cortex, i.e., neurons show adaptation for a wider range of sounds. I don't feel that the authors need to rule out this explanation, but I think it should be explicitly acknowledged in the Discussion.

As far as I can tell, there is no mention of what nucleus they are recording from in the IC or MGB. Prior SSA studies, I believe, have shown big differences between nuclei, so this seems important to engage with.

It is not always clear what the sample dimension is over which they are computing statistics. For example, if you are bootstrapping a statistic, it needs to be clear what you are bootstrapping. The same goes for a t-test.

Minor notes:

When plotting images where 0 is meaningful (e.g., Figure 5), the authors should use a diverging colormap that makes it easy to see when values are below or above 0 (e.g., blue(-1):white(0):red(1)).

I think the authors should give an example of TW Figure 1, as they do for other stimuli. Please also define the acronym at first usage.

# Author comment: Author response to Decision Letter 2

**DOI:** 10.1371/journal.pbio.3004046.r003

25 May 2026

Attachment Submitted filename: Response to reviewers.docx

# Peer review: Decision Letter 2

**DOI:** 10.1371/journal.pbio.3004046.r004

**Authors:** Christian Schnell, PhD

17 Jul 2026

Dear Manolo,

Thank you for your patience while we considered your revised manuscript "Hierarchical Encoding of Click-Train Temporal Pattern in the Mammalian Auditory Pathway" for consideration as a Research Article at PLOS Biology. Your revised study has now been evaluated by the PLOS Biology editors, the Academic Editor and two of the original reviewers.

In light of the reviews, which you will find at the end of this email, we are pleased to offer you the opportunity to address the remaining points from the reviewers in a revision that we anticipate should not take you very long. We will then assess your revised manuscript and your response to the reviewers' comments with our Academic Editor aiming to avoid further rounds of peer-review, although we might need to consult with the reviewers, depending on the nature of the revisions.

You will see that Reviewer 1 is largely satisfied with the revision, but Reviewer 2 has still a lot of concerns. While their tone is a little bit harsh at some places, the comments are not unreasonable in content. Except for the auditory model, we think you will be able to address the concerns via textual revisions to keep the introduction and discussion closer to the actual manuscript and data, and to remove overstatements.

In addition to these revisions, you may need to complete some formatting changes, which you will receive in a follow up email. A member of our team will be in touch with a set of requests shortly. If you do not receive a separate email within a few days, please assume that checks have been completed, and no additional changes are required.

We expect to receive your revised manuscript within 1 month. Please email us (plosbiology@plos.org) if you have any questions or concerns, or would like to request an extension.

At this stage, your manuscript remains formally under active consideration at our journal; please notify us by email if you do not intend to submit a revision so that we withdraw the manuscript.

**IMPORTANT - SUBMITTING YOUR REVISION**

Your revisions should address the specific points made by each reviewer. Please submit the following files along with your revised manuscript:

1. A 'Response to Reviewers' file - this should detail your responses to the editorial requests, present a point-by-point response to all of the reviewers' comments, and indicate the changes made to the manuscript.

*NOTE: In your point-by-point response to the reviewers, please provide the full context of each review. Do not selectively quote paragraphs or sentences to reply to. The entire set of reviewer comments should be present in full and each specific point should be responded to individually.

You should also cite any additional relevant literature that has been published since the original submission and mention any additional citations in your response.

2. In addition to a clean copy of the manuscript, please also upload a 'track-changes' version of your manuscript that specifies the edits made. This should be uploaded as a "Revised Article with Changes Highlighted " file type.

*Resubmission Checklist*

When you are ready to resubmit your revised manuscript, please refer to this resubmission checklist: https://plos.io/Biology_Checklist

To submit a revised version of your manuscript, please go to https://www.editorialmanager.com/pbiology/ and log in as an Author. Click the link labelled 'Submissions Needing Revision' where you will find your submission record.

Please make sure to read the following important policies and guidelines while preparing your revision:

*Published Peer Review*

Please note while forming your response, if your article is accepted, you may have the opportunity to make the peer review history publicly available. The record will include editor decision letters (with reviews) and your responses to reviewer comments. If eligible, we will contact you to opt in or out. Please see here for more details:

https://blogs.plos.org/plos/2019/05/plos-journals-now-open-for-published-peer-review/

*PLOS Data Policy*

Please note that as a condition of publication PLOS' data policy (http://journals.plos.org/plosbiology/s/data-availability) requires that you make available all data used to draw the conclusions arrived at in your manuscript. If you have not already done so, you must include any data used in your manuscript either in appropriate repositories, within the body of the manuscript, or as supporting information (N.B. this includes any numerical values that were used to generate graphs, histograms etc.). For an example see here: http://www.plosbiology.org/article/info%3Adoi%2F10.1371%2Fjournal.pbio.1001908#s5

*Blot and Gel Data Policy*

We require the original, uncropped and minimally adjusted images supporting all blot and gel results reported in an article's figures or Supporting Information files. We will require these files before a manuscript can be accepted so please prepare them now, if you have not already uploaded them. Please carefully read our guidelines for how to prepare and upload this data: https://journals.plos.org/plosbiology/s/figures#loc-blot-and-gel-reporting-requirements

*Protocols deposition*

To enhance the reproducibility of your results, we recommend that if applicable you deposit your laboratory protocols in protocols.io, where a protocol can be assigned its own identifier (DOI) such that it can be cited independently in the future. Additionally, PLOS ONE offers an option for publishing peer-reviewed Lab Protocol articles, which describe protocols hosted on protocols.io. Read more information on sharing protocols at https://plos.org/protocols?utm_medium=editorial-email&utm_source=authorletters&utm_campaign=protocols

Thank you again for your submission to our journal. We hope that our editorial process has been constructive thus far, and we welcome your feedback at any time. Please don't hesitate to contact us if you have any questions or comments.

Sincerely,

Christian

Christian Schnell, PhD

Senior Editor

PLOS Biology

cschnell@plos.org

----------------------------------------------------------------

REVIEWS:

Reviewer #1: Thank you for the substantial effort invested in revising the manuscript. I have carefully read the revised manuscript, response letter, figures, and supporting information.

In my previous review, my main concern was that the manuscript made broad conceptual claims regarding feature-like encoding of millisecond-scale temporal configurations, hierarchical computation, and cross-species conservation without providing a sufficiently explicit stimulus-to-neural mapping or mechanistic account. I felt that supporting those claims in their original form would require substantial additional experiments, parametric analyses, and modeling.

The revisions address these concerns in a thoughtful and constructive manner. While the current manuscript still does not provide the missing parametric or encoding-model analyses, nor does it identify the specific acoustic or temporal statistics encoded by the auditory system, the authors have substantially narrowed the scope of their claims so that the central conclusions no longer depend on these stronger mechanistic interpretations. In particular, the manuscript no longer presents the findings as establishing a novel, purely temporal auditory feature, and it now acknowledges that the click-train manipulations necessarily produce spectrotemporal consequences. The stimulus set is also more appropriately described as an operationally defined family of click-train patterns rather than as samples drawn from a formally parameterized temporal feature space.

Several specific revisions further strengthen the study. The inclusion of a spectrally matched control is valuable, although it remains important to recognize that matching long-term spectral magnitude does not guarantee equivalent peripheral or neurogram-level representations. The revised ECoG analysis now employs a more appropriate comparison between responses to the same stimulus presented as standard versus deviant. The behavioral analyses have been corrected and supplemented with d′ measures. The IC selection analysis is now cross-validated, relevant subnuclei are specified, and the interpretation of the cortical-cooling experiment has been appropriately moderated so that predictive coding is no longer presented as the sole possible explanation.

Overall, the concerns regarding overstatement that motivated my original recommendation have largely been resolved. The manuscript is now more accurately framed as a systems-level characterization of behavioral and neural sensitivity to rapid click-train patterns, rather than as a mechanistic demonstration of a novel auditory feature.

Two limitations regarding the broader conceptual impact remain.

First, the principal neural findings continue to rely on CSI/SSA measures, namely comparisons between responses to the same stimulus when presented as a deviant versus as a standard, across IC, MGB, and A1. Such measures alone cannot distinguish genuine deviance sensitivity or prediction-error signaling from use-dependent adaptation, for example differential adaptation of narrowly tuned feedforward inputs. Resolving this issue would require explicit and falsifiable modeling approaches, such as fitting an adaptation model and testing whether deviant responses exceed model predictions. The present study does not undertake such analyses, and therefore the neural mechanisms underlying the observed effects remain open to interpretation.

Second, the study should be considered in the context of prior work. The hierarchical organization of the reported effects largely recapitulates findings previously reported by the authors' own group for frequency oddballs along the same IC-MGB-A1 pathway (Parras et al., 2017). In addition, the authors appropriately position the present work as a temporal-domain counterpart to Harpaz et al. (2021), which demonstrated stimulus-specific adaptation to configural "tone-cloud" identity that emerges strongly in cortex and only weakly in subcortical structures. This comparison helps clarify both the contribution and its limitations. Harpaz et al. employed spectrally balanced stimuli specifically designed to minimize the contribution of frequency-specific adaptation, whereas the click-train stimuli used here retain spectrotemporal differences that are only partially controlled by the long-term FFT-matched condition. Consequently, I view the present work as a valuable extension of an established line of research, expanding from spectral to temporal configurations, rather than as a fundamentally new conceptual advance.

These remaining points primarily concern the level of conceptual advance and journal-level significance rather than scientific rigor or validity. In my view, the manuscript is now scientifically sound and substantially improved.

Reviewer #2: The revised version is substantially improved. Nevertheless, I found reading the paper difficult, due to the tendency of the authors to make very wide-ranging, wrong, claims, and then do experiments that do not engage with these claims at all. The detailed comments below are full of such issues.

There are in addition some technical issues and figures that obviously show wrong data.

Problem settings and irrelevant claims: These are mostly issues in the introduction and the discussion.

P.4, top paragraph: This is mixing up temporal organizations at many time scales, which contribute to perception in different ways and are processed in different ways and different structures. For example, speech and music have strong temporal modulations at slow rates - for example, the typical rate of modulation of speech is about 4 Hz. These are very different from the time scales studied here, where interclick intervals are a few ms. This whole paragraph doesn't make sense (see e.g. among others Meibauer's 'Aspects of a theory of bullshit', https://doi.org/10.1075/pc.23.1.04mei). I would strongly urge the authors to drop the slop.

P. 4, line 9: 'the distinction between "what" a sound is and "when" it occurs' - I sincerely protest. When a sound occurs is when it occurs - this is totally independent of what a sound is. See e.g. Anne Danielsen's work on the 'p-center' (the time point to which a sound is perceptually assigned). These statements are simply nonsense.

P. 4, lines 14 and on - these statements are correct, but totally irrelevant to the data here, because they occur at very different time scales.

P. 4, lines 16-18 - this is a half-truth. We know that this information is represented in a way that allows neural discrimination - starting from the auditory nerve fibers, that provide a veridical representation of click trains at the tested rates here (interclick intervals of a few ms). We also know that they are represented in a way that allows perceptual discrimination - for example, a periodic click train has a pitch while a Poisson click train with the same average ICI doesn't. The contribution of this paper is in following this representation along the ascending auditory system, and showing that it continues to carry enough information to account for perception.

Frankly, I would simply remove this paragraph (P. 4 up to line 18) and start with the next one, which engages with the actual content of this paper.

P. 5, lines 5-9: it is known that different ms-scale temporal configuarions are perceived as distinct (e.g. periodic vs. Poisson). What is unknown is how these unitary objects are represented neurally in higher stations of the auditory system (in auditory nerve and CN, they are likely represented largely veridically by the temporal sequence of spikes).

The final paragraph of the introduction is much better. This language should be adopted also in the earlier parts of the introduction.

P. 20 L2-6: This result is not surprising - the auditory system doesn't measure the long-term spectral magnitude. Rather, it is sensitive to the short-term spectro-temporal structure. The fact that sounds with identical long-term power spectra may sound very different is very well known (e.g. the 'ramp and damp' papers of Roy Patterson). Another well-known example is the Schroeder-phase harmonic complexes, which come in two polarities (causing basically a rising or a falling chirp structure); these sounds evoke very different activities already in the inner ear, reflected in different ABR structure. So the attribution of 'contributions from peripheral non-linearities' is wrong. These sounds have different spectro-temporal structure (the members of a pair are time-reversed), and so evoke different responses.

Even in the context of CSI, this result is not novel - Harpaz et al. (referenced in the manuscript a number of times) did all their work with sounds that share the same long-term spectrum, and showed largely the same results (larger effects of deviance in A1 than in IC and MGB).

P. 24, Lines 2-4: If you do not make the claim, you don't need to apologize for making it. You should remove these lines.

Lines 7-14: back to these unsubstantiated, inexact claims. How the auditory system encodes temporal structure at the ms scale has been intensively studied, for example in the pitch literature. Such claims are wrong, but also unnecessary. Remove them from the paper. What shown here is novel and interesting enough without them.

Fine timing of water flow, bird song, and wind in trees is NOT at the ms scale. This is poetic and sounds nice, but is deeply misleading.

P. 24, L 15 and following: Same issues. Spoken language has hierarchical temporal integration, but ms scale is not one of them. For example, Regev et al. 2024 (Nat. Hum. Behav., Fedorenko's group) show temporal receptive windows of 1, 4, and 6 words, where one word is about 250 ms long, two orders of magnitude longer than the ms scale studied in this paper. You can go higher to syllabic rate, which would give you another order of magnitude, but still more like 50 ms than like 4 ms.

As in the introduction, once past these generalities, the text stabilizes at things that make sense. But I would remove these incoherent ramblings.

Paragraph spanning P. 25-6: as I repeat again and again, these sounds are perceptually discriminable because they evoke different activity in the auditory system. This is not stated anywhere, but it should be the starting point for any discussion of the discrimination between these sounds. Afterward, one can ask whether there are 'summary statistics' that can be used to discriminate between them, and here comes the list of features discussed by the authors.

This bleeds into the next paragraph, which discusses the fact that neural signals were more discriminable than human behavior. There is no problem with this - behavior is a function of the neural signals, and therefore, you expect neural representations to be at least as fine as behavior, but they can be finer. There is no real 'dissociation' here.

Paragraph spanning P. 27-8: the fact that there is SSA to spectrally matched stimuli has already been shown (Harpaz et al.); I think this should be mentioned in this paragraph. As they argued, this fact falsifies mechanisms that occur within channel; they are however definitely not falsifying any feedforward mechanisms, which may be sensitive to spectro-temporal differences. I would remove this from the paper - it's wrong, but also unimportant for the main thrust of the results.

Technical issues that need to be corrected:

The description of stimulus synthesis is much better now. Thanks for making the sound files available. However, spectro-temporal analysis is not described in detail (just in passing in a few lines spanning P. 7-8, with a wrong reference to the supplementary information file - this is not the name under which it is provided) and is wrong the way it is provided. The analysis is clearly on a linear frequency - you can see the linearly-spaced harmonics in some of the spectrograms - but the frequency axis shown on the right hand side of each panel is logarithmic. When performing time-frequency analysis, the windows that have been used must be specified. I suggest that the authors use one of the simulations of the peripheral auditory system for that purpose - there are many good ones around (https://amtoolbox.org/, for example; I had that cited already in the previous review).

Figure 1D: something wrong in the calculation of the d'. While TW10-TW30 performance is at chance level (Fig. 1C), the d' values center on 1. But d' of 1 corresponds to a fair bias towards correct responses.

I think the d' it was computed using the standard equations (transformed hit rates - transformed FA rates), which assume equal probability classes, not taking into account the fact that the test was conducted with 35% chance for same pairs.

P. 14, Fig. 3C and text: '…both showed enhanced negative-going responses when presented as deviants…' - I frankly don't see it. The red lines, if anything, are above the blue lines. If there's anything that I miss, please show that explicitly in the figures.

I already commented on that in the previous review. As presented, the data in Fig. 3 do not support the text that describe them. Some red lines are above the blue lines, some are below; in some the peak-to-peak seems to be larger for deviants than for standards, and some the reverse.

I would accept these data as evidence that the stimuli are represented differentially somewhere along the pathway, but I would remove the text about 'enhanced responses to deivants'. Frankly, I don't see anything consistent about that.

Other comments, largely in the order in which they occur in the text:

P. 7 and Table S1: Note that the 'Asc' stimulus has a descending pitch, and the 'Dec' stimulus has an ascending pitch. This confused me at first.

P. 9 L6: drop 'psychological' - you likely mean 'behavioral'. Also, on the same page, 'reference proportion' is incorrect ('chance level' or something like that; at least, say 'probability' rather than 'proportion').

P. 9, L. 15-16: you only know that the discriminated the stimuli, you don't know what features they used for that. Drop the line about the participants recognizing the objects as distinct entities ('distinct' and 'different' are not the same thing).

P. 10, l4-5: the authors correctly state that the behavioral results do not determine the specific features that were used for discrimination, implying (at least to me) that they are going to do that. However, they do something completely different - they study the neural representations of these sounds. Remove the sentence 'However, the present behavioral data…'.

P 10 L 7-10: There's a subtle point here that is obscured by all the enveloping text. '…click-train patterns … differentially represented in neural activity' requires a different experiment than the use of oddball paradigms. In the same auditory station, different click train patterns can evoke the same neural activity, and yet also evoke differential deviant-standard responses. Oddball sequences demonstrate that over the whole pathway creating the responses, the two stimuli are represented differentially; not that the recorded neural response represents these stimuli differentially. It's completely fine that the authors used oddball design, and I'm very happy with their results, but yet again they shouldn't state things they didn't do.

Pp. 10-12, EEG recordings: P. 10 l7 and on, and also Fig. 2A, seems to imply that in the oddball sequences only TW30 was used as standard. However, later on in the text it turns out that in some recordings pairs of sounds were used alternatively as standard and deviant (Fig. 2E and 2F), and the in the multi-deviant sequences, sometimes RN10 was used as the standard. Please explain exactly what you did.

Paragraph spanning Pp. 10 and 11 - why did you select these 27 channels and no others?

P. 12 l. 7: …when the deviants were Asc, …. etc.' - remove the etc., and state exactly which conditions.

P. 12 L. 21, again 'psychological' instead of 'behavioral'.

P. 18 L11 and other places: the reference to the supplemental figures is stragne ('…are summarized in S1 Fig.' and similar texts elsewhere.

Fig. 5: this figures incidentally shows that at least in IC, different click trains indeed evoke different responses! This is obvious in 5C, 5H and 5M (line graphs). This is actually an interesting point: whereas in IC different stimuli give rise to different responses (and therefore can be discriminated by the actual responses), in MGB and A1 potentially these differences are reduced, but the two areas are still sensitive to the identity of the stimuli as shown by the deviant-standard contrast.

P. 19, L6-8: I don't fully follow the logic, and the new text, while much clearer than the original version, still doesn't convince me. The CSI is a joint property of the neuron and of the stimuli used to test it. The units used in this analysis were selected by their large CSI on the complex tone, so why does the fact that the weaker click-train CSI in IC cannot be explained solely by the generally weaker SSA in IC? It could be that there is another subset of neurons with very large CSI for the click-trains.

What this controls show is that large CSI is, well, a joint property of the tested neuron and the tested stimulus. Large CSI for one stimulus does not imply large CSI for other stimuli. I frankly don't see anything else here.

# Author comment: Author response to Decision Letter 3

**DOI:** 10.1371/journal.pbio.3004046.r005

17 Aug 2026

Attachment Submitted filename: Response to Comments_260808.docx

# Peer review: Decision Letter 3

**DOI:** 10.1371/journal.pbio.3004046.r006

**Authors:** Christian Schnell, PhD

11 Sep 2026

Dear Manolo,

Thank you for your patience while we considered your revised manuscript "Hierarchical Encoding of Click-Train Temporal Pattern in the Mammalian Auditory Pathway" for publication as a Research Article at PLOS Biology. This revised version of your manuscript has been evaluated by the PLOS Biology editors, the Academic Editor and one of the original reviewers.

Based on the reviews and on our Academic Editor's assessment of your revision, we are likely to accept this manuscript for publication, provided you satisfactorily address the following data and other policy-related requests:

* We would like to suggest a different title to improve its accessibility for our broad audience:

Distinct millisecond-scale click-train patterns evoke discriminable behavioral and neural responses in humans and rats

* Please add the links to the funding agencies in the Financial Disclosure statement in the manuscript details.

* Please include information in the Methods section whether the study has been conducted according to the principles expressed in the Declaration of Helsinki.

* DATA POLICY:

You may be aware of the PLOS Data Policy, which requires that all data be made available without restriction: http://journals.plos.org/plosbiology/s/data-availability. For more information, please also see this editorial: http://dx.doi.org/10.1371/journal.pbio.1001797

Note that we do not require all raw data. Rather, we ask that all individual quantitative observations that underlie the data summarized in the figures and results of your paper be made available in one of the following forms:

1) Supplementary files (e.g., excel). Please ensure that all data files are uploaded as 'Supporting Information' and are invariably referred to (in the manuscript, figure legends, and the Description field when uploading your files) using the following format verbatim: S1 Data, S2 Data, etc. Multiple panels of a single or even several figures can be included as multiple sheets in one excel file that is saved using exactly the following convention: S1_Data.xlsx (using an underscore).

2) Deposition in a publicly available repository. Please also provide the accession code or a reviewer link so that we may view your data before publication.

Regardless of the method selected, please ensure that you provide the individual numerical values that underlie the summary data displayed in the following figure panels as they are essential for readers to assess your analysis and to reproduce it: 1CD, 5EJO, 7BDFH, S3CFIL and S4H

NOTE: the numerical data provided should include all replicates AND the way in which the plotted mean and errors were derived (it should not present only the mean/average values).

Please also ensure that figure legends in your manuscript include information on where the underlying data can be found, and ensure your supplemental data file/s has a legend.

Please ensure that your Data Statement in the submission system accurately describes where your data can be found.

* CODE POLICY

Per journal policy, if you have generated any custom code during the course of this investigation, please make it available without restrictions. Please ensure that the code is sufficiently well documented and reusable, and that your Data Statement in the Editorial Manager submission system accurately describes where your code can be found. More information on our Code Policy, what and how to share can be found here: https://journals.plos.org/plosbiology/s/code-availability

Please note that we cannot accept sole deposition of code in GitHub, as this could be changed after publication. However, you can archive this version of your publicly available GitHub code to Zenodo. Once you do this, it will generate a DOI number, which you will need to provide in the Data Accessibility Statement (you are welcome to also provide the GitHub access information). See the process for doing this here: https://docs.github.com/en/repositories/archiving-a-github-repository/referencing-and-citing-content

As you address these items, please take this last chance to review your reference list to ensure that it is complete and correct. If you have cited papers that have been retracted, please include the rationale for doing so in the manuscript text, or remove these references and replace them with relevant current references. Any changes to the reference list should be mentioned in the cover letter that accompanies your revised manuscript.

In addition to these revisions, you may need to complete some formatting changes, which you will receive in a follow up email. A member of our team will be in touch with a set of requests shortly. If you do not receive a separate email within a few days, please assume that checks have been completed, and no additional changes are required.

We expect to receive your revised manuscript within two weeks.

To submit your revision, please go to https://www.editorialmanager.com/pbiology/ and log in as an Author. Click the link labelled 'Submissions Needing Revision' to find your submission record. Your revised submission must include the following:

- a cover letter that should detail your responses to any editorial requests, if applicable, and whether changes have been made to the reference list

- a Response to Reviewers file that provides a detailed response to the reviewers' comments (if applicable, if not applicable please do not delete your existing 'Response to Reviewers' file.)

- a track-changes file indicating any changes that you have made to the manuscript.

NOTE: If Supporting Information files are included with your article, note that these are not copyedited and will be published as they are submitted. Please ensure that these files are legible and of high quality (at least 300 dpi) in an easily accessible file format. For this reason, please be aware that any references listed in an SI file will not be indexed. For more information, see our Supporting Information guidelines:

https://journals.plos.org/plosbiology/s/supporting-information

*Published Peer Review History*

Please note that you may have the opportunity to make the peer review history publicly available. The record will include editor decision letters (with reviews) and your responses to reviewer comments. If eligible, we will contact you to opt in or out. Please see here for more details:

https://plos.org/published-peer-review-history/

*Press*

Should you, your institution's press office or the journal office choose to press release your paper, please ensure you have opted out of Early Article Posting on the submission form. We ask that you notify us as soon as possible if you or your institution is planning to press release the article.

*Protocols deposition*

To enhance the reproducibility of your results, we recommend that if applicable you deposit your laboratory protocols in protocols.io, where a protocol can be assigned its own identifier (DOI) such that it can be cited independently in the future. Additionally, PLOS ONE offers an option for publishing peer-reviewed Lab Protocol articles, which describe protocols hosted on protocols.io. Read more information on sharing protocols at https://plos.org/protocols?utm_medium=editorial-email&utm_source=authorletters&utm_campaign=protocols

Please do not hesitate to contact me should you have any questions.

Sincerely,

Christian

Christian Schnell, PhD

Senior Editor

cschnell@plos.org

PLOS Biology

------------------------------------------------------------------------

Reviewer remarks:

Reviewer #2: The authors have responded very satisfactorily to all my concerns.

# Editor report: Decision Letter 4

**DOI:** 10.1371/journal.pbio.3004046.r007

**Authors:** Christian Schnell, PhD

24 Sep 2026

Dear Manuel,

Thank you for the submission of your revised Research Article "Distinct millisecond-scale click-train patterns evoke discriminable behavioral and neural responses in humans and rats" for publication in PLOS Biology. On behalf of my colleagues and the Academic Editor, Jennifer Bizley, I am pleased to say that we can in principle accept your manuscript for publication, provided you address any remaining formatting and reporting issues. These will be detailed in an email you should receive within 2-3 business days from our colleagues in the journal operations team; no action is required from you until then. Please note that we will not be able to formally accept your manuscript and schedule it for publication until you have completed any requested changes.

Please take a minute to log into Editorial Manager at http://www.editorialmanager.com/pbiology/, click the "Update My Information" link at the top of the page, and update your user information to ensure an efficient production process.

PRESS

We frequently collaborate with press offices. If your institution or institutions have a press office, please notify them about your upcoming paper at this point, to enable them to help maximize its impact. If the press office is planning to promote your findings, we would be grateful if they could coordinate with biologypress@plos.org. If you have previously opted in to the early version process, we ask that you notify us immediately of any press plans so that we may opt out on your behalf.

We also ask that you take this opportunity to read our Embargo Policy regarding the discussion, promotion and media coverage of work that is yet to be published by PLOS. As your manuscript is not yet published, it is bound by the conditions of our Embargo Policy. Please be aware that this policy is in place both to ensure that any press coverage of your article is fully substantiated and to provide a direct link between such coverage and the published work. For full details of our Embargo Policy, please visit http://www.plos.org/about/media-inquiries/embargo-policy/.

Thank you again for choosing PLOS Biology for publication and supporting Open Access publishing. We look forward to publishing your study.

Sincerely,

Christian

Christian Schnell, PhD

Senior Editor

PLOS Biology

cschnell@plos.org
